# Molecular cleft or tweezer compounds derived from trioxabicyclo[3.3.1]nonadiene diisocyanate and diacid dichloride

**DOI:** 10.3762/bjoc.11.1

**Published:** 2015-01-02

**Authors:** Gert Kollenz, Ralf Smounig, Ferdinand Belaj, David Kvaskoff, Curt Wentrup

**Affiliations:** 1 Institute of Chemistry, Karl-Franzens University of Graz, Heinrichstrasse 28, A-8010 Graz, Austria; 2 School of Chemistry and Molecular Biosciences, The University of Queensland, Brisbane, Qld 4072, Australia

**Keywords:** carbamates, crown ethers, diisocyanate, isocyanate, ureas

## Abstract

The structures of two derivatives of the bisdioxine diisocyanate **1**, the bisurea **4** and the biscarbamate **5**, are established by X-ray crystallography and DFT calculations. These compounds possess *endo*,*endo* structures, in the case of the bisurea **4** with two nearly parallel pendant chains. The X-ray structures are reproduced very well by DFT calculations. Similar *endo*,*endo* conformations are calculated for the bisamide crown ether derivatives **7**, where two proximate and nearly parallel crown ether units endow the molecules with a claw-like molecular cleft or tweezer structure as evidenced by an enhanced ability to extract some alkali, alkaline earth and rare earth metal ions.

## Introduction

**Figure 1:**
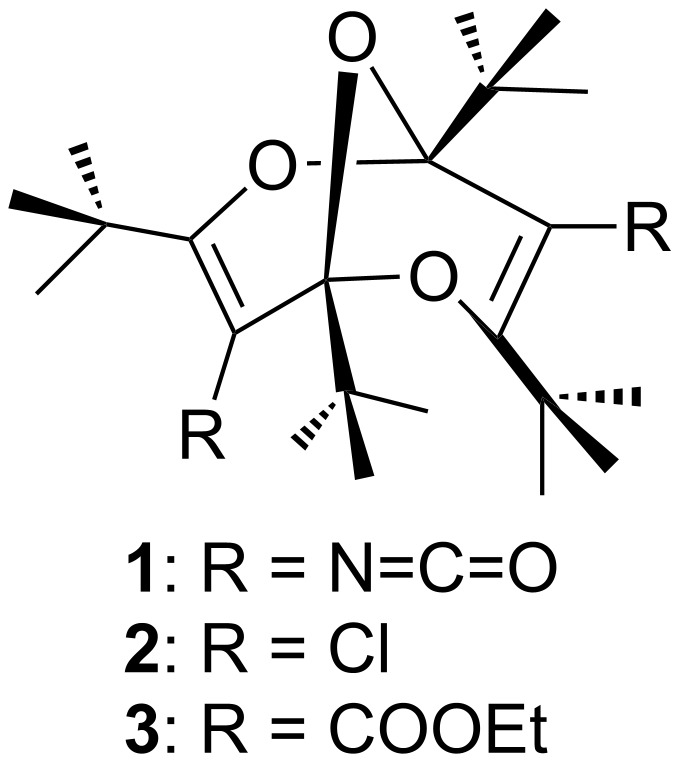
2,6,9-Trioxabicyclo[3.3.1]nonadiene (“bisdioxine”) derivatives.

The synthesis of the surprisingly stable, monomeric diisocyanate **1** (Figure 1) was reported recently [1]. This and several other derivatives of the unique 2,6,9-trioxabicyclo[3.3.1]nonadiene (“bisdioxine”) system, including the diacid, the diacid dichloride **2** [2] and the diethyl ester **3** [3] are readily synthesized from the stable dimer of dipivaloylketene.

**Figure 2:**
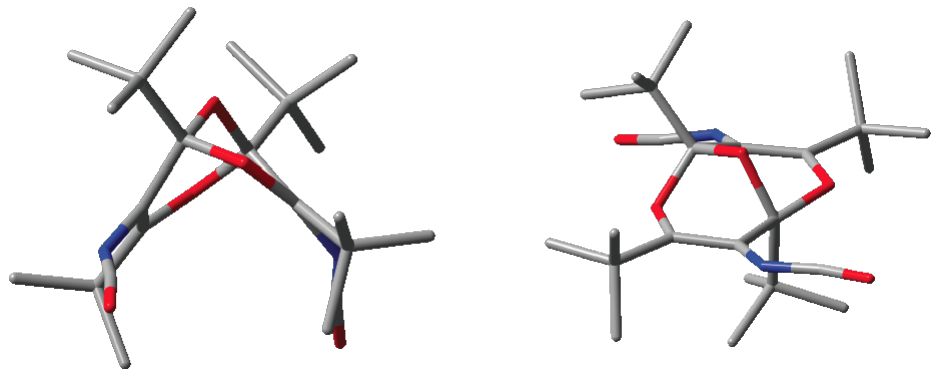
B3LYP/6-31G**-calculated structures of the stable *endo*,*endo* diisocyanate **1** (left) and the unstable *endo*,*exo* isomer (right) (hydrogen atoms are omitted for clarity).

**Scheme 1:**
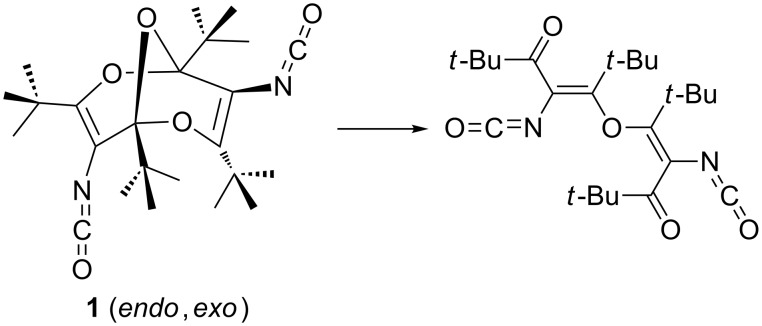
Ring opening taking place on attempted optimization of the calculated, putative *endo*,*exo* isomer of **1**.

DFT calculations at the B3LYP/6-31G** level predict that molecules of this type can exist only in the *endo*,*endo* forms shown, i.e., with the functional groups pointing “downwards”, away from the central ether bridge [1]. The calculated *endo*,*endo* and *endo*,*exo* structures of **1** are shown in Figure 2. However, neither the *endo*,*exo* structure, nor the *exo*,*exo* isomer (not shown) represent stable energy minima [1]. Optimization of the *endo*,*exo* structure leads to ring opening to a new diisocyanate (Scheme 1). Therefore, it is of some importance to ascertain the actual molecular structures of compounds of this type by X-ray crystallography.

Here we report the X-ray crystal structures of two derivatives of the diisocyanate **1**, viz. the di(hexylurea) **4** and the di(methyl carbamate) **5**, as well as the calculated structures of diamide derivatives **7**.

## Results and Discussion

### Diurea and dicarbamate

**Scheme 2:**
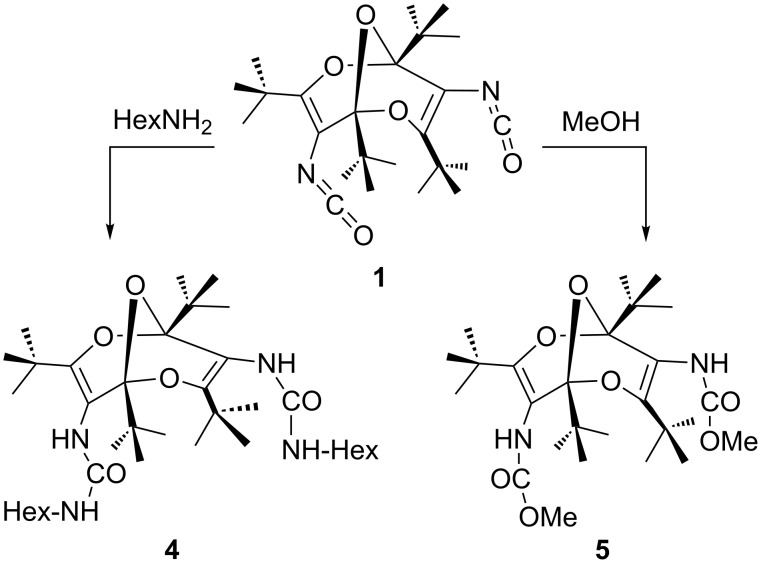
Synthesis of the di(hexylurea) and di(methyl carbamate) derivatives **4** and **5**.

**Figure 3:**
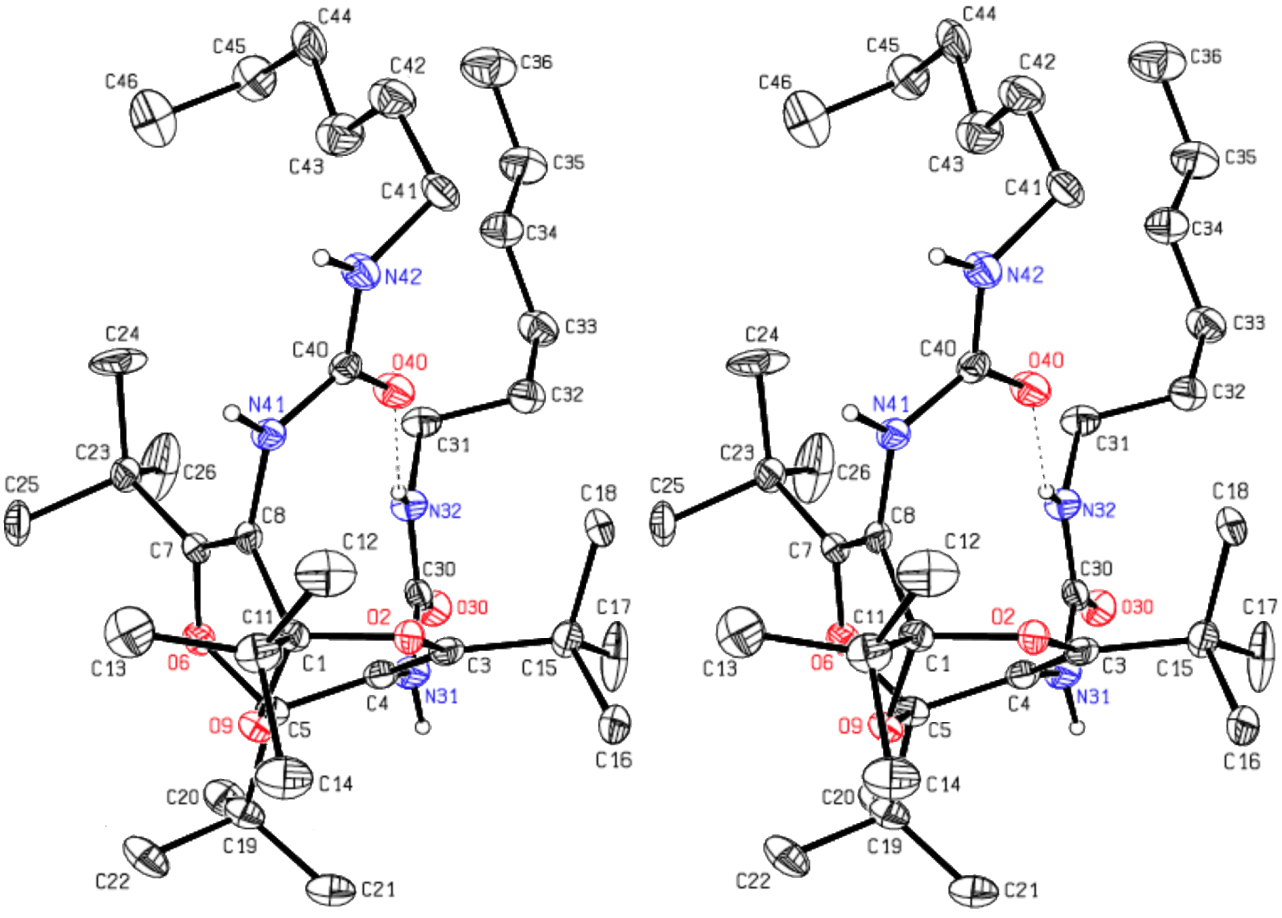
Stereoscopic ORTEP plot of molecule **A** of the di(hexylurea) derivative **4** with atomic numbering scheme. The ellipsoids are drawn at the 50% probability level. The H atoms bonded to N are drawn with arbitrary radii; the other H atoms as well as the disordered atoms with site occupation factors less than 0.5 were omitted for the sake of clarity. The intramolecular hydrogen bond is indicated by a dashed line (H(N32)–O40 = 1.970 Å).

**Figure 4:**
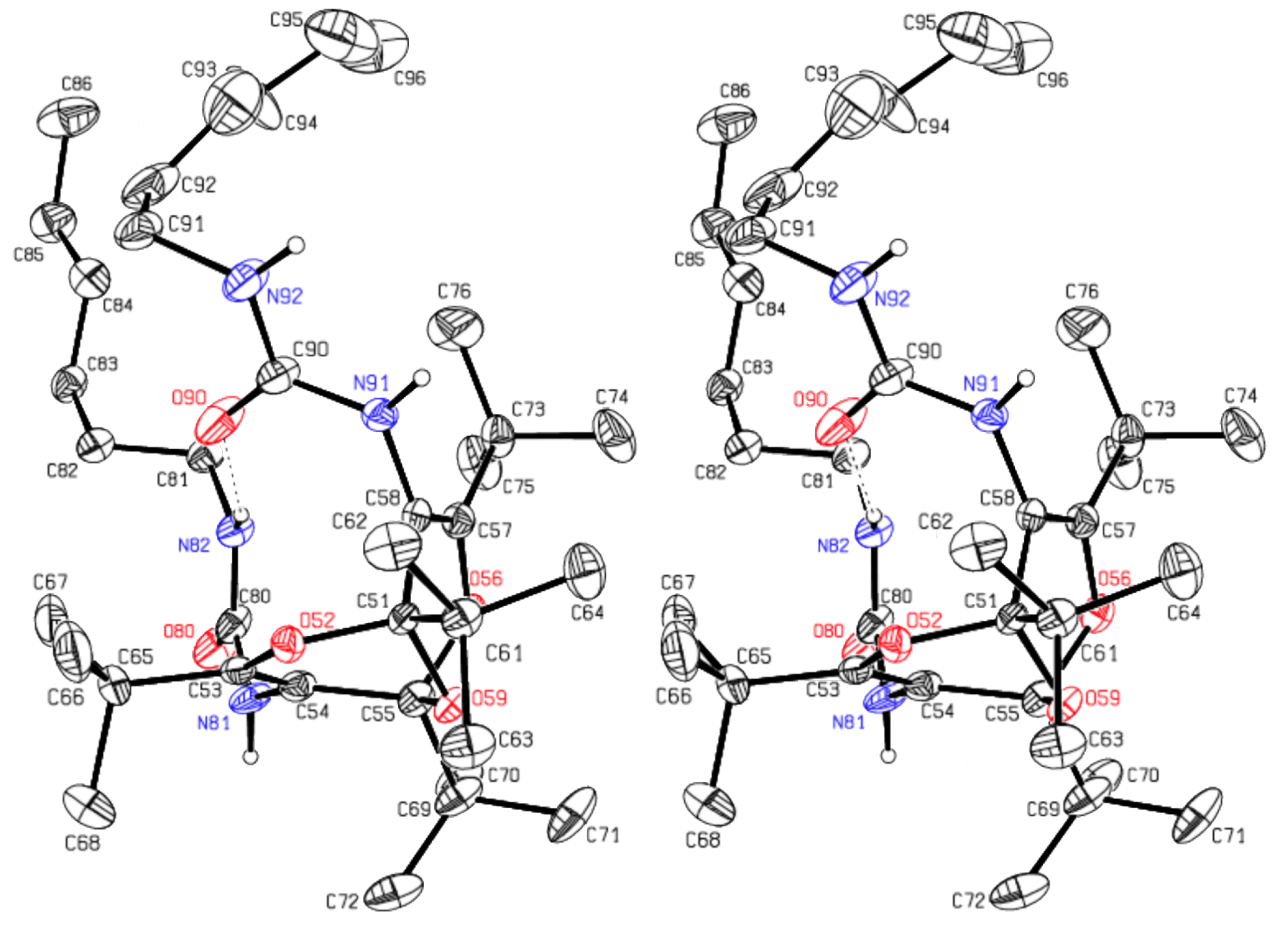
Stereoscopic ORTEP plot of molecule **B** of **4** with atomic numbering scheme. The ellipsoids are drawn at the 50% probability level. The H atoms bonded to N are drawn with arbitrary radii; the other H atoms as well as the disordered atoms with site occupation factors less than 0.5 were omitted for reasons of clarity. The intramolecular hydrogen bond is indicated by a dashed line (H(N82–O90) = 2.006 Å).

The di(hexylurea) **4** and the di(methyl carbamate) **5** were synthesized by addition of hexylamine and methanol, respectively, to diisocyanate **1** (Scheme 2) [1]. The crystal structure analysis of **4** confirmed the compound as 1,3,5,7-tetra-*tert*-butyl-2,6,9-trioxabicyclo[3.3.1]nona-3,7-diene-4,8-diyl-bis(3-hexylurea). All atoms lie on general positions. The asymmetric unit consists of two molecules, A and B (see Figure 3 and Figure 4) related by a pseudo-inversion center. A refinement with only one molecule in a unit cell with a’ = a/2 – corresponding to the fact that reflections with *h* odd [*I*_max_ 12.74(38)] are far weaker (but still significant) than those with *h* even [*I*_max_ 1000(27)] – resulted in an much higher R value, R1 = 0.0942. In each molecule there is one H atom (H31, H81) bonded to the nitrogen atom, which is *cis* to the O atom of the urea subunit. These H atoms are not involved in hydrogen bonding. The other H atoms of the same urea subunit (H(N32) and H(N82)) show intramolecular hydrogen bonds to O40 and O90 of 1.970 and 2.006 Å, respectively (dashed lines in Figure 3 and Figure 4). The four other H atoms bonded to N show intermolecular hydrogen bonds forming chains parallel to the [1] direction.

**Figure 5:**
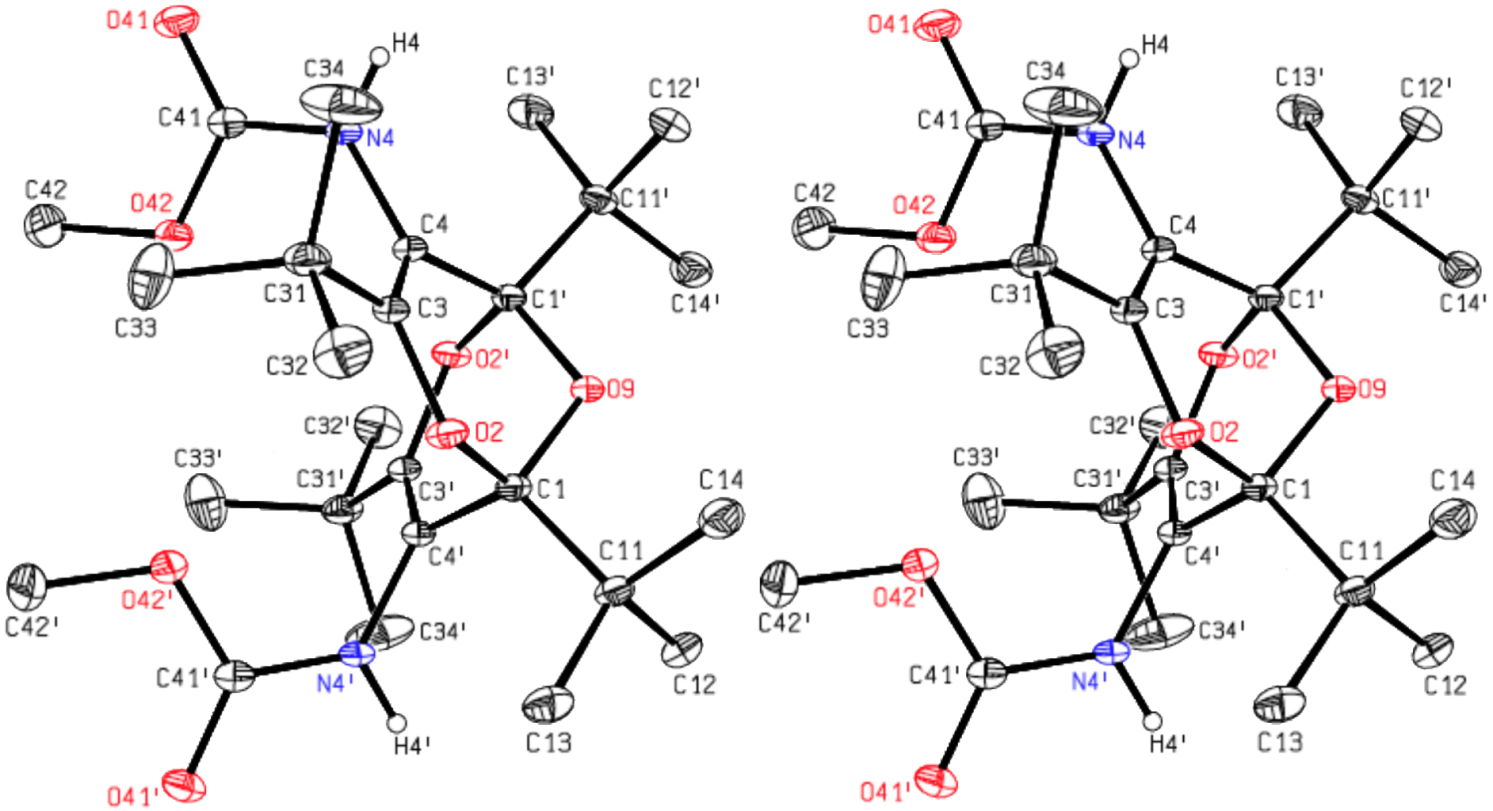
Stereoscopic ORTEP plot of the di(methyl carbamate) **5** showing the atomic numbering scheme. The ellipsoids are drawn at the 50% probability level. The H atoms bonded to N are drawn with arbitrary radii; the H atoms of the methyl groups were omitted for reasons of clarity.

The analogous crystal structure analysis of **5** confirmed this compound as dimethyl (1,3,5,7-tetra-*tert*-butyl-2,6,9-trioxabicyclo[3.3.1]nona-3,7-diene-4,8-diyl)biscarbamate. The molecules are ordered around two-fold rotation axes through O9 (Figure 5). Each molecule is connected by a donor and an acceptor hydrogen bond to each of two adjacent molecules. Thereby chains parallel to the [101] direction are formed (see Figure S1 in Supporting Information File 1).

It is seen in the crystal structures depicted in Figures 3–5 that compounds **4** and **5** (and therefore also **1**) exist in the *endo,endo* structures, and each functional group is surrounded by three *tert*-butyl groups, which direct these functional groups away from the ether bridge. This ascertains that the functional groups with their attachments are oriented essentially parallel, i.e., away from the concave bisdioxine backbone. Furthermore, the intramolecular hydrogen bonds depicted in Figure 3 and Figure 4 ascertain that the two pendant hexylamino chains in **4** are held in close proximity.

### Calculations

**Figure 6:**
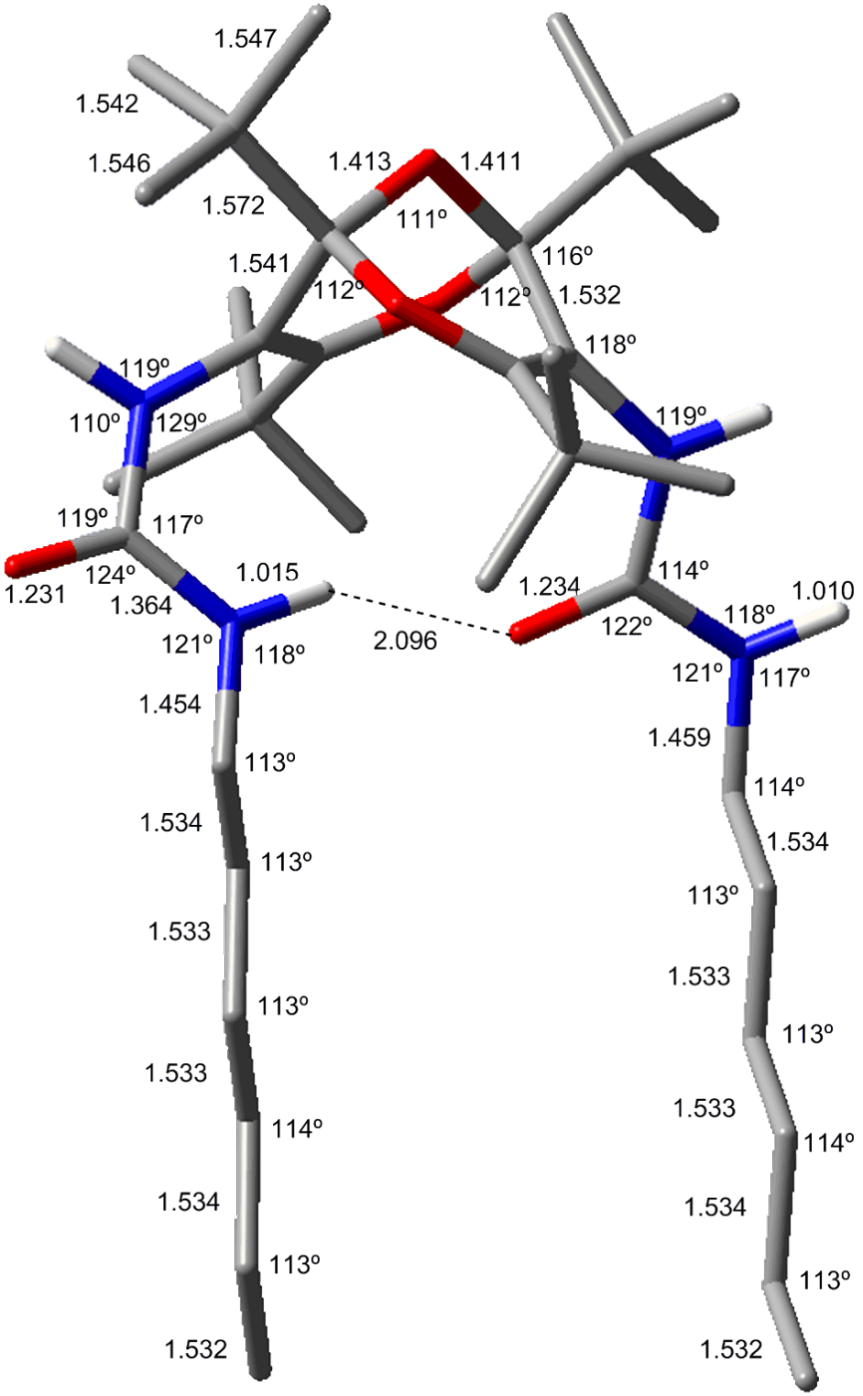
The lowest-energy calculated structure of **4** (B3LYP/6-31G**; for other conformers and full details see Supporting Information File 1). Bond lengths are given in Å and angles in degrees. The interchain 2 Å hydrogen bond is indicated by a dashed line.

**Figure 7:**
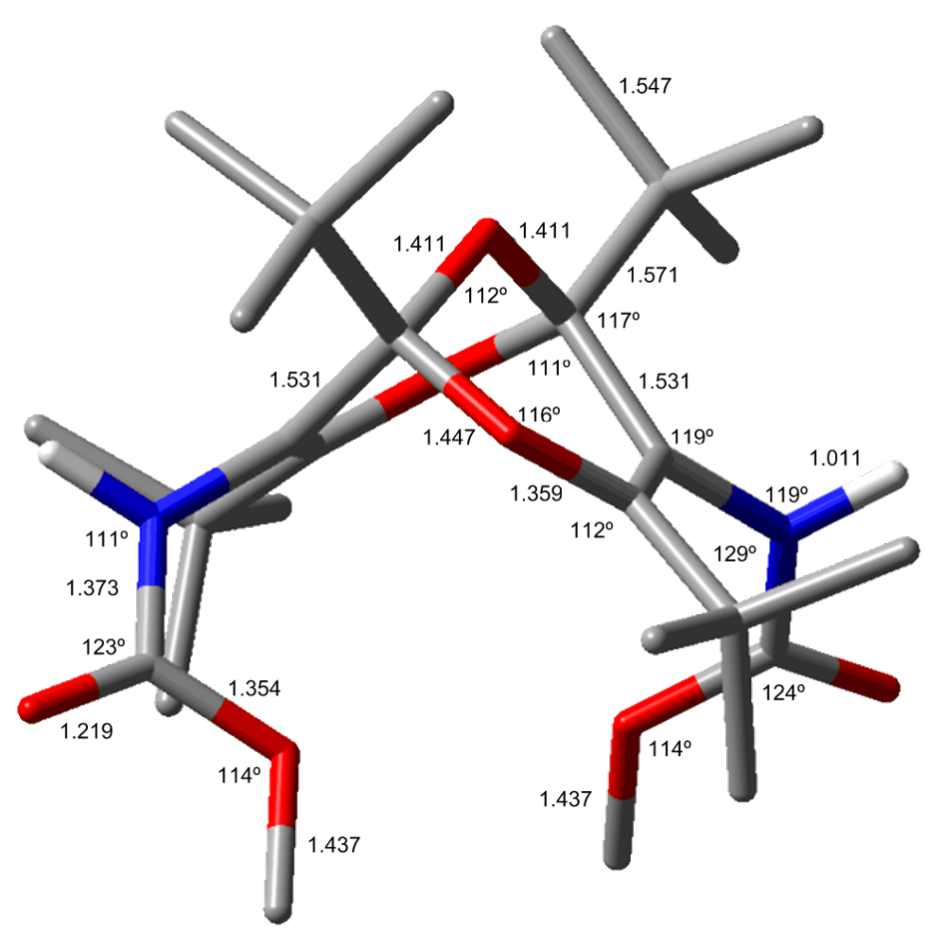
Calculated structure of **5** (B3LYP/6-31G**). Bond lengths are given in Å and angles in degrees. For another view and full details see Supporting Information File 1).

The structures of **4** and **5** were calculated at the B3LYP/6-31G** level, which reproduces the crystal structures very well, as demonstrated in Figure 6 and Figure 7 and in Figure S2 and Figure S3 (Supporting Information File 1).

While there are several possible conformations of **4** and **5** (see Figure S2 and Figure S3, Supporting Information File 1), the lowest-energy conformers shown in Figure 6 and Figure 7 are in very good agreement with the X-ray structures. The nearly parallel dangling chains in the lowest-energy conformer of **4** are particularly noteworthy (Figure 6). As in the crystal structure, the two chains are held in this orientation by 2 Å hydrogen bonds. The calculated structure of the diester **3** (Figure S4, Supporting Information File 1) is also in very good agreement with the previously determined crystal structure [3], but unlike the structures of **4** and **5** described above, the two ester moieties in compound **3** point away from each other with an angle of approximately 120° between them.

### Diamides

**Scheme 3:**
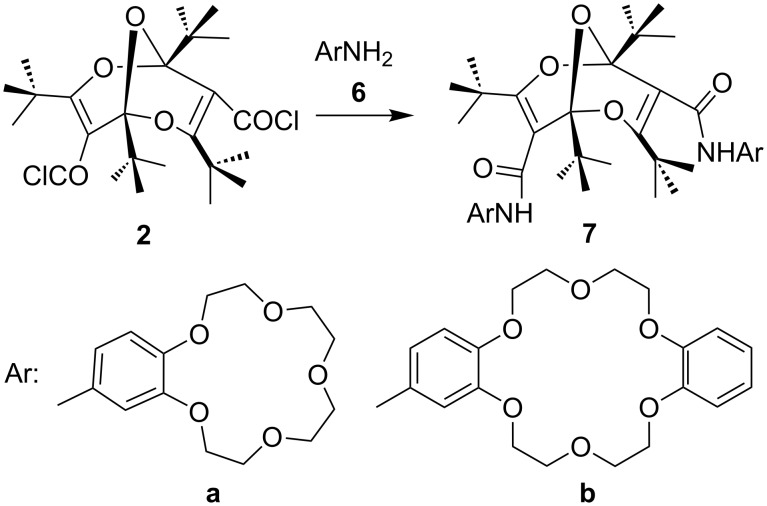
Synthesis of bis-crown ether diamides **7**.

**Figure 8:**
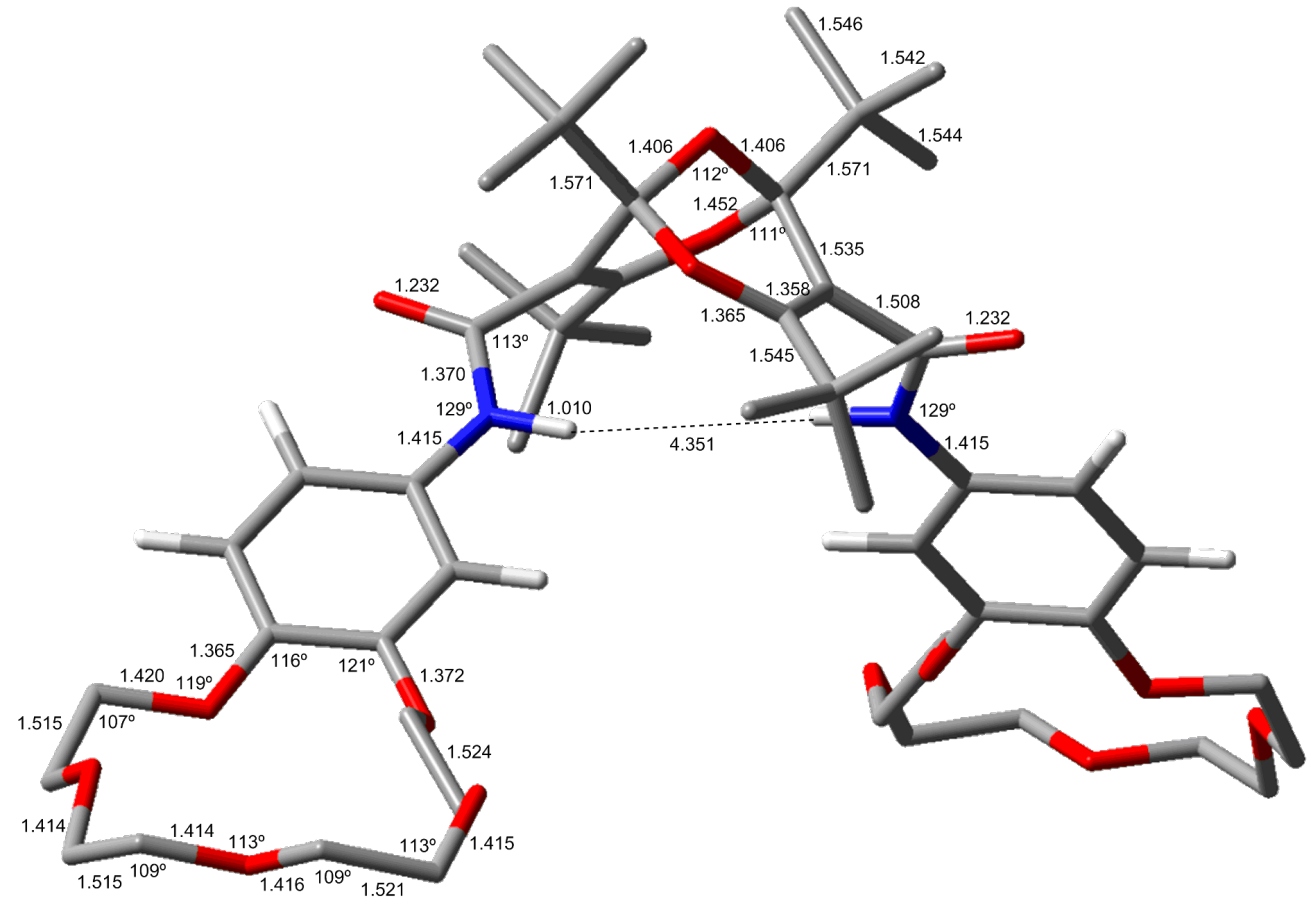
Calculated structure of **7a-a** (B3LYP/6-31G**). Bond lengths are given in Å and angles in degrees. For other conformers (**7a-b** and **7a-c**) and full details, see Supporting Information File 1.

**Figure 9:**
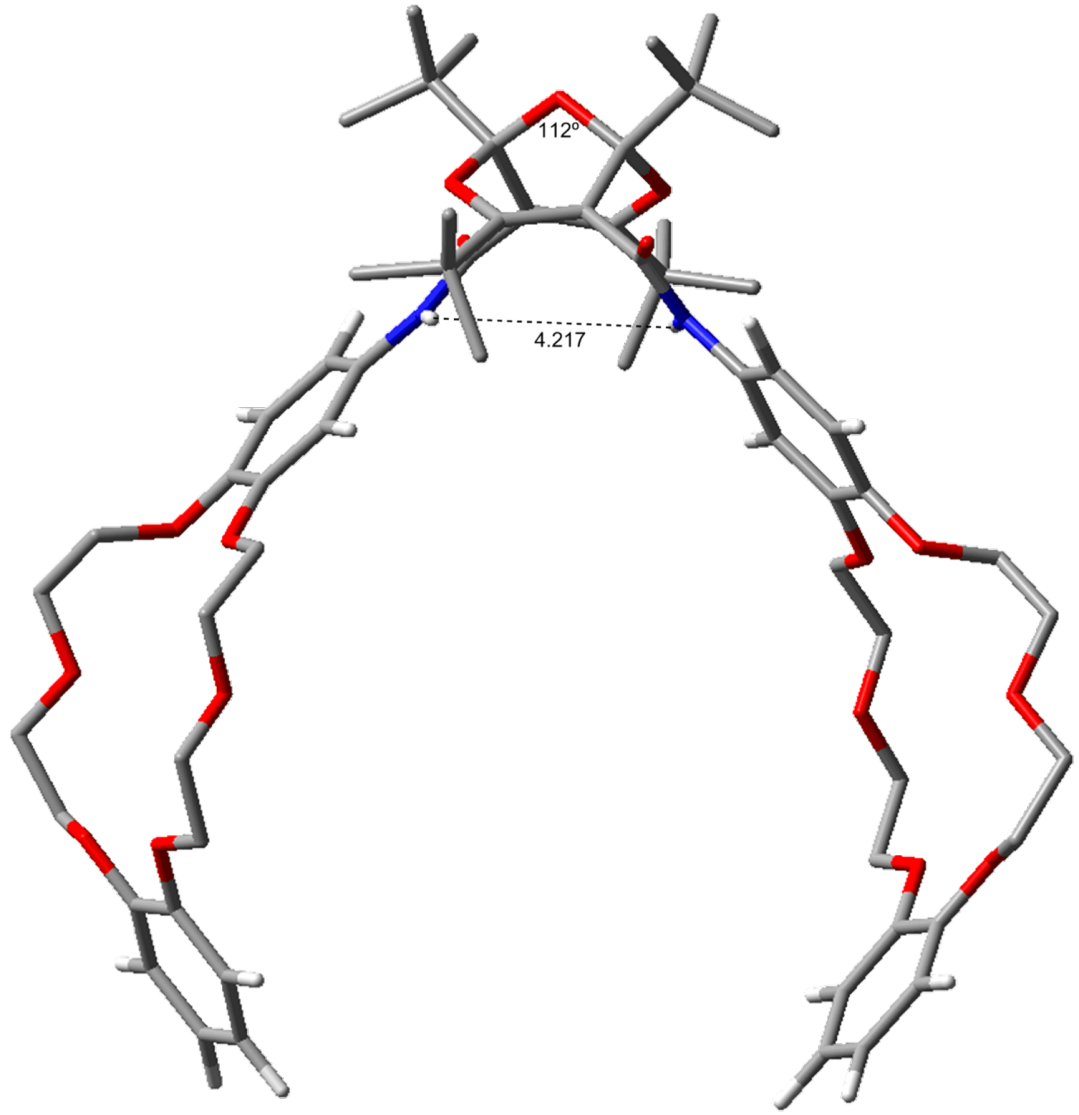
Calculated structure of **7b-a** (B3LYP/6-31G**). For other conformers (**7b-b** and **7b-c**) and full details, see Supporting Information File 1.

The *endo*,*endo*-structures of compounds **4** and **5** endow them with the character of molecular clefts or tweezers [4–10]. This suggests that other derivatives, such as the bis-crown ether diamides **7** (Scheme 3), would possess similar structures with parallel substituents. Accordingly, we investigated the structures of compound **7a** and **7b** computationally. Again, there are several possible conformers of each compound, but the lowest-energy conformers shown in Figure 8 and Figure 9 reveal the claw-like character of the dangling chains. This is particularly noteworthy in compound **7b**, where a cavity between the two crown-ether moieties is apparent (Figure 9). This suggests that this molecule might be able to form strong complexes with suitable metal ions.

### Cation extraction experiments

The *endo*,*endo* structures of bis-crown ether diamides **7** with parallel crown ether moieties would be expected to endow them with enhanced complexation abilities. The extraction of metal ions from aqueous to chloroform solution was examined for the picrates of Na^+^, K^+^, Ca^2+^ and Ce^3+^ by using a previously described procedure [11]. For comparison, the performance of the crown ether amines **6a** and **6b** was evaluated with the same extraction procedure. It is seen in Table 1 that there is a modest increase in the extraction ability of **7a** (6–13%) over **6a** (4–8%), but a more distinct improvement for **7b** (11–15%) compared to **6b** (3–9%).

**Table 1:** Extraction of metal ions (%) as picrates from H_2_O to CHCl_3_ mediated by bis-crown ether diamides **7a** and **7b** and the corresponding mono-crown ether amines **6a** and **6b**.

Ion/ ligand	Na^+^	K^+^	Ca^2+^	Ce^3+^

**7a**	11	6	9	13
**7b**	15	11	13	14
**6a**	4	4	6	8
**6b**	3	7	7	9

## Conclusion

X-ray structure determinations confirmed the *endo*,*endo* structures of the bisurea **4** and biscarbamate **5**, which in turn confirm the *endo*,*endo* structure of the diisocyanate **1**. Bis-crown ether diamide derivatives **7** also feature *endo*,*endo* structures as confirmed by DFT calculations at the B3LYP/6-31G** level. This endows them with claw-like molecular cleft or tweezer properties as manifested in enhanced abilities to extract selected alkali and alkaline earth metal and rare earth ions. The ready availability of diisocyanate **1** and diacid dichloride **2** [1–3,11] paves the way for the synthesis of many other types of compounds with hairpin turns and parallel pendant chains.

## Experimental

### General

Preparations of the di(hexylurea) **4**, the di(ethyl urethane) **5** and the diamides **7** were carried out as previously described [1,11–13].

### Crystallography

Crystal structures are represented in ORTEP [14]. Tables of crystal data and bond lengths and angles are presented in Supporting Information File 1. The full data can be obtained free of charge from the Cambridge Crystallographic Data Centre via http://www.ccdc.cam.ac.uk/data_request/cif. CCDC numbers 925965 and 925966.

### X-ray diffraction data for **4**

All the measurements were performed using graphite-monochromatized MoKα radiation at 100 K: C_36_H_66_N_4_O_5_, *M*_r_ 634.93, monoclinic, space group *P*2_1_/*c*, *a* = 21.0815(7) Å, *b* = 20.1551(6) Å, *c* = 17.9547(6) Å, β = 92.203(2)°, *V* = 7623.3(4) Å^3^, *Z* = 8, *d*_calc_ = 1.106 g cm^−3^, μ = 0.073 mm^−1^. A total of 34296 reflections were collected (Θ_max_ = 25.0°), from which 13358 were unique (*R*_int_ = 0.0344), with 8670 having *I* > 2σ(I). The structure was solved by direct methods (SHELXS-97) [15] and refined by full-matrix least-squares techniques against *F*^2^ (SHELXL-97) [15]. Three of the eight *tert*-butyl groups as well as two of the four hexyl groups in the two molecules of the asymmetric unit are disordered over two orientations. Their site occupation factors were refined to add to unity each and equivalent bonds were restrained to have the same lengths. The same anisotropic displacement parameters were used for equivalent atoms of the disordered groups. The other non-hydrogen atoms were refined with anisotropic displacement parameters without any constraints. The H atoms of the NH groups were refined with N–H distances of 0.88 Å, but without any further positional constraints, and with a common isotropic displacement parameter. The H atoms of the ordered CH_2_ groups were refined with their isotropic displacement parameters fixed to 1.2 times *U*_eq_ of the C atom they are bonded to and idealized geometry with approximately tetrahedral angles and C–H distances of 0.99 Å. The H atoms of the methyl groups were refined with their isotropic displacement parameters fixed to 1.3 times *U*_eq_ of the C atom they are bonded to and idealized geometry with tetrahedral angles, staggered conformation, and C–H distances of 0.98 Å. For 902 parameters final *R* indices of *R*1 = 0.0670 and w*R*^2^ = 0.1708 (GOF = 1.015) were obtained. The largest peak in a difference Fourier map was 0.400 eÅ^−3^.

### X-ray diffraction data for **5**

All the measurements were performed using graphite-monochromatized MoKα radiation at 100 K: C_26_H_44_N_2_O_7_, *M*_r_ 496.63, monoclinic, space group *C*2/*c*, *a* = 16.8698(6) Å, *b* = 10.4411(6) Å, *c* = 17.1497(7) Å, β = 113.903(3)°, *V* = 2761.7(2) Å^3^, *Z* = 4, *d*_calc_ = 1.194 g cm^−3^, μ = 0.086 mm^−1^. A total of 11810 reflections were collected (Θ_max_ = 27.5°), from which 3165 were unique (*R*_int_ = 0.0254), with 2622 having *I* > 2σ(I). The structure was solved by direct methods (SHELXS-97) [15] and refined by full-matrix least-squares techniques against *F*^2^ (SHELXL-97) [15]. The non-hydrogen atoms were refined with anisotropic displacement parameters without any constraints. The H atom bonded to N4 was refined without any positional constraints with an individual isotropic displacement parameter. The H atoms of the methyl groups were refined with common isotropic displacement parameters for the H atoms of the same group and idealized geometries with tetrahedral angles, enabling rotation around the X–C bond, and C–H distances of 0.98 Å. For 177 parameters final *R* indices of *R*1 = 0.0377 and w*R*^2^ = 0.1058 (GOF = 1.045) were obtained. The largest peak in a difference Fourier map was 0.408 eÅ^−3^.

### Extraction experiments

The extractions of ions from H_2_O to CHCl_3_ with the aid of bis-crown ether amides **7a** and **7c** (Table 1) were carried out as described previously [11,16]. Briefly, equimolar amounts of picrate and crown ether in H_2_O and CHCl_3_ respectively were shaken vigorously for 10 min. The extent of extraction into CHCl_3_ was measured by UV spectrophotometry at 354 nm.

## Supporting Information

File 1 Hydrogen bonding pattern in **5**, calculated structures of compounds **3**, **4**, **5**, **7a** and **7b**. X-ray structural data, bond lengths and bond angles for **4** and **5**, and preparative and computational details.
