# Supplementary material for: Molecular cleft or tweezer compounds derived from trioxabicyclo[3.3.1]nonadiene diisocyanate and diacid dichloride
Source: Beilstein J Org Chem. 2015 Jan 2;11:1–8. doi: 10.3762/bjoc.11.1 (PMC4311622; doi:10.3762/bjoc.11.1)
Supplement: File 1 — Hydrogen bonding pattern in 5, calculated structures of compounds 3, 4, 5, 7a and 7b. X-ray structural data, bond lengths and bond angles for 4 and 5, and preparative and computational details. [file Beilstein_J_Org_Chem-11-01-s001.pdf]

Supporting Information

for

Molecular cleft or tweezer compounds derived from trioxabicyclo[3.3.1]nonadiene diisocyanate and diacid dichloride

Gert Kollenz*[1], Ralf Smounig[1], Ferdinand Belaj[1], David Kvaskoff[2], and Curt Wentrup*[2]

Address: [1]Institute of Chemistry, Karl-Franzens University of Graz, Heinrichstrasse 28, A-8010 Graz, Austria and [2]School of Chemistry and Molecular Biosciences, The University of Queensland, Brisbane, Qld 4072, Australia

Email: Gert Kollenz* - gert.kollenz@uni-graz.at; Curt Wentrup* - wentrup@uq.edu.au

*Corresponding author

Hydrogen bonding pattern in **5**, calculated structures of compounds **3**, **4**, **5**, **7a** and **7b**. X-ray structural data, bond lengths and bond angles for **4** and **5**, and preparative and computational details.

Table of contents:

Figure S1. Plot of the hydrogen bonding scheme of **5** S2
Figure S2. Calculated structures of **4**........ S3
Figure S3. Calculated structure of **5** S4

Figure S4. Calculated structure of **3** S5
Figure S5. Calculated structures of **7a** S6
Figure S6. Calculated structure of **7b** S7
Preparations S8
X-ray crystal data, structures, bond lengths, bond angles, and
hydrogen bonds for **4** and **5** S8
Summary table of relative energies and harmonic frequencies,
calculated at the B3LYP/6-31G** level. S26
Cartesian coordinates and energies calculated at the B3LYP/6-31G** level. S26
References S60

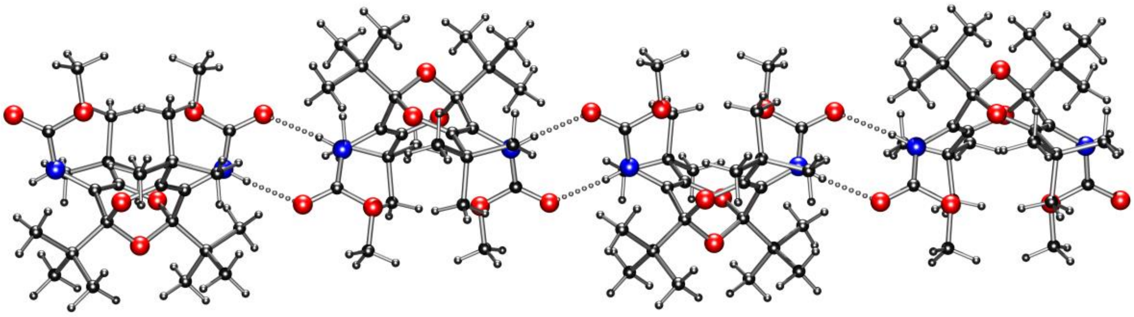

Figure S1: Plot of the hydrogen bonding scheme of **5.** The atoms are drawn with arbitrary radii, the hydrogen bonds are indicated by dotted lines.

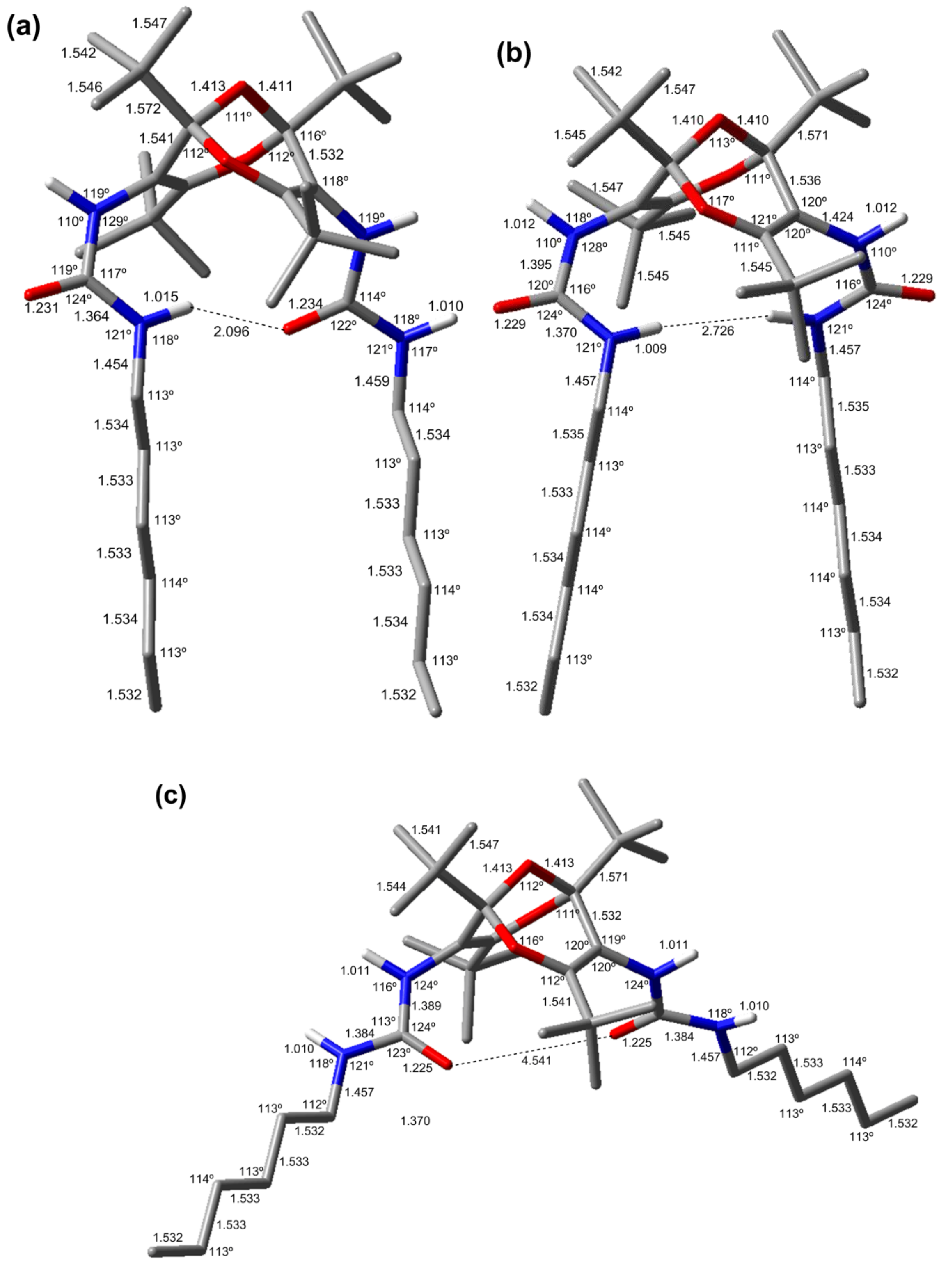

Figure S2: Calculated structures (B3LYP/6-31G**) of 1,3,5,7-tetra-*tert*-butyl-2,6,9-trioxabicyclo[3.3.1]nona-3,7-diene-4,8-diyl-bis(3-hexylurea) (compound **4**, three isomers **4a**, **4b** and **4c**), including bond lengths [Å] and bond angles [°]. Methyl hydrogens omitted for clarity.

Relative energies (see Table S7, p S26):**4a**: 0.0; **4b**: 3.9; **4c**: 5.5 kcal/mol.

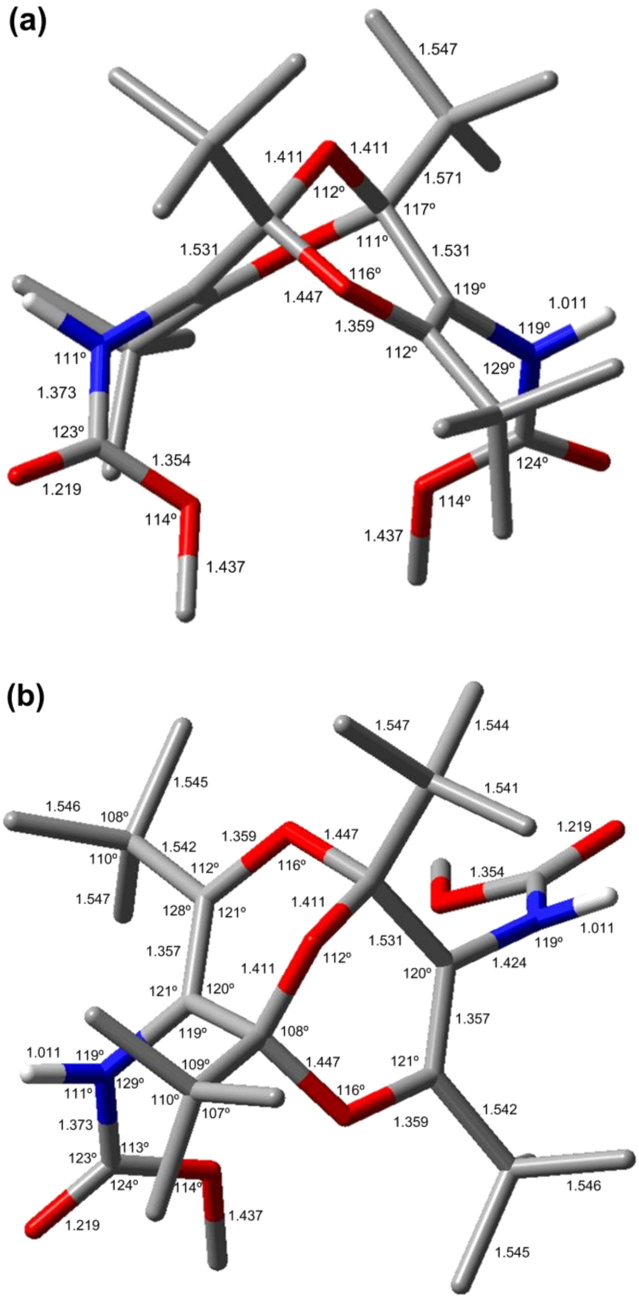

Figure S3: Two views of the calculated structure (B3LYP/6-31G**) of dimethyl(1,3,5,7-tetra-*tert*-butyl-2,6,9-trioxabi-cyclo[3.3.1]nona-3,7-diene-4,8-diyl)biscarbamate (compound **5**, single isomer), including bond lengths [Å] and bond angles [°]. Methyl hydrogens omitted for clarity.

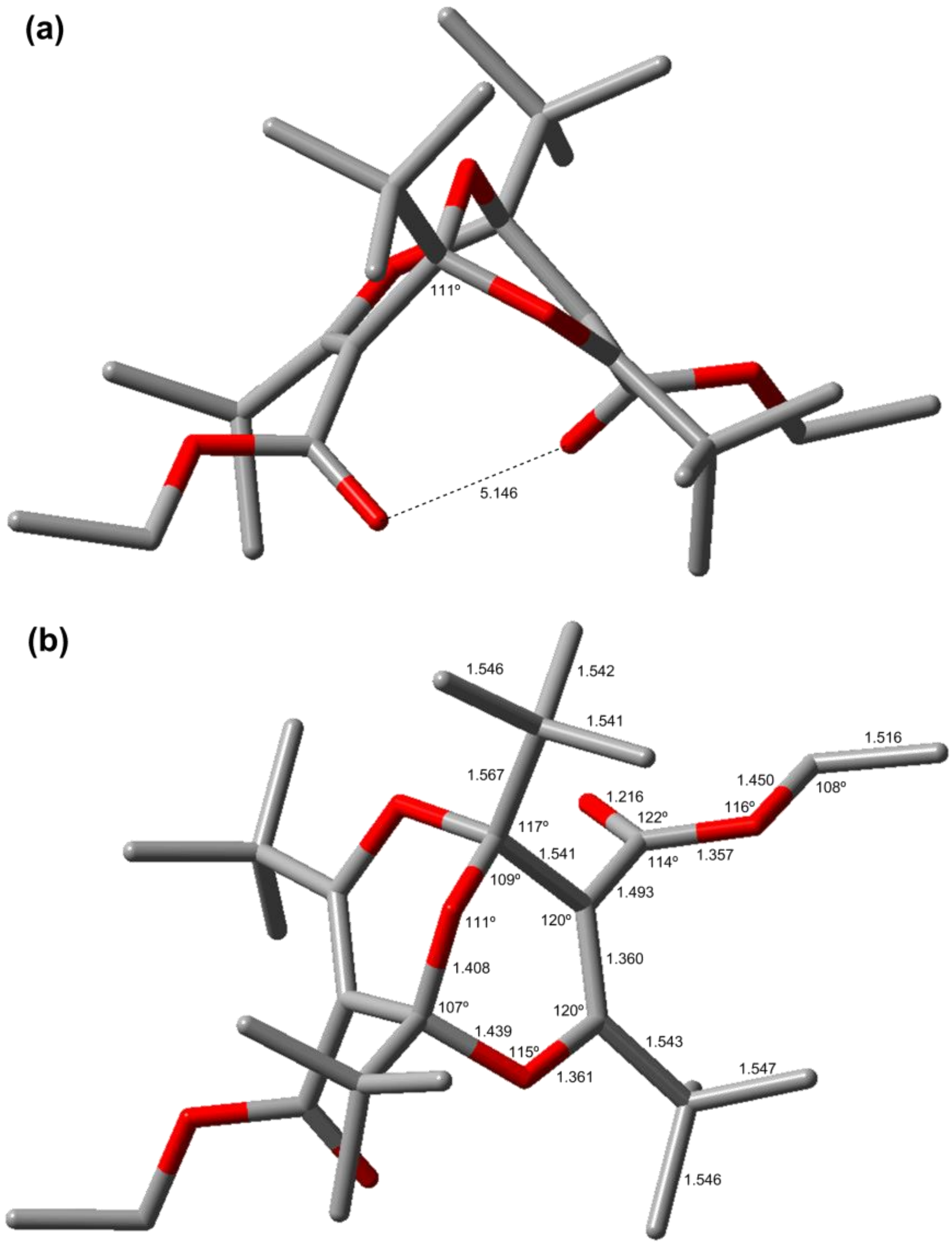

Figure S4: Two views of the calculated structure (C_2 symmetry, B3LYP/6-31G**) of diethyl ester **3**, including bond lengths [Å] and bond angles [°]. Methyl hydrogens omitted for clarity. The X-ray crystal structure of this compound is reported in reference [1].

(a)

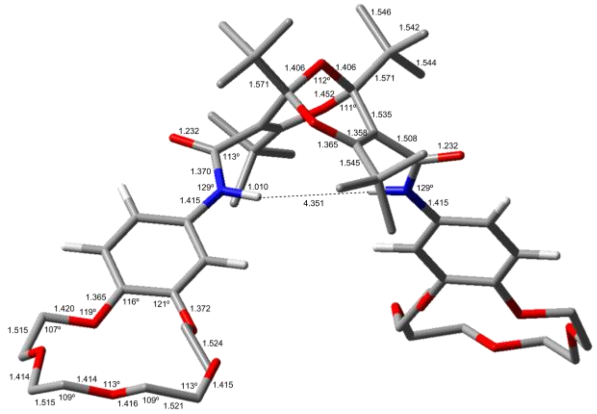

(b)

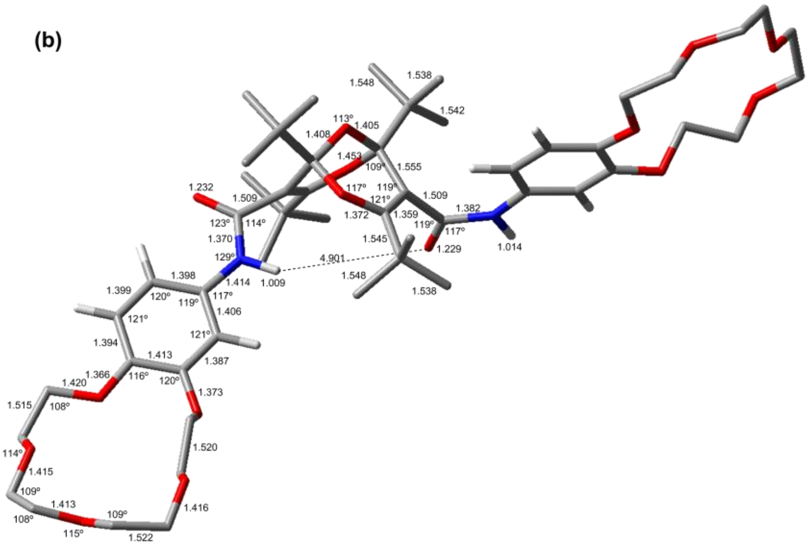

(c)

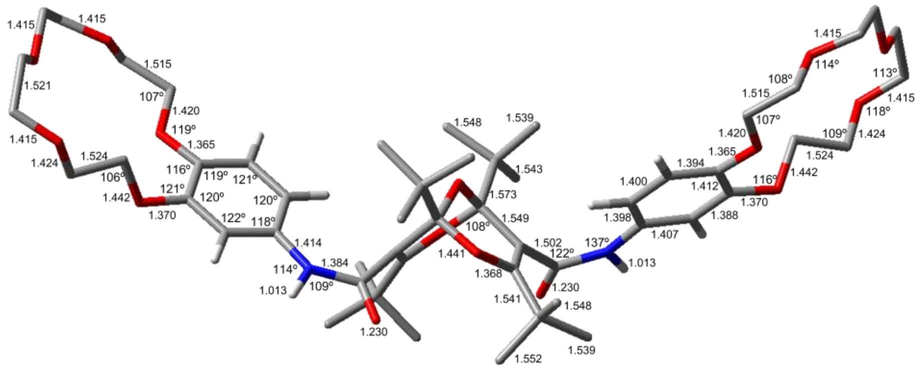

Figure S5: Calculated structures (B3LYP/6-31G**) of **7a** (3 isomers **7a-a**, **7a-b**, and **7a-c**), including bond lengths [Å] and bond angles [º]. Methyl and methylene hydrogens omitted for clarity. **Relative energies** (see Table S7, p S26): **7a-a**: 0.0; **7a-b**: 12.2; **7a-c**: 21.0 kcal/mol.

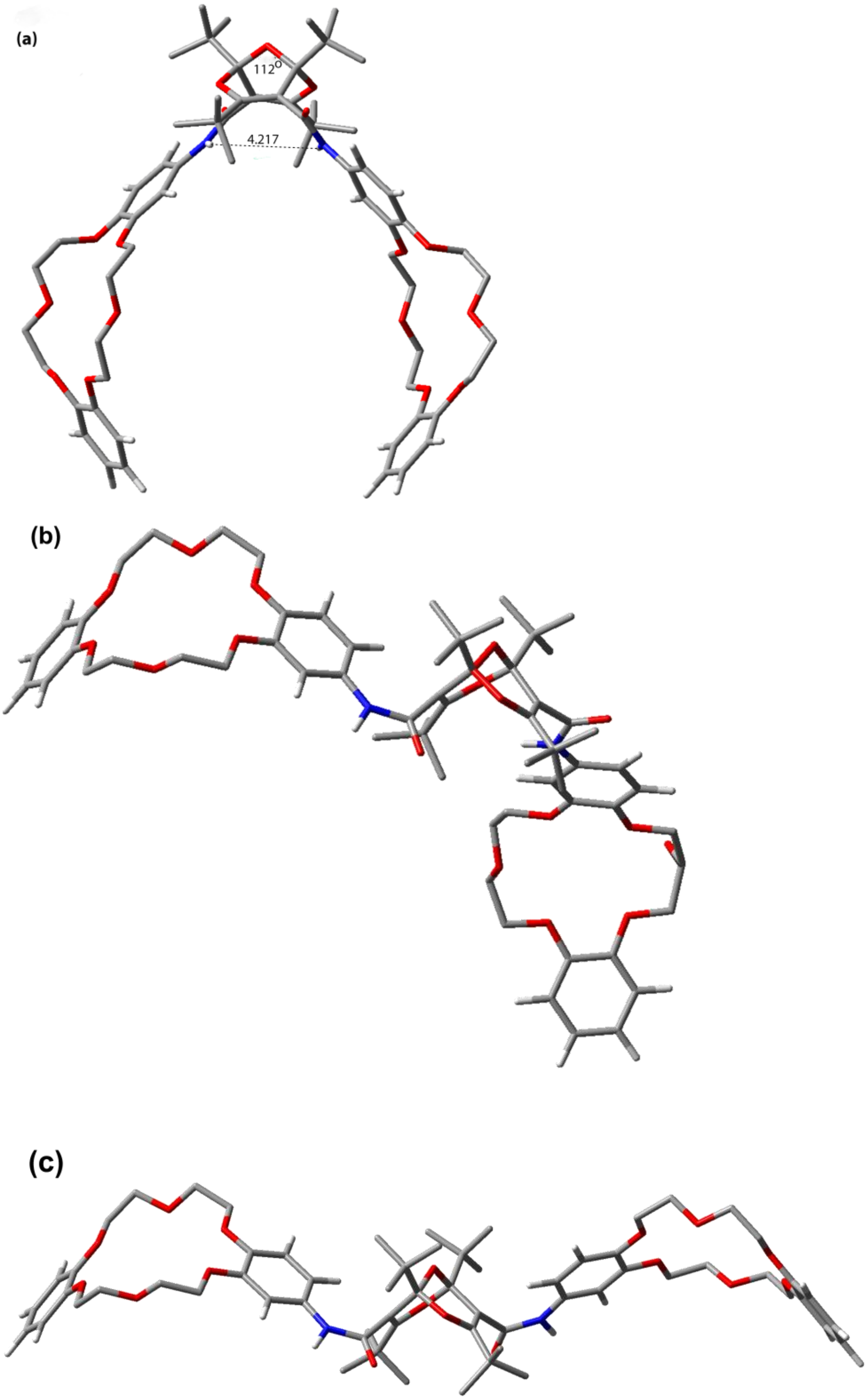

Figure S6: Calculated structures (B3LYP/6-31G**) of **7b** (3 isomers, **7b-a**, **7b-b**, and **7b-c**), including bond lengths [Å] and bond angles [°]. Methyl and methylene hydrogens omitted for clarity. **Relative energies** (see Table S7, p S26): **7b-a** 0.0, **7b-b** 12.3, **7b-c** 16.8 kcal/mol.

Preparations

Preparations of the di(hexylurea) **4**, the di(ethyl urethane) **5** and the diamides **7** were carried out as described in references [2-5].

(1,3,5,7-Tetra-*tert*-butyl-2,6,9-trioxabicyclo[3.3.1]nona-3,7-diene)-4,8-dicarboxylic acid-(benzo-15-crown-5)-4'-yl-amide (7a)

Yield : 325 mg (80%); mp 100° C; IR (KBr): 3430 (NH), 304-2870 (CH), 1675 (C=O), 1610 (C=C) cm^{-1}

^{1}H-NMR($CDCl_3$): δ = 1.15, 1.4 (2s, 2x18H, *t*-Bu), 3.7-4.2 (m, 32H), 6.82 (b, 4H, Ar-H), 7.10 (s, 2H, Ar-H, 7.38 (s, 2NH) ppm;

^{13}C-NMR ($CDCl_3$): δ = 24.8, 29.4 (4C(**C**$H_3)_3$), 38.1, 40.3 (4**C** $(CH_3)_3$), 69.0, 69.5, 69.7, 69.8, 70.5, 70.7, 71.1, 71.2, (OCH_2CH_2), 98.2 (C-1/C-5), 106.7 (C-4/C-8), 111.8, 115.0, 132,0, 146.1, 149.6 (Ar-C), 161.8 (C-3/C-7), 165.8 (CO) ppm.

Anal.Calcd. for $C_{52}H_{76}N_2O_{15}$ x H_2O : C, 63.27; H, 7.90; N, 2.84; found : C, 63.50; H, 7.95; N, 2.95.

(1,3,5,7-Tetra-*tert*-butyl-2,6,9-trioxabicyclo[3.3.1]nona-3,7-diene)-4,8-dicarboxylic acid-(dibenzo-18-crown-6)-4'-yl-amide (7b)

Yield: 410 mg (85%); mp 130°C; IR(KBr): 3420 (NH), 3000 – 2870 (CH), 1680 (C=O), 1610 (C=C) cm^{-1}

^{1}H-NMR ($CDCl_3$): δ = 1.15, 1.4 (2s, 2x18H, *t*-Bu), 4.01 – 4.15 (m, 32H), 6.80-6.95 (m, 14H, Ar-H), 7.3 (s, 2NH) ppm.

Anal.Calcd. for $C_{64}H_{84}N_2O_{17}$ x H_2O : C, 65.62; H, 7.40; N, 2.39; found: C, 65.51; H, 7.15; N, 2.40.

Table S1: Crystal data and structure refinement for **4**.

Crystal data		
Identification code		KRS91
Empirical formula		$C_{36}H_{66}N_4O_5$
Formula weight		634.93
Crystal description		plate, colourless
Crystal size		0.32 x 0.27 x 0.09mm
Crystal system, space group		monoclinic, P 21/c
Unit cell dimensions:	a	21.0815(7)Å
	b	20.1551(6)Å
	c	17.9547(6)Å
	β	92.203(2)°
Volume		7623.3(4)$Å^3$
Z		8
Calculated density		1.106Mg/m^3
F(000)		2800
Linear absorption coefficient μ		0.073mm^{-1}
Absorption correction		semi-empirical from equivalents
Max. and min. transmission		1.0000 and 0.7564
Unit cell determination		2.49° < Θ < 26.19°
		7734 reflections used at 100K
Data collection		
Temperature		100K
Diffractometer		Bruker APEX-II CCD
Radiation source		fine-focus sealed tube
Radiation and wavelength		MoK_α, 0.71073Å
Monochromator		graphite
Scan type		ϕ and ω scans
Θ range for data collection		2.18 to 25.00°

Index ranges	$-17 \le h \le 25$, $-23 \le k \le 23$, $-21 \le l \le 17$
Reflections collected / unique	34296 / 13358
Significant unique reflections	8670 with $I > 2\sigma(I)$
R(int), R(sigma)	0.0344, 0.0560
Completeness to Θ = 25.0°	99.5%
**Refinement**	
Refinement method	Full-matrix least-squares on F^2
Data / parameters / restraints	13358 / 902 / 37
Goodness-of-fit on F^2	1.015
Final R indices [$I > 2\sigma(I)$]	R1 = 0.0670, wR2 = 0.1498
R indices (all data)	R1 = 0.1112, wR2 = 0.1708
Extinction expression	none
Weighting scheme	$w = 1/[\sigma^2(F_o^2)+(aP)^2+bP]$ where $P = (F_o^2+2F_c^2)/3$
Weighting scheme parameters a, b	0.0672, 6.5922
Largest Δ/σ in last cycle	0.001
Largest difference peak and hole	0.400 and -0.341e/Å^3
Structure Solution Program	SHELXS-97 (Sheldrick, 2008)
Structure Refinement Program	SHELXL-97 (Sheldrick, 2008)

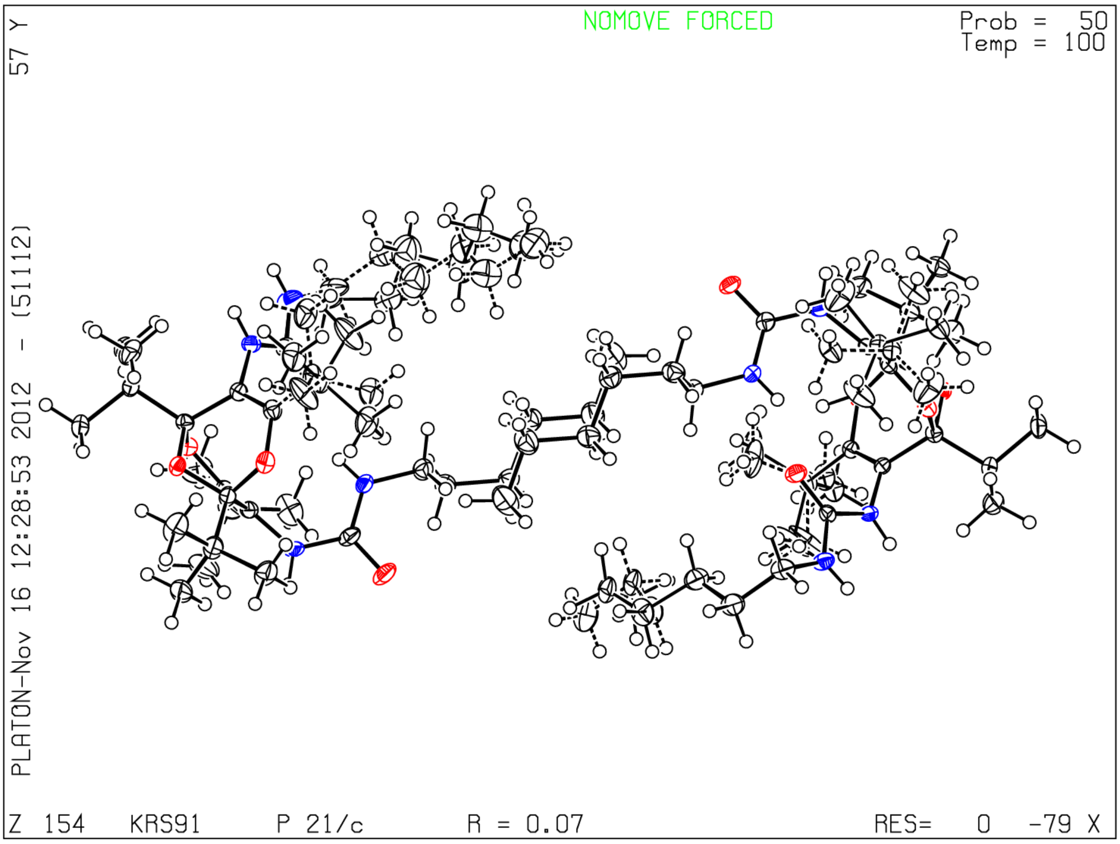

Table S2: Hydrogen bonds for **4** (**KRS91)** [Å, °].

D-H···A	d(D-H)	d(H···A)	d(D···A)	<(DHA)
N(32)-H(32)···O(40)	0.88	1.970(14)	2.763(3)	149(2)
N(41)-H(41)···O(30)[i)]	0.88	2.057(12)	2.876(3)	154(2)
N(42)-H(42)···O(30)[i)]	0.88	2.131(13)	2.935(3)	151(2)
N(82)-H(82)···O(90)	0.88	2.006(12)	2.823(3)	154(2)
N(91)-H(91)···O(80)[ii)]	0.88	2.043(13)	2.852(3)	152(2)
N(92)-H(92)···O(80)[ii)]	0.88	2.105(13)	2.914(3)	152(2)

Symmetry transformations used to generate equivalent atoms:

[i)] x, 3/2-y, z-1/2 [ii)] x, 1/2-y, z+1/2

Table S3:. Full list of bond lengths [Å] and angles [°] for **4 (KRS91)**.

C(1)-O(9)	1.414(3)	C(117)-H(117)	0.98
C(1)-O(2)	1.434(3)	C(117)-H(217)	0.98
C(1)-C(8)	1.520(3)	C(117)-H(317)	0.98
C(1)-C(11)	1.555(3)	C(118)-H(118)	0.98
O(2)-C(3)	1.373(3)	C(118)-H(218)	0.98
C(3)-C(4)	1.338(4)	C(118)-H(318)	0.98
C(3)-C(15)	1.533(4)	C(19)-C(21)	1.534(4)
C(3)-C(115)	1.537(10)	C(19)-C(20)	1.537(4)
C(4)-N(31)	1.429(3)	C(19)-C(22)	1.540(4)
C(4)-C(5)	1.519(3)	C(20)-H(201)	0.98
C(5)-O(9)	1.406(3)	C(20)-H(202)	0.98
C(5)-O(6)	1.437(3)	C(20)-H(203)	0.98
C(5)-C(19)	1.561(3)	C(21)-H(211)	0.98
O(6)-C(7)	1.372(3)	C(21)-H(212)	0.98
C(7)-C(8)	1.347(3)	C(21)-H(213)	0.98
C(7)-C(123)	1.531(7)	C(22)-H(221)	0.98
C(7)-C(23)	1.531(5)	C(22)-H(222)	0.98
C(8)-N(41)	1.417(3)	C(22)-H(223)	0.98
C(11)-C(14)	1.534(4)	C(23)-C(25)	1.574(13)
C(11)-C(13)	1.536(4)	C(23)-C(26)	1.575(13)
C(11)-C(12)	1.540(4)	C(23)-C(24)	1.575(13)
C(12)-H(121)	0.98	C(24)-H(241)	0.98
C(12)-H(122)	0.98	C(24)-H(242)	0.98
C(12)-H(123)	0.98	C(24)-H(243)	0.98
C(13)-H(131)	0.98	C(25)-H(251)	0.98
C(13)-H(132)	0.98	C(25)-H(252)	0.98
C(13)-H(133)	0.98	C(25)-H(253)	0.98
C(14)-H(141)	0.98	C(26)-H(261)	0.98
C(14)-H(142)	0.98	C(26)-H(262)	0.98
C(14)-H(143)	0.98	C(26)-H(263)	0.98
C(15)-C(17)	1.510(5)	C(123)-C(125)	1.41(3)
C(15)-C(18)	1.532(5)	C(123)-C(126)	1.42(3)
C(15)-C(16)	1.556(4)	C(123)-C(124)	1.47(3)
C(16)-H(161)	0.98	C(124)-H(124)	0.98
C(16)-H(162)	0.98	C(124)-H(224)	0.98
C(16)-H(163)	0.98	C(124)-H(324)	0.98
C(17)-H(171)	0.98	C(125)-H(125)	0.98
C(17)-H(172)	0.98	C(125)-H(225)	0.98
C(17)-H(173)	0.98	C(125)-H(325)	0.98
C(18)-H(181)	0.98	C(126)-H(126)	0.98
C(18)-H(182)	0.98	C(126)-H(226)	0.98
C(18)-H(183)	0.98	C(126)-H(326)	0.98
C(115)-C(117)	1.542(12)	N(31)-C(30)	1.363(3)
C(115)-C(118)	1.543(12)	N(31)-H(31)	0.88
C(115)-C(116)	1.545(12)	C(30)-O(30)	1.248(3)
C(116)-H(116)	0.98	C(30)-N(32)	1.333(3)
C(116)-H(216)	0.98	N(32)-C(31)	1.456(3)
C(116)-H(316)	0.98	N(32)-H(32)	0.88

C(31)-C(32)	1.525(4)
C(31)-H(311)	0.99
C(31)-H(312)	0.99
C(32)-C(33)	1.524(4)
C(32)-H(321)	0.99
C(32)-H(322)	0.99
C(33)-C(34)	1.513(4)
C(33)-H(331)	0.99
C(33)-H(332)	0.99
C(34)-C(35)	1.527(4)
C(34)-H(341)	0.99
C(34)-H(342)	0.99
C(35)-C(36)	1.505(4)
C(35)-H(351)	0.99
C(35)-H(352)	0.99
C(36)-H(361)	0.98
C(36)-H(362)	0.98
C(36)-H(363)	0.98
N(41)-C(40)	1.367(3)
N(41)-H(41)	0.88
C(40)-O(40)	1.240(3)
C(40)-N(42)	1.350(3)
N(42)-C(41)	1.449(3)
N(42)-H(42)	0.88
C(41)-C(42)	1.523(4)
C(41)-H(411)	0.99
C(41)-H(412)	0.99
C(42)-C(43)	1.516(4)
C(42)-H(421)	0.99
C(42)-H(422)	0.99
C(43)-C(144)	1.527(10)
C(43)-C(44)	1.534(4)
C(43)-H(431)	0.99
C(43)-H(432)	0.99
C(44)-C(45)	1.544(5)
C(44)-H(441)	0.99
C(44)-H(442)	0.99
C(45)-C(46)	1.503(5)
C(45)-H(451)	0.99
C(45)-H(452)	0.99
C(46)-H(461)	0.98
C(46)-H(462)	0.98
C(46)-H(463)	0.98
C(144)-C(145)	1.541(11)
C(144)-H(144)	0.99
C(144)-H(244)	0.99
C(145)-C(146)	1.506(10)
C(145)-H(145)	0.99
C(145)-H(245)	0.99
C(146)-H(146)	0.98
C(146)-H(246)	0.98
C(146)-H(346)	0.98
C(51)-O(59)	1.410(3)
C(51)-O(52)	1.433(3)
C(51)-C(58)	1.519(3)
C(51)-C(61)	1.558(3)
O(52)-C(53)	1.364(3)
C(53)-C(54)	1.336(4)
C(53)-C(65)	1.529(4)
C(54)-N(81)	1.429(3)
C(54)-C(55)	1.524(4)
C(55)-O(59)	1.410(3)
C(55)-O(56)	1.442(3)
C(55)-C(69)	1.561(4)
O(56)-C(57)	1.367(3)
C(57)-C(58)	1.344(3)
C(57)-C(73)	1.536(5)
C(57)-C(173)	1.537(8)
C(58)-N(91)	1.417(3)
C(61)-C(64)	1.528(4)
C(61)-C(62)	1.531(4)
C(61)-C(63)	1.538(4)
C(62)-H(621)	0.98
C(62)-H(622)	0.98
C(62)-H(623)	0.98
C(63)-H(631)	0.98
C(63)-H(632)	0.98
C(63)-H(633)	0.98
C(64)-H(641)	0.98
C(64)-H(642)	0.98
C(64)-H(643)	0.98
C(65)-C(67)	1.515(4)
C(65)-C(66)	1.540(4)
C(65)-C(68)	1.541(4)
C(66)-H(661)	0.98
C(66)-H(662)	0.98
C(66)-H(663)	0.98
C(67)-H(671)	0.98
C(67)-H(672)	0.98
C(67)-H(673)	0.98
C(68)-H(681)	0.98
C(68)-H(682)	0.98
C(68)-H(683)	0.98
C(69)-C(72)	1.530(4)
C(69)-C(70)	1.539(4)
C(69)-C(71)	1.541(4)
C(70)-H(701)	0.98
C(70)-H(702)	0.98
C(70)-H(703)	0.98
C(71)-H(711)	0.98

C(71)-H(712)	0.98
C(71)-H(713)	0.98
C(72)-H(721)	0.98
C(72)-H(722)	0.98
C(72)-H(723)	0.98
C(73)-C(76)	1.524(6)
C(73)-C(74)	1.531(5)
C(73)-C(75)	1.535(5)
C(74)-H(741)	0.98
C(74)-H(742)	0.98
C(74)-H(743)	0.98
C(75)-H(751)	0.98
C(75)-H(752)	0.98
C(75)-H(753)	0.98
C(76)-H(761)	0.98
C(76)-H(762)	0.98
C(76)-H(763)	0.98
C(173)-C(175)	1.513(8)
C(173)-C(174)	1.517(8)
C(173)-C(176)	1.518(8)
C(174)-H(174)	0.98
C(174)-H(274)	0.98
C(174)-H(374)	0.98
C(175)-H(175)	0.98
C(175)-H(275)	0.98
C(175)-H(375)	0.98
C(176)-H(176)	0.98
C(176)-H(276)	0.98
C(176)-H(376)	0.98
N(81)-C(80)	1.371(3)
N(81)-H(81)	0.88
C(80)-O(80)	1.248(3)
C(80)-N(82)	1.335(3)
N(82)-C(81)	1.455(3)
N(82)-H(82)	0.88
C(81)-C(82)	1.521(4)
C(81)-H(811)	0.99
C(81)-H(812)	0.99
C(82)-C(83)	1.526(4)
C(82)-H(821)	0.99
C(82)-H(822)	0.99
C(83)-C(84)	1.519(4)
C(83)-H(831)	0.99
C(83)-H(832)	0.99
C(84)-C(85)	1.526(4)
C(84)-H(841)	0.99
C(84)-H(842)	0.99
C(85)-C(86)	1.511(4)
C(85)-H(851)	0.99
C(85)-H(852)	0.99
C(86)-H(861)	0.98
C(86)-H(862)	0.98
C(86)-H(863)	0.98
N(91)-C(90)	1.364(3)
N(91)-H(91)	0.88
C(90)-O(90)	1.242(3)
C(90)-N(92)	1.348(3)
N(92)-C(191)	1.458(6)
N(92)-C(91)	1.463(6)
N(92)-H(92)	0.88
C(91)-C(92)	1.525(11)
C(91)-H(911)	0.99
C(91)-H(912)	0.99
C(92)-C(93)	1.486(8)
C(92)-H(921)	0.99
C(92)-H(922)	0.99
C(93)-C(94)	1.539(9)
C(93)-H(931)	0.99
C(93)-H(932)	0.99
C(94)-C(95)	1.479(8)
C(94)-H(941)	0.99
C(94)-H(942)	0.99
C(95)-C(96)	1.48(2)
C(95)-H(951)	0.99
C(95)-H(952)	0.99
C(96)-H(961)	0.98
C(96)-H(962)	0.98
C(96)-H(963)	0.98
C(191)-C(192)	1.523(10)
C(191)-H(191)	0.99
C(191)-H(291)	0.99
C(192)-C(193)	1.485(8)
C(192)-H(192)	0.99
C(192)-H(292)	0.99
C(193)-C(194)	1.534(9)
C(193)-H(193)	0.99
C(193)-H(293)	0.99
C(194)-C(195)	1.491(10)
C(194)-H(194)	0.99
C(194)-H(294)	0.99
C(195)-C(196)	1.48(2)
C(195)-H(195)	0.99
C(195)-H(295)	0.99
C(196)-H(196)	0.98
C(196)-H(296)	0.98
C(196)-H(396)	0.98

O(9)-C(1)-O(2)	107.67(18)
O(9)-C(1)-C(8)	108.51(19)
O(2)-C(1)-C(8)	111.41(19)

O(9)-C(1)-C(11)	105.90(19)
O(2)-C(1)-C(11)	106.57(19)
C(8)-C(1)-C(11)	116.3(2)
C(3)-O(2)-C(1)	114.33(18)
C(4)-C(3)-O(2)	119.4(2)
C(4)-C(3)-C(15)	130.4(2)
O(2)-C(3)-C(15)	110.1(2)
C(4)-C(3)-C(115)	141.9(6)
O(2)-C(3)-C(115)	98.1(6)
C(3)-C(4)-N(31)	121.9(2)
C(3)-C(4)-C(5)	121.5(2)
N(31)-C(4)-C(5)	116.5(2)
O(9)-C(5)-O(6)	107.49(18)
O(9)-C(5)-C(4)	108.48(19)
O(6)-C(5)-C(4)	112.11(19)
O(9)-C(5)-C(19)	105.98(19)
O(6)-C(5)-C(19)	106.48(19)
C(4)-C(5)-C(19)	115.8(2)
C(7)-O(6)-C(5)	113.78(18)
C(8)-C(7)-O(6)	119.9(2)
C(8)-C(7)-C(123)	131.5(5)
O(6)-C(7)-C(123)	108.4(5)
C(8)-C(7)-C(23)	130.1(3)
O(6)-C(7)-C(23)	109.9(3)
C(7)-C(8)-N(41)	122.4(2)
C(7)-C(8)-C(1)	121.0(2)
N(41)-C(8)-C(1)	116.5(2)
C(5)-O(9)-C(1)	110.19(18)
C(14)-C(11)-C(13)	108.8(2)
C(14)-C(11)-C(12)	107.3(2)
C(13)-C(11)-C(12)	110.2(2)
C(14)-C(11)-C(1)	108.7(2)
C(13)-C(11)-C(1)	109.3(2)
C(12)-C(11)-C(1)	112.4(2)
C(11)-C(12)-H(121)	109.5
C(11)-C(12)-H(122)	109.5
H(121)-C(12)-H(122)	109.5
C(11)-C(12)-H(123)	109.5
H(121)-C(12)-H(123)	109.5
H(122)-C(12)-H(123)	109.5
C(11)-C(13)-H(131)	109.5
C(11)-C(13)-H(132)	109.5
H(131)-C(13)-H(132)	109.5
C(11)-C(13)-H(133)	109.5
H(131)-C(13)-H(133)	109.5
H(132)-C(13)-H(133)	109.5
C(11)-C(14)-H(141)	109.5
C(11)-C(14)-H(142)	109.5
H(141)-C(14)-H(142)	109.5
C(11)-C(14)-H(143)	109.5

H(141)-C(14)-H(143)	109.5
H(142)-C(14)-H(143)	109.5
C(17)-C(15)-C(18)	109.5(3)
C(17)-C(15)-C(3)	116.7(3)
C(18)-C(15)-C(3)	109.3(3)
C(17)-C(15)-C(16)	108.1(3)
C(18)-C(15)-C(16)	107.9(3)
C(3)-C(15)-C(16)	105.0(2)
C(15)-C(16)-H(161)	109.5
C(15)-C(16)-H(162)	109.5
H(161)-C(16)-H(162)	109.5
C(15)-C(16)-H(163)	109.5
H(161)-C(16)-H(163)	109.5
H(162)-C(16)-H(163)	109.5
C(15)-C(17)-H(171)	109.5
C(15)-C(17)-H(172)	109.5
H(171)-C(17)-H(172)	109.5
C(15)-C(17)-H(173)	109.5
H(171)-C(17)-H(173)	109.5
H(172)-C(17)-H(173)	109.5
C(15)-C(18)-H(181)	109.5
C(15)-C(18)-H(182)	109.5
H(181)-C(18)-H(182)	109.5
C(15)-C(18)-H(183)	109.5
H(181)-C(18)-H(183)	109.5
H(182)-C(18)-H(183)	109.5
C(3)-C(115)-C(117)	117.4(11)
C(3)-C(115)-C(118)	105.5(10)
C(117)-C(115)-C(118)	108.3(14)
C(3)-C(115)-C(116)	105.7(10)
C(117)-C(115)-C(116)	111.5(13)
C(118)-C(115)-C(116)	108.0(13)
C(115)-C(116)-H(116)	109.5
C(115)-C(116)-H(216)	109.5
H(116)-C(116)-H(216)	109.5
C(115)-C(116)-H(316)	109.5
H(116)-C(116)-H(316)	109.5
H(216)-C(116)-H(316)	109.5
C(115)-C(117)-H(117)	109.5
C(115)-C(117)-H(217)	109.5
H(117)-C(117)-H(217)	109.5
C(115)-C(117)-H(317)	109.5
H(117)-C(117)-H(317)	109.5
H(217)-C(117)-H(317)	109.5
C(115)-C(118)-H(118)	109.5
C(115)-C(118)-H(218)	109.5
H(118)-C(118)-H(218)	109.5
C(115)-C(118)-H(318)	109.5
H(118)-C(118)-H(318)	109.5
H(218)-C(118)-H(318)	109.5

C(21)-C(19)-C(20)	111.0(2)
C(21)-C(19)-C(22)	108.6(2)
C(20)-C(19)-C(22)	107.4(2)
C(21)-C(19)-C(5)	108.9(2)
C(20)-C(19)-C(5)	112.5(2)
C(22)-C(19)-C(5)	108.3(2)
C(19)-C(20)-H(201)	109.5
C(19)-C(20)-H(202)	109.5
H(201)-C(20)-H(202)	109.5
C(19)-C(20)-H(203)	109.5
H(201)-C(20)-H(203)	109.5
H(202)-C(20)-H(203)	109.5
C(19)-C(21)-H(211)	109.5
C(19)-C(21)-H(212)	109.5
H(211)-C(21)-H(212)	109.5
C(19)-C(21)-H(213)	109.5
H(211)-C(21)-H(213)	109.5
H(212)-C(21)-H(213)	109.5
C(19)-C(22)-H(221)	109.5
C(19)-C(22)-H(222)	109.5
H(221)-C(22)-H(222)	109.5
C(19)-C(22)-H(223)	109.5
H(221)-C(22)-H(223)	109.5
H(222)-C(22)-H(223)	109.5
C(7)-C(23)-C(25)	106.2(8)
C(7)-C(23)-C(26)	110.4(10)
C(25)-C(23)-C(26)	108.0(9)
C(7)-C(23)-C(24)	115.3(8)
C(25)-C(23)-C(24)	108.2(8)
C(26)-C(23)-C(24)	108.5(11)
C(23)-C(24)-H(241)	109.5
C(23)-C(24)-H(242)	109.5
H(241)-C(24)-H(242)	109.5
C(23)-C(24)-H(243)	109.5
H(241)-C(24)-H(243)	109.5
H(242)-C(24)-H(243)	109.5
C(23)-C(25)-H(251)	109.5
C(23)-C(25)-H(252)	109.5
H(251)-C(25)-H(252)	109.5
C(23)-C(25)-H(253)	109.5
H(251)-C(25)-H(253)	109.5
H(252)-C(25)-H(253)	109.5
C(23)-C(26)-H(261)	109.5
C(23)-C(26)-H(262)	109.5
H(261)-C(26)-H(262)	109.5
C(23)-C(26)-H(263)	109.5
H(261)-C(26)-H(263)	109.5
H(262)-C(26)-H(263)	109.5
C(125)-C(123)-C(126)	112(2)
C(125)-C(123)-C(124)	104.4(17)
C(126)-C(123)-C(124)	110(2)
C(125)-C(123)-C(7)	108.0(17)
C(126)-C(123)-C(7)	111(2)
C(124)-C(123)-C(7)	111.4(17)
C(123)-C(124)-H(124)	109.5
C(123)-C(124)-H(224)	109.5
H(124)-C(124)-H(224)	109.5
C(123)-C(124)-H(324)	109.5
H(124)-C(124)-H(324)	109.5
H(224)-C(124)-H(324)	109.5
C(123)-C(125)-H(125)	109.5
C(123)-C(125)-H(225)	109.5
H(125)-C(125)-H(225)	109.5
C(123)-C(125)-H(325)	109.5
H(125)-C(125)-H(325)	109.5
H(225)-C(125)-H(325)	109.5
C(123)-C(126)-H(126)	109.5
C(123)-C(126)-H(226)	109.5
H(126)-C(126)-H(226)	109.5
C(123)-C(126)-H(326)	109.5
H(126)-C(126)-H(326)	109.5
H(226)-C(126)-H(326)	109.5
C(30)-N(31)-C(4)	127.5(2)
C(30)-N(31)-H(31)	111.5(17)
C(4)-N(31)-H(31)	120.8(17)
O(30)-C(30)-N(32)	122.8(2)
O(30)-C(30)-N(31)	119.3(2)
N(32)-C(30)-N(31)	117.9(2)
C(30)-N(32)-C(31)	120.6(2)
C(30)-N(32)-H(32)	123.0(17)
C(31)-N(32)-H(32)	116.3(18)
N(32)-C(31)-C(32)	112.9(2)
N(32)-C(31)-H(311)	109.0
C(32)-C(31)-H(311)	109.0
N(32)-C(31)-H(312)	109.0
C(32)-C(31)-H(312)	109.0
H(311)-C(31)-H(312)	107.8
C(33)-C(32)-C(31)	113.3(2)
C(33)-C(32)-H(321)	108.9
C(31)-C(32)-H(321)	108.9
C(33)-C(32)-H(322)	108.9
C(31)-C(32)-H(322)	108.9
H(321)-C(32)-H(322)	107.7
C(34)-C(33)-C(32)	114.6(2)
C(34)-C(33)-H(331)	108.6
C(32)-C(33)-H(331)	108.6
C(34)-C(33)-H(332)	108.6
C(32)-C(33)-H(332)	108.6
H(331)-C(33)-H(332)	107.6
C(33)-C(34)-C(35)	113.8(2)

C(33)-C(34)-H(341)	108.8
C(35)-C(34)-H(341)	108.8
C(33)-C(34)-H(342)	108.8
C(35)-C(34)-H(342)	108.8
H(341)-C(34)-H(342)	107.7
C(36)-C(35)-C(34)	113.2(3)
C(36)-C(35)-H(351)	108.9
C(34)-C(35)-H(351)	108.9
C(36)-C(35)-H(352)	108.9
C(34)-C(35)-H(352)	108.9
H(351)-C(35)-H(352)	107.8
C(35)-C(36)-H(361)	109.5
C(35)-C(36)-H(362)	109.5
H(361)-C(36)-H(362)	109.5
C(35)-C(36)-H(363)	109.5
H(361)-C(36)-H(363)	109.5
H(362)-C(36)-H(363)	109.5
C(40)-N(41)-C(8)	122.5(2)
C(40)-N(41)-H(41)	118.5(18)
C(8)-N(41)-H(41)	118.9(18)
O(40)-C(40)-N(42)	122.6(2)
O(40)-C(40)-N(41)	123.0(2)
N(42)-C(40)-N(41)	114.4(2)
C(40)-N(42)-C(41)	122.8(2)
C(40)-N(42)-H(42)	118.1(18)
C(41)-N(42)-H(42)	119.0(18)
N(42)-C(41)-C(42)	114.4(2)
N(42)-C(41)-H(411)	108.7
C(42)-C(41)-H(411)	108.7
N(42)-C(41)-H(412)	108.7
C(42)-C(41)-H(412)	108.7
H(411)-C(41)-H(412)	107.6
C(43)-C(42)-C(41)	114.3(2)
C(43)-C(42)-H(421)	108.7
C(41)-C(42)-H(421)	108.7
C(43)-C(42)-H(422)	108.7
C(41)-C(42)-H(422)	108.7
H(421)-C(42)-H(422)	107.6
C(42)-C(43)-C(144)	124.7(8)
C(42)-C(43)-C(44)	112.5(3)
C(42)-C(43)-H(431)	109.1
C(144)-C(43)-H(431)	86.9
C(44)-C(43)-H(431)	109.1
C(42)-C(43)-H(432)	109.1
C(144)-C(43)-H(432)	115.6
C(44)-C(43)-H(432)	109.1
H(431)-C(43)-H(432)	107.8
C(43)-C(44)-C(45)	113.5(3)
C(43)-C(44)-H(441)	108.9
C(45)-C(44)-H(441)	108.9
C(43)-C(44)-H(442)	108.9
C(45)-C(44)-H(442)	108.9
H(441)-C(44)-H(442)	107.7
C(46)-C(45)-C(44)	115.0(3)
C(46)-C(45)-H(451)	108.5
C(44)-C(45)-H(451)	108.5
C(46)-C(45)-H(452)	108.5
C(44)-C(45)-H(452)	108.5
H(451)-C(45)-H(452)	107.5
C(45)-C(46)-H(461)	109.5
C(45)-C(46)-H(462)	109.5
H(461)-C(46)-H(462)	109.5
C(45)-C(46)-H(463)	109.5
H(461)-C(46)-H(463)	109.5
H(462)-C(46)-H(463)	109.5
C(43)-C(144)-C(145)	115.3(11)
C(43)-C(144)-H(144)	108.5
C(145)-C(144)-H(144)	108.5
C(43)-C(144)-H(244)	108.5
C(145)-C(144)-H(244)	108.5
H(144)-C(144)-H(244)	107.5
C(146)-C(145)-C(144)	110.6(13)
C(146)-C(145)-H(145)	109.5
C(144)-C(145)-H(145)	109.5
C(146)-C(145)-H(245)	109.5
C(144)-C(145)-H(245)	109.5
H(145)-C(145)-H(245)	108.1
C(145)-C(146)-H(146)	109.5
C(145)-C(146)-H(246)	109.5
H(146)-C(146)-H(246)	109.5
C(145)-C(146)-H(346)	109.5
H(146)-C(146)-H(346)	109.5
H(246)-C(146)-H(346)	109.5
O(59)-C(51)-O(52)	108.06(18)
O(59)-C(51)-C(58)	108.70(19)
O(52)-C(51)-C(58)	111.02(19)
O(59)-C(51)-C(61)	106.21(19)
O(52)-C(51)-C(61)	106.79(19)
C(58)-C(51)-C(61)	115.7(2)
C(53)-O(52)-C(51)	113.81(18)
C(54)-C(53)-O(52)	119.8(2)
C(54)-C(53)-C(65)	129.1(2)
O(52)-C(53)-C(65)	110.9(2)
C(53)-C(54)-N(81)	121.0(2)
C(53)-C(54)-C(55)	121.8(2)
N(81)-C(54)-C(55)	117.1(2)
O(59)-C(55)-O(56)	107.12(19)
O(59)-C(55)-C(54)	107.9(2)
O(56)-C(55)-C(54)	113.1(2)
O(59)-C(55)-C(69)	106.2(2)

O(56)-C(55)-C(69)	106.0(2)	H(671)-C(67)-H(672)	109.5
C(54)-C(55)-C(69)	116.1(2)	C(65)-C(67)-H(673)	109.5
C(57)-O(56)-C(55)	113.37(18)	H(671)-C(67)-H(673)	109.5
C(58)-C(57)-O(56)	120.0(2)	H(672)-C(67)-H(673)	109.5
C(58)-C(57)-C(73)	130.3(3)	C(65)-C(68)-H(681)	109.5
O(56)-C(57)-C(73)	109.8(3)	C(65)-C(68)-H(682)	109.5
C(58)-C(57)-C(173)	128.8(4)	H(681)-C(68)-H(682)	109.5
O(56)-C(57)-C(173)	109.7(4)	C(65)-C(68)-H(683)	109.5
C(57)-C(58)-N(91)	122.4(2)	H(681)-C(68)-H(683)	109.5
C(57)-C(58)-C(51)	121.0(2)	H(682)-C(68)-H(683)	109.5
N(91)-C(58)-C(51)	116.6(2)	C(72)-C(69)-C(70)	110.9(2)
C(55)-O(59)-C(51)	109.92(18)	C(72)-C(69)-C(71)	108.9(2)
C(64)-C(61)-C(62)	110.3(2)	C(70)-C(69)-C(71)	107.1(2)
C(64)-C(61)-C(63)	109.3(2)	C(72)-C(69)-C(55)	109.7(2)
C(62)-C(61)-C(63)	107.2(2)	C(70)-C(69)-C(55)	112.0(2)
C(64)-C(61)-C(51)	109.3(2)	C(71)-C(69)-C(55)	108.2(2)
C(62)-C(61)-C(51)	112.4(2)	C(69)-C(70)-H(701)	109.5
C(63)-C(61)-C(51)	108.2(2)	C(69)-C(70)-H(702)	109.5
C(61)-C(62)-H(621)	109.5	H(701)-C(70)-H(702)	109.5
C(61)-C(62)-H(622)	109.5	C(69)-C(70)-H(703)	109.5
H(621)-C(62)-H(622)	109.5	H(701)-C(70)-H(703)	109.5
C(61)-C(62)-H(623)	109.5	H(702)-C(70)-H(703)	109.5
H(621)-C(62)-H(623)	109.5	C(69)-C(71)-H(711)	109.5
H(622)-C(62)-H(623)	109.5	C(69)-C(71)-H(712)	109.5
C(61)-C(63)-H(631)	109.5	H(711)-C(71)-H(712)	109.5
C(61)-C(63)-H(632)	109.5	C(69)-C(71)-H(713)	109.5
H(631)-C(63)-H(632)	109.5	H(711)-C(71)-H(713)	109.5
C(61)-C(63)-H(633)	109.5	H(712)-C(71)-H(713)	109.5
H(631)-C(63)-H(633)	109.5	C(69)-C(72)-H(721)	109.5
H(632)-C(63)-H(633)	109.5	C(69)-C(72)-H(722)	109.5
C(61)-C(64)-H(641)	109.5	H(721)-C(72)-H(722)	109.5
C(61)-C(64)-H(642)	109.5	C(69)-C(72)-H(723)	109.5
H(641)-C(64)-H(642)	109.5	H(721)-C(72)-H(723)	109.5
C(61)-C(64)-H(643)	109.5	H(722)-C(72)-H(723)	109.5
H(641)-C(64)-H(643)	109.5	C(76)-C(73)-C(74)	109.6(4)
H(642)-C(64)-H(643)	109.5	C(76)-C(73)-C(75)	107.9(4)
C(67)-C(65)-C(53)	114.1(2)	C(74)-C(73)-C(75)	107.6(4)
C(67)-C(65)-C(66)	107.5(3)	C(76)-C(73)-C(57)	115.3(4)
C(53)-C(65)-C(66)	110.0(2)	C(74)-C(73)-C(57)	103.8(3)
C(67)-C(65)-C(68)	109.8(2)	C(75)-C(73)-C(57)	112.3(4)
C(53)-C(65)-C(68)	107.6(2)	C(73)-C(74)-H(741)	109.5
C(66)-C(65)-C(68)	107.6(2)	C(73)-C(74)-H(742)	109.5
C(65)-C(66)-H(661)	109.5	H(741)-C(74)-H(742)	109.5
C(65)-C(66)-H(662)	109.5	C(73)-C(74)-H(743)	109.5
H(661)-C(66)-H(662)	109.5	H(741)-C(74)-H(743)	109.5
C(65)-C(66)-H(663)	109.5	H(742)-C(74)-H(743)	109.5
H(661)-C(66)-H(663)	109.5	C(73)-C(75)-H(751)	109.5
H(662)-C(66)-H(663)	109.5	C(73)-C(75)-H(752)	109.5
C(65)-C(67)-H(671)	109.5	H(751)-C(75)-H(752)	109.5
C(65)-C(67)-H(672)	109.5	C(73)-C(75)-H(753)	109.5

H(751)-C(75)-H(753)	109.5
H(752)-C(75)-H(753)	109.5
C(73)-C(76)-H(761)	109.5
C(73)-C(76)-H(762)	109.5
H(761)-C(76)-H(762)	109.5
C(73)-C(76)-H(763)	109.5
H(761)-C(76)-H(763)	109.5
H(762)-C(76)-H(763)	109.5
C(175)-C(173)-C(174)	109.5(7)
C(175)-C(173)-C(176)	108.3(8)
C(174)-C(173)-C(176)	107.8(8)
C(175)-C(173)-C(57)	114.9(6)
C(174)-C(173)-C(57)	106.7(6)
C(176)-C(173)-C(57)	109.4(6)
C(173)-C(174)-H(174)	109.5
C(173)-C(174)-H(274)	109.5
H(174)-C(174)-H(274)	109.5
C(173)-C(174)-H(374)	109.5
H(174)-C(174)-H(374)	109.5
H(274)-C(174)-H(374)	109.5
C(173)-C(175)-H(175)	109.5
C(173)-C(175)-H(275)	109.5
H(175)-C(175)-H(275)	109.5
C(173)-C(175)-H(375)	109.5
H(175)-C(175)-H(375)	109.5
H(275)-C(175)-H(375)	109.5
C(173)-C(176)-H(176)	109.5
C(173)-C(176)-H(276)	109.5
H(176)-C(176)-H(276)	109.5
C(173)-C(176)-H(376)	109.5
H(176)-C(176)-H(376)	109.5
H(276)-C(176)-H(376)	109.5
C(80)-N(81)-C(54)	125.6(2)
C(80)-N(81)-H(81)	110.6(17)
C(54)-N(81)-H(81)	120.7(17)
O(80)-C(80)-N(82)	122.4(2)
O(80)-C(80)-N(81)	119.7(2)
N(82)-C(80)-N(81)	117.9(2)
C(80)-N(82)-C(81)	120.3(2)
C(80)-N(82)-H(82)	119.4(17)
C(81)-N(82)-H(82)	118.8(17)
N(82)-C(81)-C(82)	113.1(2)
N(82)-C(81)-H(811)	109.0
C(82)-C(81)-H(811)	109.0
N(82)-C(81)-H(812)	109.0
C(82)-C(81)-H(812)	109.0
H(811)-C(81)-H(812)	107.8
C(81)-C(82)-C(83)	113.4(2)
C(81)-C(82)-H(821)	108.9
C(83)-C(82)-H(821)	108.9
C(81)-C(82)-H(822)	108.9
C(83)-C(82)-H(822)	108.9
H(821)-C(82)-H(822)	107.7
C(84)-C(83)-C(82)	114.6(2)
C(84)-C(83)-H(831)	108.6
C(82)-C(83)-H(831)	108.6
C(84)-C(83)-H(832)	108.6
C(82)-C(83)-H(832)	108.6
H(831)-C(83)-H(832)	107.6
C(83)-C(84)-C(85)	113.8(2)
C(83)-C(84)-H(841)	108.8
C(85)-C(84)-H(841)	108.8
C(83)-C(84)-H(842)	108.8
C(85)-C(84)-H(842)	108.8
H(841)-C(84)-H(842)	107.7
C(86)-C(85)-C(84)	113.2(3)
C(86)-C(85)-H(851)	108.9
C(84)-C(85)-H(851)	108.9
C(86)-C(85)-H(852)	108.9
C(84)-C(85)-H(852)	108.9
H(851)-C(85)-H(852)	107.8
C(85)-C(86)-H(861)	109.5
C(85)-C(86)-H(862)	109.5
H(861)-C(86)-H(862)	109.5
C(85)-C(86)-H(863)	109.5
H(861)-C(86)-H(863)	109.5
H(862)-C(86)-H(863)	109.5
C(90)-N(91)-C(58)	121.1(2)
C(90)-N(91)-H(91)	119.1(18)
C(58)-N(91)-H(91)	119.6(18)
O(90)-C(90)-N(92)	121.9(3)
O(90)-C(90)-N(91)	123.2(2)
N(92)-C(90)-N(91)	114.9(2)
C(90)-N(92)-C(191)	125.0(11)
C(90)-N(92)-C(91)	118.5(10)
C(90)-N(92)-H(92)	117.4(18)
C(191)-N(92)-H(92)	113(2)
C(91)-N(92)-H(92)	123(2)
N(92)-C(91)-C(92)	110.2(7)
N(92)-C(91)-H(911)	109.6
C(92)-C(91)-H(911)	109.6
N(92)-C(91)-H(912)	109.6
C(92)-C(91)-H(912)	109.6
H(911)-C(91)-H(912)	108.1
C(93)-C(92)-C(91)	117.5(9)
C(93)-C(92)-H(921)	107.9
C(91)-C(92)-H(921)	107.9
C(93)-C(92)-H(922)	107.9
C(91)-C(92)-H(922)	107.9
H(921)-C(92)-H(922)	107.2

C(92)-C(93)-C(94)	110.3(7)
C(92)-C(93)-H(931)	109.6
C(94)-C(93)-H(931)	109.6
C(92)-C(93)-H(932)	109.6
C(94)-C(93)-H(932)	109.6
H(931)-C(93)-H(932)	108.1
C(95)-C(94)-C(93)	114.2(7)
C(95)-C(94)-H(941)	108.7
C(93)-C(94)-H(941)	108.7
C(95)-C(94)-H(942)	108.7
C(93)-C(94)-H(942)	108.7
H(941)-C(94)-H(942)	107.6
C(94)-C(95)-C(96)	117(2)
C(94)-C(95)-H(951)	108.1
C(96)-C(95)-H(951)	108.1
C(94)-C(95)-H(952)	108.1
C(96)-C(95)-H(952)	108.1
H(951)-C(95)-H(952)	107.3
C(95)-C(96)-H(961)	109.5
C(95)-C(96)-H(962)	109.5
H(961)-C(96)-H(962)	109.5
C(95)-C(96)-H(963)	109.5
H(961)-C(96)-H(963)	109.5
H(962)-C(96)-H(963)	109.5
N(92)-C(191)-C(192)	116.8(7)
N(92)-C(191)-H(191)	108.1
C(192)-C(191)-H(191)	108.1
N(92)-C(191)-H(291)	108.1
C(192)-C(191)-H(291)	108.1
H(191)-C(191)-H(291)	107.3
C(193)-C(192)-C(191)	114.2(11)
C(193)-C(192)-H(192)	108.7
C(191)-C(192)-H(192)	108.7
C(193)-C(192)-H(292)	108.7
C(191)-C(192)-H(292)	108.7
H(192)-C(192)-H(292)	107.6
C(192)-C(193)-C(194)	117.2(8)
C(192)-C(193)-H(193)	108.0
C(194)-C(193)-H(193)	108.0
C(192)-C(193)-H(293)	108.0
C(194)-C(193)-H(293)	108.0
H(193)-C(193)-H(293)	107.2
C(195)-C(194)-C(193)	113.2(9)
C(195)-C(194)-H(194)	108.9
C(193)-C(194)-H(194)	108.9
C(195)-C(194)-H(294)	108.9
C(193)-C(194)-H(294)	108.9
H(194)-C(194)-H(294)	107.7
C(196)-C(195)-C(194)	113(2)
C(196)-C(195)-H(195)	108.9
C(194)-C(195)-H(195)	108.9
C(196)-C(195)-H(295)	108.9
C(194)-C(195)-H(295)	108.9
H(195)-C(195)-H(295)	107.7
C(195)-C(196)-H(196)	109.5
C(195)-C(196)-H(296)	109.5
H(196)-C(196)-H(296)	109.5
C(195)-C(196)-H(396)	109.5
H(196)-C(196)-H(396)	109.5
H(296)-C(196)-H(396)	109.5

O(9)-C(1)-O(2)-C(3)	50.7(2)
C(8)-C(1)-O(2)-C(3)	-68.2(2)
C(11)-C(1)-O(2)-C(3)	163.92(19)
C(1)-O(2)-C(3)-C(4)	-8.7(3)
C(1)-O(2)-C(3)-C(15)	173.5(2)
C(1)-O(2)-C(3)-C(115)	177.9(6)
O(2)-C(3)-C(4)-N(31)	169.9(2)
C(15)-C(3)-C(4)-N(31)	-12.8(4)
C(115)-C(3)-C(4)-N(31)	-20.8(11)
O(2)-C(3)-C(4)-C(5)	-14.6(3)
C(15)-C(3)-C(4)-C(5)	162.8(3)
C(115)-C(3)-C(4)-C(5)	154.8(10)
C(3)-C(4)-C(5)-O(9)	-5.6(3)
N(31)-C(4)-C(5)-O(9)	170.20(19)
C(3)-C(4)-C(5)-O(6)	112.9(2)
N(31)-C(4)-C(5)-O(6)	-71.3(3)
C(3)-C(4)-C(5)-C(19)	-124.6(3)
N(31)-C(4)-C(5)-C(19)	51.2(3)
O(9)-C(5)-O(6)-C(7)	52.4(2)
C(4)-C(5)-O(6)-C(7)	-66.8(2)
C(19)-C(5)-O(6)-C(7)	165.57(18)
C(5)-O(6)-C(7)-C(8)	-10.6(3)
C(5)-O(6)-C(7)-C(123)	173.7(5)
C(5)-O(6)-C(7)-C(23)	173.7(3)
O(6)-C(7)-C(8)-N(41)	166.3(2)
C(123)-C(7)-C(8)-N(41)	-19.2(7)
C(23)-C(7)-C(8)-N(41)	-19.1(5)
O(6)-C(7)-C(8)-C(1)	-13.0(3)
C(123)-C(7)-C(8)-C(1)	161.5(6)
C(23)-C(7)-C(8)-C(1)	161.6(3)
O(9)-C(1)-C(8)-C(7)	-5.9(3)
O(2)-C(1)-C(8)-C(7)	112.4(2)
C(11)-C(1)-C(8)-C(7)	-125.2(2)
O(9)-C(1)-C(8)-N(41)	174.73(18)
O(2)-C(1)-C(8)-N(41)	-66.9(3)
C(11)-C(1)-C(8)-N(41)	55.5(3)
O(6)-C(5)-O(9)-C(1)	-73.3(2)
C(4)-C(5)-O(9)-C(1)	48.1(2)
C(19)-C(5)-O(9)-C(1)	173.14(18)

O(2)-C(1)-O(9)-C(5)	-72.6(2)
C(8)-C(1)-O(9)-C(5)	48.1(2)
C(11)-C(1)-O(9)-C(5)	173.68(18)
O(9)-C(1)-C(11)-C(14)	43.5(3)
O(2)-C(1)-C(11)-C(14)	-70.9(3)
C(8)-C(1)-C(11)-C(14)	164.2(2)
O(9)-C(1)-C(11)-C(13)	-75.1(2)
O(2)-C(1)-C(11)-C(13)	170.5(2)
C(8)-C(1)-C(11)-C(13)	45.5(3)
O(9)-C(1)-C(11)-C(12)	162.2(2)
O(2)-C(1)-C(11)-C(12)	47.7(3)
C(8)-C(1)-C(11)-C(12)	-77.2(3)
C(4)-C(3)-C(15)-C(17)	-0.4(5)
O(2)-C(3)-C(15)-C(17)	177.2(3)
C(115)-C(3)-C(15)-C(17)	157(3)
C(4)-C(3)-C(15)-C(18)	124.5(3)
O(2)-C(3)-C(15)-C(18)	-58.0(3)
C(115)-C(3)-C(15)-C(18)	-78(3)
C(4)-C(3)-C(15)-C(16)	-120.0(3)
O(2)-C(3)-C(15)-C(16)	57.5(3)
C(115)-C(3)-C(15)-C(16)	37(3)
C(4)-C(3)-C(115)-C(117)	-175.2(10)
O(2)-C(3)-C(115)-C(117)	-4.5(14)
C(15)-C(3)-C(115)-C(117)	156(4)
C(4)-C(3)-C(115)-C(118)	-54.4(17)
O(2)-C(3)-C(115)-C(118)	116.2(11)
C(15)-C(3)-C(115)-C(118)	-83(3)
C(4)-C(3)-C(115)-C(116)	59.8(16)
O(2)-C(3)-C(115)-C(116)	-129.5(10)
C(15)-C(3)-C(115)-C(116)	31(3)
O(9)-C(5)-C(19)-C(21)	-75.8(2)
O(6)-C(5)-C(19)-C(21)	169.96(19)
C(4)-C(5)-C(19)-C(21)	44.5(3)
O(9)-C(5)-C(19)-C(20)	160.7(2)
O(6)-C(5)-C(19)-C(20)	46.4(3)
C(4)-C(5)-C(19)-C(20)	-79.0(3)
O(9)-C(5)-C(19)-C(22)	42.2(3)
O(6)-C(5)-C(19)-C(22)	-72.1(2)
C(4)-C(5)-C(19)-C(22)	162.5(2)
C(8)-C(7)-C(23)-C(25)	-112.1(6)
O(6)-C(7)-C(23)-C(25)	62.9(6)
C(123)-C(7)-C(23)-C(25)	64(27)
C(8)-C(7)-C(23)-C(26)	131.1(8)
O(6)-C(7)-C(23)-C(26)	-53.9(9)
C(123)-C(7)-C(23)-C(26)	-53(27)
C(8)-C(7)-C(23)-C(24)	7.7(8)
O(6)-C(7)-C(23)-C(24)	-177.2(7)
C(123)-C(7)-C(23)-C(24)	-177(100)
C(8)-C(7)-C(123)-C(125)	-123.4(12)
O(6)-C(7)-C(123)-C(125)	51.6(13)
C(23)-C(7)-C(123)-C(125)	-128(28)
C(8)-C(7)-C(123)-C(126)	113.0(17)
O(6)-C(7)-C(123)-C(126)	-72.0(18)
C(23)-C(7)-C(123)-C(126)	109(28)
C(8)-C(7)-C(123)-C(124)	-9.3(16)
O(6)-C(7)-C(123)-C(124)	165.7(13)
C(23)-C(7)-C(123)-C(124)	-14(27)
C(3)-C(4)-N(31)-C(30)	-80.8(3)
C(5)-C(4)-N(31)-C(30)	103.4(3)
C(4)-N(31)-C(30)-O(30)	-172.1(2)
C(4)-N(31)-C(30)-N(32)	8.5(4)
O(30)-C(30)-N(32)-C(31)	-3.1(4)
N(31)-C(30)-N(32)-C(31)	176.4(2)
C(30)-N(32)-C(31)-C(32)	-81.4(3)
N(32)-C(31)-C(32)-C(33)	168.5(2)
C(31)-C(32)-C(33)-C(34)	68.0(3)
C(32)-C(33)-C(34)-C(35)	174.2(2)
C(33)-C(34)-C(35)-C(36)	-179.1(3)
C(7)-C(8)-N(41)-C(40)	-76.1(3)
C(1)-C(8)-N(41)-C(40)	103.2(3)
C(8)-N(41)-C(40)-O(40)	4.6(4)
C(8)-N(41)-C(40)-N(42)	-175.9(2)
O(40)-C(40)-N(42)-C(41)	-2.3(4)
N(41)-C(40)-N(42)-C(41)	178.2(2)
C(40)-N(42)-C(41)-C(42)	103.1(3)
N(42)-C(41)-C(42)-C(43)	-64.3(3)
C(41)-C(42)-C(43)-C(144)	166.1(10)
C(41)-C(42)-C(43)-C(44)	-172.5(3)
C(42)-C(43)-C(44)-C(45)	-175.3(3)
C(144)-C(43)-C(44)-C(45)	-48(2)
C(43)-C(44)-C(45)-C(46)	69.6(4)
C(42)-C(43)-C(144)-C(145)	-162.4(13)
C(44)-C(43)-C(144)-C(145)	135(4)
C(43)-C(144)-C(145)-C(146)	-176.2(16)
O(59)-C(51)-O(52)-C(53)	-51.8(2)
C(58)-C(51)-O(52)-C(53)	67.3(2)
C(61)-C(51)-O(52)-C(53)	-165.73(19)
C(51)-O(52)-C(53)-C(54)	10.4(3)
C(51)-O(52)-C(53)-C(65)	-174.25(19)
O(52)-C(53)-C(54)-N(81)	-172.2(2)
C(65)-C(53)-C(54)-N(81)	13.4(4)
O(52)-C(53)-C(54)-C(55)	12.5(4)
C(65)-C(53)-C(54)-C(55)	-162.0(2)
C(53)-C(54)-C(55)-O(59)	6.9(3)
N(81)-C(54)-C(55)-O(59)	-168.6(2)
C(53)-C(54)-C(55)-O(56)	-111.3(3)
N(81)-C(54)-C(55)-O(56)	73.2(3)
C(53)-C(54)-C(55)-C(69)	125.8(3)
N(81)-C(54)-C(55)-C(69)	-49.7(3)
O(59)-C(55)-O(56)-C(57)	-53.6(2)

C(54)-C(55)-O(56)-C(57)	65.1(3)
C(69)-C(55)-O(56)-C(57)	-166.66(19)
C(55)-O(56)-C(57)-C(58)	11.5(3)
C(55)-O(56)-C(57)-C(73)	-169.6(3)
C(55)-O(56)-C(57)-C(173)	178.6(4)
O(56)-C(57)-C(58)-N(91)	-165.7(2)
C(73)-C(57)-C(58)-N(91)	15.6(5)
C(173)-C(57)-C(58)-N(91)	29.8(6)
O(56)-C(57)-C(58)-C(51)	13.4(4)
C(73)-C(57)-C(58)-C(51)	-165.2(3)
C(173)-C(57)-C(58)-C(51)	-151.0(5)
O(59)-C(51)-C(58)-C(57)	4.8(3)
O(52)-C(51)-C(58)-C(57)	-113.9(2)
C(61)-C(51)-C(58)-C(57)	124.2(3)
O(59)-C(51)-C(58)-N(91)	-175.94(19)
O(52)-C(51)-C(58)-N(91)	65.3(3)
C(61)-C(51)-C(58)-N(91)	-56.6(3)
O(56)-C(55)-O(59)-C(51)	73.7(2)
C(54)-C(55)-O(59)-C(51)	-48.3(2)
C(69)-C(55)-O(59)-C(51)	-173.34(19)
O(52)-C(51)-O(59)-C(55)	73.2(2)
C(58)-C(51)-O(59)-C(55)	-47.4(2)
C(61)-C(51)-O(59)-C(55)	-172.54(19)
O(59)-C(51)-C(61)-C(64)	74.2(2)
O(52)-C(51)-C(61)-C(64)	-170.6(2)
C(58)-C(51)-C(61)-C(64)	-46.5(3)
O(59)-C(51)-C(61)-C(62)	-162.9(2)
O(52)-C(51)-C(61)-C(62)	-47.8(3)
C(58)-C(51)-C(61)-C(62)	76.3(3)
O(59)-C(51)-C(61)-C(63)	-44.7(3)
O(52)-C(51)-C(61)-C(63)	70.4(2)
C(58)-C(51)-C(61)-C(63)	-165.4(2)
C(54)-C(53)-C(65)-C(67)	-57.2(4)
O(52)-C(53)-C(65)-C(67)	128.0(3)
C(54)-C(53)-C(65)-C(66)	-178.0(3)
O(52)-C(53)-C(65)-C(66)	7.1(3)
C(54)-C(53)-C(65)-C(68)	64.9(4)
O(52)-C(53)-C(65)-C(68)	-109.9(2)
O(59)-C(55)-C(69)-C(72)	75.9(3)
O(56)-C(55)-C(69)-C(72)	-170.4(2)
C(54)-C(55)-C(69)-C(72)	-43.9(3)
O(59)-C(55)-C(69)-C(70)	-160.5(2)
O(56)-C(55)-C(69)-C(70)	-46.8(3)
C(54)-C(55)-C(69)-C(70)	79.7(3)
O(59)-C(55)-C(69)-C(71)	-42.7(3)
O(56)-C(55)-C(69)-C(71)	71.0(3)
C(54)-C(55)-C(69)-C(71)	-162.5(2)
C(58)-C(57)-C(73)-C(76)	-24.2(6)
O(56)-C(57)-C(73)-C(76)	157.1(4)
C(173)-C(57)-C(73)-C(76)	-111(3)
C(58)-C(57)-C(73)-C(74)	95.7(5)
O(56)-C(57)-C(73)-C(74)	-83.1(4)
C(173)-C(57)-C(73)-C(74)	9(2)
C(58)-C(57)-C(73)-C(75)	-148.4(4)
O(56)-C(57)-C(73)-C(75)	32.9(4)
C(173)-C(57)-C(73)-C(75)	125(3)
C(58)-C(57)-C(173)-C(175)	10.1(10)
O(56)-C(57)-C(173)-C(175)	-155.6(6)
C(73)-C(57)-C(173)-C(175)	112(3)
C(58)-C(57)-C(173)-C(174)	-111.5(7)
O(56)-C(57)-C(173)-C(174)	82.8(7)
C(73)-C(57)-C(173)-C(174)	-9(2)
C(58)-C(57)-C(173)-C(176)	132.1(6)
O(56)-C(57)-C(173)-C(176)	-33.6(8)
C(73)-C(57)-C(173)-C(176)	-126(3)
C(53)-C(54)-N(81)-C(80)	93.0(3)
C(55)-C(54)-N(81)-C(80)	-91.5(3)
C(54)-N(81)-C(80)-O(80)	164.1(3)
C(54)-N(81)-C(80)-N(82)	-17.2(4)
O(80)-C(80)-N(82)-C(81)	6.5(4)
N(81)-C(80)-N(82)-C(81)	-172.1(2)
C(80)-N(82)-C(81)-C(82)	78.1(3)
N(82)-C(81)-C(82)-C(83)	-169.6(2)
C(81)-C(82)-C(83)-C(84)	-69.3(3)
C(82)-C(83)-C(84)-C(85)	-173.3(2)
C(83)-C(84)-C(85)-C(86)	176.5(3)
C(57)-C(58)-N(91)-C(90)	83.0(3)
C(51)-C(58)-N(91)-C(90)	-96.2(3)
C(58)-N(91)-C(90)-O(90)	-5.5(4)
C(58)-N(91)-C(90)-N(92)	174.1(2)
O(90)-C(90)-N(92)-C(191)	-18.2(6)
N(91)-C(90)-N(92)-C(191)	162.3(5)
O(90)-C(90)-N(92)-C(91)	-2.3(6)
N(91)-C(90)-N(92)-C(91)	178.1(5)
C(90)-N(92)-C(91)-C(92)	-71.9(14)
C(191)-N(92)-C(91)-C(92)	48(6)
N(92)-C(91)-C(92)-C(93)	-58.4(18)
C(91)-C(92)-C(93)-C(94)	163.0(10)
C(92)-C(93)-C(94)-C(95)	-169.7(9)
C(93)-C(94)-C(95)-C(96)	169(3)
C(90)-N(92)-C(191)-C(192)	-95.3(18)
C(91)-N(92)-C(191)-C(192)	-164(9)
N(92)-C(191)-C(192)-C(193)	61(2)
C(191)-C(192)-C(193)-C(194)	-177.9(9)
C(192)-C(193)-C(194)-C(195)	170.5(8)
C(193)-C(194)-C(195)-C(196)	177(3)

Table S4: Crystal data and structure refinement for **5 (KRS45)**.

Crystal data	
Identification code	KRS45
Empirical formula	$C_{26}H_{44}N_2O_7$
Formula weight	496.63
Crystal description	block, colourless
Crystal size	0.38 x 0.32 x 0.23mm
Crystal system, space group	monoclinic, C 2/c
Unit cell dimensions:	a 16.8698(6)Å
b	10.4411(6)Å
c	17.1497(7)Å
β	113.903(3)°
Volume	2761.7(2)Å^3
Z	4
Calculated density	1.194Mg/m^3
F(000)	1080
Linear absorption coefficient μ	0.086mm^{-1}
Absorption correction	semi-empirical from equivalents
Max. and min. transmission	0.7456 and 0.6336
Unit cell determination	2.64° < Θ < 27.51°
	5368 reflections used at 100K
Data collection	
Temperature	100K
Diffractometer	Bruker APEX-II CCD
Radiation source	fine-focus sealed tube
Radiation and wavelength	MoK_α, 0.71073Å
Monochromator	graphite
Scan type	ϕ and ω scans
Θ range for data collection	2.36 to 27.50°
Index ranges	$-18 \le h \le 21$, $-13 \le k \le 13$, $-22 \le l \le 22$
Reflections collected / unique	11810 / 3165
Significant unique reflections	2622 with I > 2σ(I)
R(int), R(sigma)	0.0254, 0.0222
Completeness to Θ = 27.5°	99.7%
Refinement	
Refinement method	Full-matrix least-squares on F^2
Data / parameters / restraints	3165 / 177 / 0
Goodness-of-fit on F^2	1.045
Final R indices [I > 2σ(I)]	R1 = 0.0377, wR2 = 0.0964
R indices (all data)	R1 = 0.0484, wR2 = 0.1058
Extinction expression	none
Weighting scheme	$w = 1/[\sigma^2(F_o^2)+(aP)^2+bP]$ where $P = (F_o^2+2F_c^2)/3$
Weighting scheme parameters a, b	0.0505, 2.2527

Largest Δ/σ in last cycle	0.001
Largest difference peak and hole	0.408 and -0.240e/Å^3
Structure Solution Program	SHELXS-97 (Sheldrick, 2008)
Structure Refinement Program	SHELXL-97 (Sheldrick, 2008)

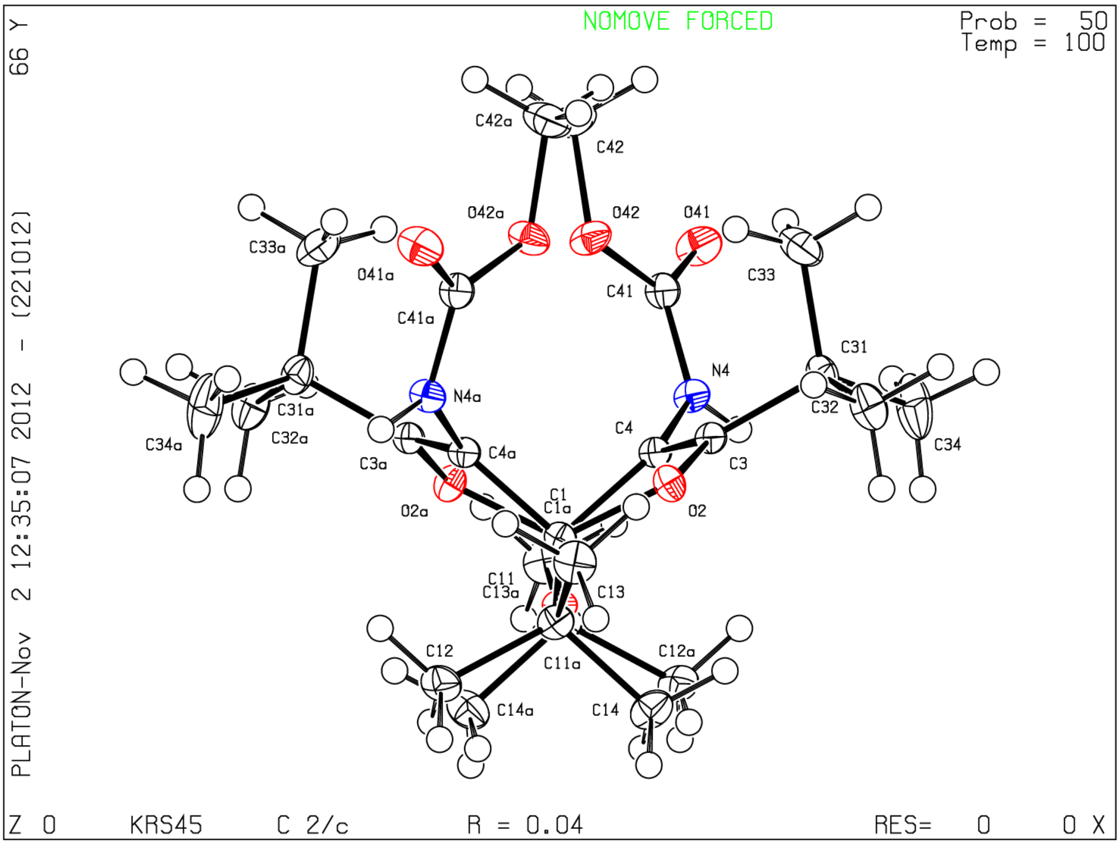

Table S5: Hydrogen bonds for **5 (KRS45)** [Å, °].

D-H···A	d(D-H)	d(H···A)	d(D···A)	<(DHA)
N(4)-H(4)···O(41)$^{i)}$	0.871(17)	2.022(17)	2.8905(13)	175.0(15)

Symmetry transformation used to generate the equivalent atom:

$^{i)}$ 1/2-x, 3/2-y, 1-z

Table S6: Full list of bond lengths [Å] and angles [°] for **5 (KRS45)**.

C(1)-O(9)	1.4104(12)	O(2)-C(3)	1.3676(13)
C(1)-O(2)	1.4425(13)	C(3)-C(4)	1.3454(16)
C(1)-C(4)$^{i)}$	1.5252(15)	C(3)-C(31)	1.5353(16)
C(1)-C(11)	1.5580(15)	C(4)-N(4)	1.4270(14)

C(4)-C(1)$^{i)}$	1.5252(15)
O(9)-C(1)$^{i)}$	1.4104(12)
C(11)-C(12)	1.5353(16)
C(11)-C(13)	1.5415(17)
C(11)-C(14)	1.5439(16)
C(12)-H(121)	0.98
C(12)-H(122)	0.98
C(12)-H(123)	0.98
C(13)-H(131)	0.98
C(13)-H(132)	0.98
C(13)-H(133)	0.98
C(14)-H(141)	0.98
C(14)-H(142)	0.98
C(14)-H(143)	0.98
C(31)-C(33)	1.5324(18)
C(31)-C(34)	1.5379(17)
C(31)-C(32)	1.5440(18)
C(32)-H(321)	0.98
C(32)-H(322)	0.98
C(32)-H(323)	0.98
C(33)-H(331)	0.98
C(33)-H(332)	0.98
C(33)-H(333)	0.98
C(34)-H(341)	0.98
C(34)-H(342)	0.98
C(34)-H(343)	0.98
N(4)-C(41)	1.3462(15)
N(4)-H(4)	0.871(17)
C(41)-O(41)	1.2259(14)
C(41)-O(42)	1.3474(14)
O(42)-C(42)	1.4433(15)
C(42)-H(421)	0.98
C(42)-H(422)	0.98
C(42)-H(423)	0.98

O(9)-C(1)-O(2)	108.47(8)
O(9)-C(1)-C(4)$^{i)}$	107.85(8)
O(2)-C(1)-C(4)$^{i)}$	112.01(9)
O(9)-C(1)-C(11)	106.05(9)
O(2)-C(1)-C(11)	105.09(9)
C(4)$^{i)}$-C(1)-C(11)	116.97(9)
C(3)-O(2)-C(1)	115.57(8)
C(4)-C(3)-O(2)	120.60(10)
C(4)-C(3)-C(31)	128.61(10)
O(2)-C(3)-C(31)	110.79(9)
C(3)-C(4)-N(4)	120.75(10)
C(3)-C(4)-C(1)$^{i)}$	120.76(10)
N(4)-C(4)-C(1)$^{i)}$	118.23(9)
C(1)-O(9)-C(1)$^{i)}$	111.46(12)
C(12)-C(11)-C(13)	110.35(9)
C(12)-C(11)-C(14)	109.22(10)
C(13)-C(11)-C(14)	107.66(10)
C(12)-C(11)-C(1)	109.36(9)
C(13)-C(11)-C(1)	112.22(9)
C(14)-C(11)-C(1)	107.95(9)
C(11)-C(12)-H(121)	109.5
C(11)-C(12)-H(122)	109.5
H(121)-C(12)-H(122)	109.5
C(11)-C(12)-H(123)	109.5
H(121)-C(12)-H(123)	109.5
H(122)-C(12)-H(123)	109.5
C(11)-C(13)-H(131)	109.5
C(11)-C(13)-H(132)	109.5
H(131)-C(13)-H(132)	109.5
C(11)-C(13)-H(133)	109.5
H(131)-C(13)-H(133)	109.5
H(132)-C(13)-H(133)	109.5
C(11)-C(14)-H(141)	109.5
C(11)-C(14)-H(142)	109.5
H(141)-C(14)-H(142)	109.5
C(11)-C(14)-H(143)	109.5
H(141)-C(14)-H(143)	109.5
H(142)-C(14)-H(143)	109.5
C(33)-C(31)-C(3)	109.71(10)
C(33)-C(31)-C(34)	110.69(12)
C(3)-C(31)-C(34)	111.97(10)
C(33)-C(31)-C(32)	107.76(11)
C(3)-C(31)-C(32)	109.71(10)
C(34)-C(31)-C(32)	106.86(11)
C(31)-C(32)-H(321)	109.5
C(31)-C(32)-H(322)	109.5
H(321)-C(32)-H(322)	109.5
C(31)-C(32)-H(323)	109.5
H(321)-C(32)-H(323)	109.5
H(322)-C(32)-H(323)	109.5
C(31)-C(33)-H(331)	109.5
C(31)-C(33)-H(332)	109.5
H(331)-C(33)-H(332)	109.5
C(31)-C(33)-H(333)	109.5
H(331)-C(33)-H(333)	109.5
H(332)-C(33)-H(333)	109.5
C(31)-C(34)-H(341)	109.5
C(31)-C(34)-H(342)	109.5
H(341)-C(34)-H(342)	109.5
C(31)-C(34)-H(343)	109.5
H(341)-C(34)-H(343)	109.5
H(342)-C(34)-H(343)	109.5
C(41)-N(4)-C(4)	126.74(10)
C(41)-N(4)-H(4)	113.3(11)
C(4)-N(4)-H(4)	119.9(11)

O(41)-C(41)-N(4)	123.53(11)
O(41)-C(41)-O(42)	122.65(11)
N(4)-C(41)-O(42)	113.81(10)
C(41)-O(42)-C(42)	114.05(9)
O(42)-C(42)-H(421)	109.5
O(42)-C(42)-H(422)	109.5
H(421)-C(42)-H(422)	109.5
O(42)-C(42)-H(423)	109.5
H(421)-C(42)-H(423)	109.5
H(422)-C(42)-H(423)	109.5

O(9)-C(1)-O(2)-C(3)	44.14(12)
C(4)[i)]-C(1)-O(2)-C(3)	-74.79(11)
C(11)-C(1)-O(2)-C(3)	157.22(9)
C(1)-O(2)-C(3)-C(4)	-4.84(14)
C(1)-O(2)-C(3)-C(31)	175.67(9)
O(2)-C(3)-C(4)-N(4)	173.72(9)
C(31)-C(3)-C(4)-N(4)	-6.89(18)
O(2)-C(3)-C(4)-C(1)[i)]	-12.22(16)
C(31)-C(3)-C(4)-C(1)[i)]	167.16(10)
O(2)-C(1)-O(9)-C(1)[i)]	-70.04(7)
C(4)[i)]-C(1)-O(9)-C(1)[i)]	51.47(6)
C(11)-C(1)-O(9)-C(1)[i)]	177.52(10)
O(9)-C(1)-C(11)-C(12)	-66.28(10)
O(2)-C(1)-C(11)-C(12)	178.95(9)
C(4)[i)]-C(1)-C(11)-C(12)	54.01(13)
O(9)-C(1)-C(11)-C(13)	170.93(8)
O(2)-C(1)-C(11)-C(13)	56.15(11)
C(4)[i)]-C(1)-C(11)-C(13)	-68.78(13)
O(9)-C(1)-C(11)-C(14)	52.45(11)
O(2)-C(1)-C(11)-C(14)	-62.32(11)
C(4)[i)]-C(1)-C(11)-C(14)	172.74(10)
C(4)-C(3)-C(31)-C(33)	76.50(15)
O(2)-C(3)-C(31)-C(33)	-104.07(12)
C(4)-C(3)-C(31)-C(34)	-46.82(17)
O(2)-C(3)-C(31)-C(34)	132.61(12)
C(4)-C(3)-C(31)-C(32)	-165.30(12)
O(2)-C(3)-C(31)-C(32)	14.14(13)
C(3)-C(4)-N(4)-C(41)	-86.47(15)
C(1)[i)]-C(4)-N(4)-C(41)	99.33(13)
C(4)-N(4)-C(41)-O(41)	178.45(11)
C(4)-N(4)-C(41)-O(42)	-0.77(17)
O(41)-C(41)-O(42)-C(42)	-8.14(17)
N(4)-C(41)-O(42)-C(42)	171.08(11)

Symmetry transformation used to generate equivalent atoms:

[i)] 1-x, y, 3/2-z

Calculated Energies and Cartesian Coordinates (B3LYP/6-31G**)

Calculations were carried out at the B3LYP/6-31G** level using the Gaussian suite of programs [6]. The temperature used was 298.15 K, and calculated wavenumbers were scaled by a factor 0.9613 [7].

Table S7: Summary of calculated energies (B3LYP/6-31G**), energy difference (kcal/mol) with respect to the most stable isomer, point group symmetry and strongest calculated IR frequencies (B3LYP/6-31G**, scaled by 0.9613, percent relative intensity).

	HF +ZPVE (Hartree)	ΔE (kcal/mol) cf. most stable isomer (bold)	Sym.	Strongest calculated IR (cm^{-1}) (scaled 0.9613, percent relative intensity)
**3**	-1620.615076		C2	1714.6(82), 1581.8(65), 1274.2, 1233.5(71), 1071.4(100)
**4a**	-2005.867297	**0.0**	C1	1703.9(53), 1679.5(47), 1514.6(100)
**4b**	-2005.861057	3.9	C2	1711.4(100), 1499.1(73)
**4c**	-2005.858582	5.5	C1	1718.2(65), 1495.0(63), 1493.2(100), 1172.3(60)
**5**	-1652.720565		C2	1738.7(100), 1327.0(62)
**7a-a**	-3263.911428	**0.0**	C1	1658.8(31), 1587.7(37), 1491.0(100), 1256.7(66)
**7a-b**	-3263.891924	12.2	C1	1662.1(48), 1587.2(42), 1491.6(100), 1255.9(63)
**7a-c**	-3263.877985	21.0	C2	1661.2(50), 1589.6(38), 1494.7(61), 1253.8(100)
**7c-a**	-3876.272146	**0.0**	C2	1662.8(37), 1589.1(41), 1496.7(100), 1249.4(48)
**7c-b**	-3876.252573	12.3	C1	1657.3(47), 1587.8(48), 1498.8(95), 1247.6(100)
**7c-c**	-3876.245337	16.8	C1	1660.5(30), 1588.4(31), 1507.3(38), 1245.9(100)

Diethyl 1,3,5,7-tetra-tert-butyl-2,6,9-trioxabicyclo[3.3.1]nona-3,7-diene-4,8-dicarboxylate, Compound 3.

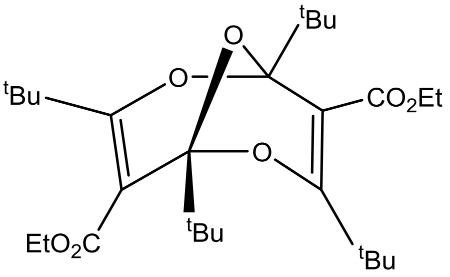

Atom Type	Coordinates (Angstroms) X	Y	Z
C	-1.09168697	0.39458963	1.12686954
O	-1.63768341	-0.79839643	0.53528113
C	-0.74006755	-1.56240134	-0.14555470
C	0.59312878	-1.36109253	0.03499945
O	0.00000000	0.00000000	1.92385888
C	-2.17346754	0.94931438	2.11566018
C	-1.65951850	2.25684407	2.74880141
H	-2.38183761	2.61119878	3.49174676
H	-1.53132282	3.04290641	1.99991308
H	-0.70161477	2.10367685	3.25228831
C	-3.52122052	1.20980213	1.41211851
H	-4.27768763	1.42538335	2.17365022
H	-3.85303261	0.34327669	0.83695168

H	-3.48464649	2.07572127	0.74909613
C	-2.41006480	-0.09845144	3.22700146
H	-3.17690500	0.27310561	3.91460821
H	-1.50196115	-0.28943619	3.80156662
H	-2.75737249	-1.04687237	2.80949852
C	-1.46117664	-2.56620223	-1.06904189
C	-1.28642001	-3.99860298	-0.51178241
H	-1.82043453	-4.70976365	-1.15198495
H	-1.70884165	-4.07448115	0.49578187
H	-0.23675778	-4.28931049	-0.46599166
C	-2.97293777	-2.24985704	-1.12859566
H	-3.45170860	-2.96563487	-1.80501019
H	-3.15213511	-1.23935084	-1.50060268
H	-3.44585402	-2.34435073	-0.14830405
C	-0.92121690	-2.46909502	-2.51508489
H	-1.52357975	-3.11163877	-3.16601093
H	0.11456433	-2.79716905	-2.60876743
H	-0.99708225	-1.44298145	-2.88575791
C	1.63336413	-1.88724727	-0.89806256
O	2.28250943	-1.18715898	-1.65038735
O	1.82870446	-3.22760180	-0.81188067
C	2.82567178	-3.77782814	-1.70938466
H	3.77638452	-3.26674082	-1.53067052
H	2.52759912	-3.56363782	-2.74079174
C	2.91554644	-5.26673662	-1.43783705
H	3.66761364	-5.71669377	-2.09331557
H	1.95791045	-5.76035910	-1.62591133
H	3.20485938	-5.45929629	-0.40086522
C	1.09168697	-0.39458963	1.12686954
O	1.63768341	0.79839643	0.53528113
C	0.74006755	1.56240134	-0.14555470
C	-0.59312878	1.36109253	0.03499945
C	2.17346754	-0.94931438	2.11566018
C	1.65951850	-2.25684407	2.74880141
H	2.38183761	-2.61119878	3.49174676
H	1.53132282	-3.04290641	1.99991308
H	0.70161477	-2.10367685	3.25228831
C	3.52122052	-1.20980213	1.41211851
H	4.27768763	-1.42538335	2.17365022
H	3.85303261	-0.34327669	0.83695168
H	3.48464649	-2.07572127	0.74909613
C	2.41006480	0.09845144	3.22700146
H	3.17690500	-0.27310561	3.91460821
H	1.50196115	0.28943619	3.80156662
H	2.75737249	1.04687237	2.80949852
C	1.46117664	2.56620223	-1.06904189
C	1.28642001	3.99860298	-0.51178241
H	1.82043453	4.70976365	-1.15198495
H	1.70884165	4.07448115	0.49578187
H	0.23675778	4.28931049	-0.46599166
C	2.97293777	2.24985704	-1.12859566
H	3.45170860	2.96563487	-1.80501019
H	3.15213511	1.23935084	-1.50060268
H	3.44585402	2.34435073	-0.14830405
C	0.92121690	2.46909502	-2.51508489
H	1.52357975	3.11163877	-3.16601093
H	-0.11456433	2.79716905	-2.60876743
H	0.99708225	1.44298145	-2.88575791
C	-1.63336413	1.88724727	-0.89806256
O	-2.28250943	1.18715898	-1.65038735
O	-1.82870446	3.22760180	-0.81188067

C	-2.82567178	3.77782814	-1.70938466
H	-3.77638452	3.26674082	-1.53067052
H	-2.52759912	3.56363782	-2.74079174
C	-2.91554644	5.26673662	-1.43783705
H	-3.66761364	5.71669377	-2.09331557
H	-1.95791045	5.76035910	-1.62591133
H	-3.20485938	5.45929629	-0.40086522

Point Group= C2. State= 1-A.
HF= -1621.3221448. RMSD=5.068e-09. RMSF=3.221e-06.
Zero-point correction= 0.707069 (Hartree/Particle)
Sum of electronic and zero-point Energies= -1620.615076

Calculated IR spectrum (unscaled Harmonic frequencies (cm-1)):

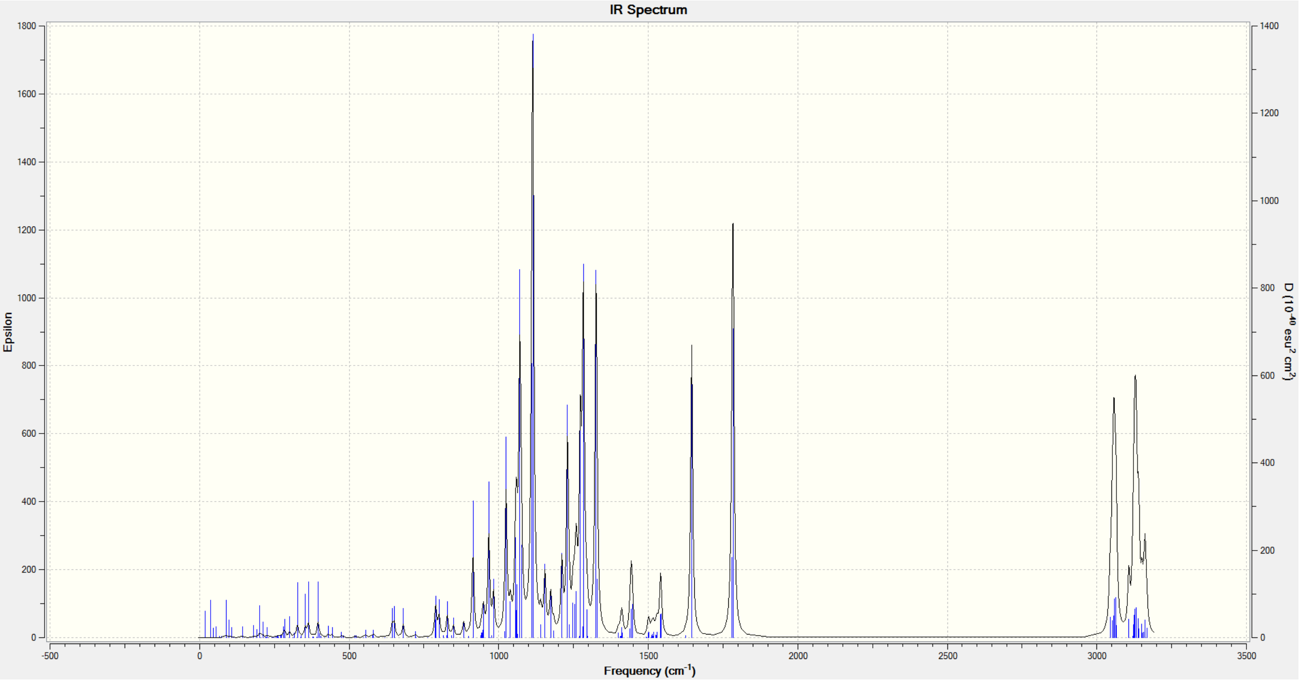

1,3,5,7-Tetra-*tert*-butyl-2,6,9-trioxabi-cyclo[3.3.1]nona-3,7-diene-4,8-diyl-bis(3-hexylurea), Compound 4a.

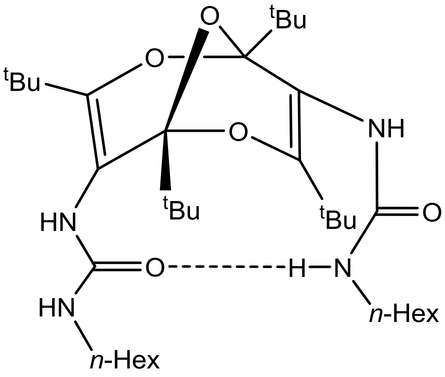

Atom	Coordinates (Angstroms)		
Type	X	Y	Z

C	-2.84191486	-1.19508722	0.80057798
O	-2.41684378	-0.28368742	1.82371917
C	-2.13101120	0.98937384	1.40733771
C	-2.38561749	1.35736929	0.12311037
C	-3.27073081	0.46841700	-0.77203119
O	-2.51632094	-0.17800679	-1.82521447
C	-1.68012783	-1.17460165	-1.42566492
C	-1.68892158	-1.58247890	-0.13068143
O	-3.83237984	-0.55374956	0.02609594
C	-3.56725457	-2.37851105	1.54198385
C	-2.73997994	-2.92358801	2.72662040
C	-3.87151572	-3.50580794	0.53744057
C	-4.90182990	-1.85633908	2.12329453
C	-1.60213619	1.78618327	2.61095103
C	-2.75591145	1.83395038	3.64894917
C	-1.17689764	3.23577799	2.31724513
C	-0.39714041	1.03989013	3.23202754
C	-4.47378939	1.17834702	-1.49204592
C	-4.01757769	2.01101418	-2.71191686
C	-5.23472951	2.06178624	-0.48335821
C	-5.44524057	0.09618519	-2.02016819
C	-0.84938391	-1.68115598	-2.61574608
C	-0.24757609	-3.08549034	-2.40374253
C	-1.79300423	-1.77389034	-3.84401752
C	0.28192461	-0.67813700	-2.92871732
N	-1.94752188	2.59559997	-0.40214075
C	-0.71213811	2.93094446	-0.96029971
O	-0.60152780	3.99735063	-1.56590703
N	0.31065076	2.05134141	-0.75631384
C	1.65179055	2.33446414	-1.24177454
C	2.68045768	2.46178633	-0.11129946
C	4.10349966	2.69790659	-0.63087728
C	5.14667375	2.82858322	0.48540086
C	6.57534957	3.04710896	-0.02731315
C	7.60783990	3.18483482	1.09564043
N	-0.62040174	-2.34463797	0.40374986
C	0.60014306	-1.79140810	0.74763852
O	0.90167603	-0.61473328	0.53211329
N	1.46442327	-2.67064719	1.35777955
C	2.85670875	-2.30977565	1.60352081
C	3.78679618	-2.57381908	0.41229953
C	5.24310976	-2.18853359	0.69778012
C	6.18236460	-2.44797676	-0.48637576
C	7.63843198	-2.05154889	-0.21345435
C	8.56884927	-2.31701959	-1.40063007
H	-2.50574695	-2.13228534	3.44127412
H	-3.32695344	-3.68794242	3.24660559
H	-1.79741475	-3.38790046	2.42987263
H	-4.41887031	-3.12122553	-0.32689126
H	-2.96345514	-3.99336083	0.16654395
H	-4.48578880	-4.27502933	1.01674487
H	-4.73594273	-1.00209499	2.78446037
H	-5.59285415	-1.54927658	1.33697131
H	-5.37739304	-2.65153198	2.70686192
H	-2.41766741	2.36499153	4.54495667
H	-3.62365552	2.36796594	3.24753078
H	-3.07475442	0.83244397	3.94494153
H	-0.32321289	3.28629316	1.64022023
H	-1.98807550	3.83050824	1.89382700
H	-0.88033704	3.69938511	3.26485161
H	0.42099766	0.94532024	2.51402080

H	-0.03584986	1.59680674	4.10309937
H	-0.67419036	0.03548134	3.55927162
H	-3.56712946	1.37055908	-3.47328786
H	-4.89634840	2.49090968	-3.15600973
H	-3.29480150	2.79076743	-2.47350322
H	-4.65705404	2.93096153	-0.15469442
H	-6.15183745	2.43932061	-0.94672899
H	-5.51470585	1.48969986	0.40542991
H	-4.92589384	-0.61912470	-2.66276251
H	-5.92348981	-0.45510810	-1.20875117
H	-6.22891106	0.57826665	-2.61364160
H	0.15822288	-3.43871090	-3.35757640
H	0.56597436	-3.09445059	-1.67802626
H	-1.00782777	-3.80462838	-2.08108437
H	-1.22556627	-2.14712513	-4.70237461
H	-2.62104309	-2.46679874	-3.65941440
H	-2.21354917	-0.80306128	-4.10705906
H	0.82370990	-0.99847060	-3.82560883
H	-0.11918329	0.32242877	-3.10663077
H	0.98652455	-0.61938471	-2.09594042
H	-2.62711534	3.31135991	-0.61411257
H	0.16052591	1.17621569	-0.26449634
H	1.96780960	1.54160485	-1.93321803
H	1.58678127	3.26366603	-1.81256949
H	2.65463095	1.54685174	0.49450524
H	2.38233575	3.28748237	0.54787685
H	4.12065209	3.60562258	-1.25070441
H	4.38950616	1.87143768	-1.29784163
H	4.86813748	3.66144361	1.14657164
H	5.12214535	1.92557685	1.11328853
H	6.59902990	3.94595186	-0.65819889
H	6.85623418	2.21111671	-0.68280950
H	8.61623804	3.33948312	0.69797070
H	7.37327746	4.03478683	1.74646436
H	7.63264749	2.28713536	1.72436545
H	-0.80112978	-3.29170675	0.70422320
H	1.24996395	-3.65674113	1.31527491
H	2.86734208	-1.24833057	1.86009788
H	3.19703483	-2.86562282	2.48497673
H	3.41388866	-2.00697130	-0.44936377
H	3.73397155	-3.63722062	0.13803612
H	5.28848813	-1.12478691	0.97057279
H	5.60442280	-2.74316019	1.57586292
H	6.14147801	-3.51312034	-0.75627229
H	5.81633994	-1.89902086	-1.36543591
H	7.67844605	-0.98650310	0.05223880
H	8.00331014	-2.59739490	0.66734274
H	9.59875981	-2.02177034	-1.17600232
H	8.57847765	-3.38012243	-1.66715949
H	8.24887454	-1.75819787	-2.28731540

Point Group= C1. State= 1-A.
HF=-2006.8620195. RMSD=7.478e-09. RMSF=3.802e-06.
Zero-point correction= 0.994722 (Hartree/Particle)
Sum of electronic and zero-point Energies= -2005.867297

Calculated IR spectrum (unscaled Harmonic frequencies (cm-1)):

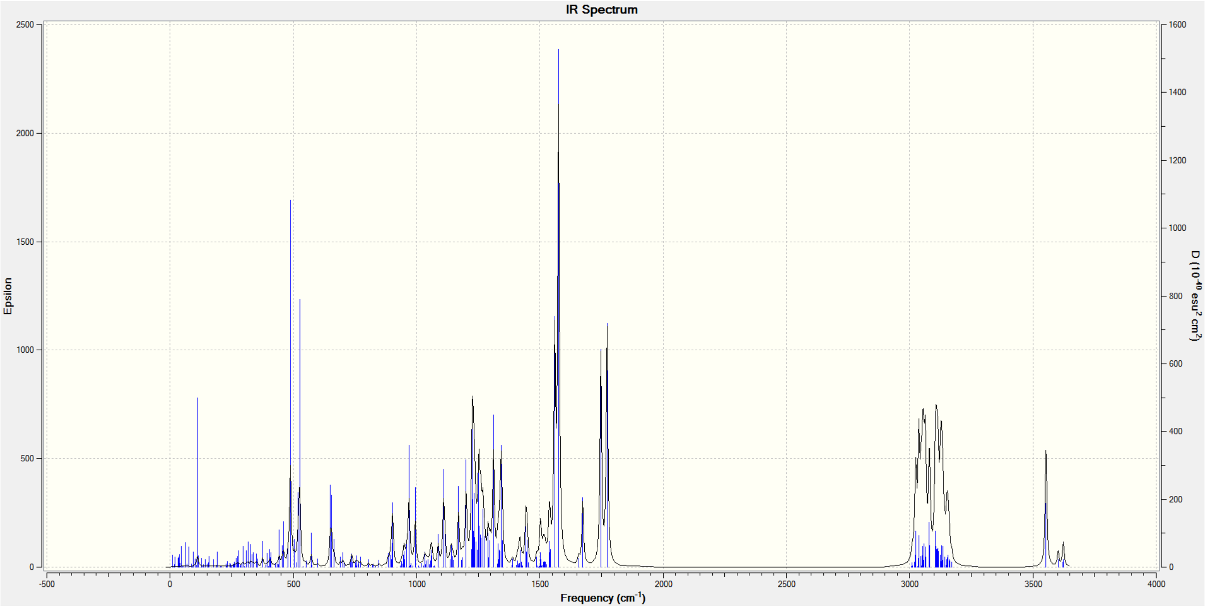

1,3,5,7-Tetra-*tert*-butyl-2,6,9-trioxabi-cyclo[3.3.1]nona-3,7-diene-4,8-diyl-bis(3-hexylurea), Compound 4b.

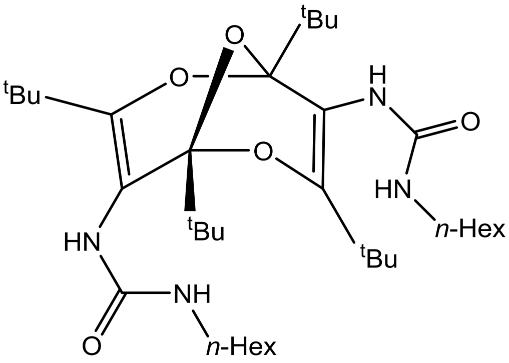

Atom Type	Coordinates (Angstroms) X	Y	Z
C	2.91042338	-1.15321222	-0.21277161
O	2.24528094	-1.00690465	-1.49316968
C	1.70636806	0.20669820	-1.77002154
C	1.89148359	1.26467786	-0.93151890
C	2.91042345	1.15321204	0.21277152
O	2.24528100	1.00690451	1.49316958
C	1.70636805	-0.20669830	1.77002145
C	1.89148351	-1.26467798	0.93151881
O	3.69358956	-0.00000012	-0.00000005
C	3.92527912	-2.33958607	-0.39203228
C	3.25456143	-3.61738600	-0.94452448
H	2.71843703	-3.41004468	-1.87339656
H	4.03734110	-4.34952294	-1.16948018
H	2.55690229	-4.09005180	-0.25360539
C	4.62311521	-2.63442379	0.95047142
H	5.10619619	-1.73967484	1.35147331
H	3.93677114	-3.01700046	1.71166682
H	5.39364177	-3.39733635	0.80076481
C	5.00217972	-1.90858768	-1.41624748
H	4.55145444	-1.65278747	-2.37852444

H	5.57767507	-1.04977268	-1.06716587
H	5.69416554	-2.74084431	-1.57831545
C	0.96266520	0.19790105	-3.12360507
C	1.65018692	1.18714980	-4.09353640
H	1.17864234	1.12123133	-5.07983528
H	1.56247967	2.21962639	-3.75124544
H	2.71144745	0.94656184	-4.21279996
C	-0.52265008	0.58881103	-2.95218576
H	-1.03836463	-0.10015574	-2.27464985
H	-0.64080436	1.60332214	-2.57355459
H	-1.02653474	0.52792019	-3.92272829
C	1.01145448	-1.20960774	-3.75819838
H	0.55207880	-1.96183809	-3.11284473
H	0.46423229	-1.18594513	-4.70597719
H	2.03591593	-1.52710868	-3.96685919
C	3.92527928	2.33958583	0.39203219
C	3.25456167	3.61738580	0.94452439
H	2.71843725	3.41004450	1.87339646
H	4.03734138	4.34952269	1.16948009
H	2.55690256	4.09005164	0.25360530
C	4.62311538	2.63442349	-0.95047151
H	3.93677134	3.01700021	-1.71166691
H	5.39364199	3.39733600	-0.80076490
H	5.10619630	1.73967451	-1.35147340
C	5.00217984	1.90858736	1.41624739
H	4.55145455	1.65278718	2.37852435
H	5.57767514	1.04977233	1.06716578
H	5.69416571	2.74084395	1.57831536
C	0.96266519	-0.19790111	3.12360498
C	1.65018684	-1.18714990	4.09353631
H	1.17864227	-1.12123140	5.07983518
H	1.56247952	-2.21962648	3.75124535
H	2.71144739	-0.94656202	4.21279987
C	1.01145456	1.20960768	3.75819829
H	0.46423237	1.18594510	4.70597710
H	2.03591603	1.52710855	3.96685910
H	0.55207893	1.96183806	3.11284463
C	-0.52265012	-0.58881099	2.95218567
H	-1.02653477	-0.52792011	3.92272820
H	-1.03836462	0.10015582	2.27464976
H	-0.64080447	-1.60332209	2.57355450
N	1.29058282	2.52292704	-1.22017241
H	1.84653494	3.21968581	-1.69861946
C	0.23862289	3.14626130	-0.54841750
O	0.03701976	4.34869834	-0.69936527
N	-0.53145260	2.34175592	0.24872922
H	-0.36729125	1.34714238	0.20752574
C	-1.77783488	2.83331489	0.82001120
H	-1.91936736	2.36373578	1.80096785
H	-1.64210212	3.90512433	0.98212927
C	-3.00706012	2.58993526	-0.06579410
H	-3.10653442	1.51299892	-0.26522070
H	-2.83669312	3.07497724	-1.03482676
C	-4.30226217	3.11782865	0.56210264
H	-4.19746719	4.19499139	0.75263668
H	-4.45072736	2.64856045	1.54550707
C	-5.54309396	2.87509842	-0.30569017
H	-5.39339662	3.33992631	-1.29039919
H	-5.65044822	1.79688343	-0.49335719
C	-6.83934604	3.41072539	0.31469063
H	-6.73202951	4.48790308	0.50070959

H	-6.98741391	2.94689361	1.29945590
C	-8.07357653	3.16338318	-0.55770812
H	-8.98121317	3.55738378	-0.08922039
H	-7.96921628	3.64449549	-1.53681125
H	-8.22778389	2.09239247	-0.73211624
N	1.29058266	-2.52292712	1.22017232
H	1.84653473	-3.21968592	1.69861937
C	0.23862268	-3.14626131	0.54841740
O	0.03701947	-4.34869834	0.69936518
N	-0.53145276	-2.34175588	-0.24872931
H	-0.36729134	-1.34714235	-0.20752583
C	-1.77783506	-2.83331477	-0.82001129
H	-1.91936751	-2.36373565	-1.80096795
H	-1.64210238	-3.90512422	-0.98212936
C	-3.00706029	-2.58993506	0.06579401
H	-3.10653452	-1.51299871	0.26522060
H	-2.83669332	-3.07497705	1.03482667
C	-4.30226238	-3.11782837	-0.56210273
H	-4.45072753	-2.64856016	-1.54550716
H	-4.19746746	-4.19499111	-0.75263677
H	-6.98741410	-2.94689315	-1.29945599
C	-5.54309415	-2.87509806	0.30569008
H	-5.39339684	-3.33992595	1.29039910
H	-5.65044833	-1.79688305	0.49335710
C	-6.83934627	-3.41072494	-0.31469072
H	-6.73202981	-4.48790264	-0.50070968
C	-8.07357673	-3.16338265	0.55770803
H	-8.98121340	-3.55738319	0.08922029
H	-7.96921652	-3.64449496	1.53681115
H	-8.22778402	-2.09239193	0.73211615

Point Group= C2. State= 1-A.
HF= -2006.8551078. RMSD=5.985e-09. RMSF=2.264e-06.
Zero-point correction= 0.994051 (Hartree/Particle)
Sum of electronic and zero-point Energies= -2005.861057

Calculated IR spectrum (unscaled Harmonic frequencies (cm-1)):

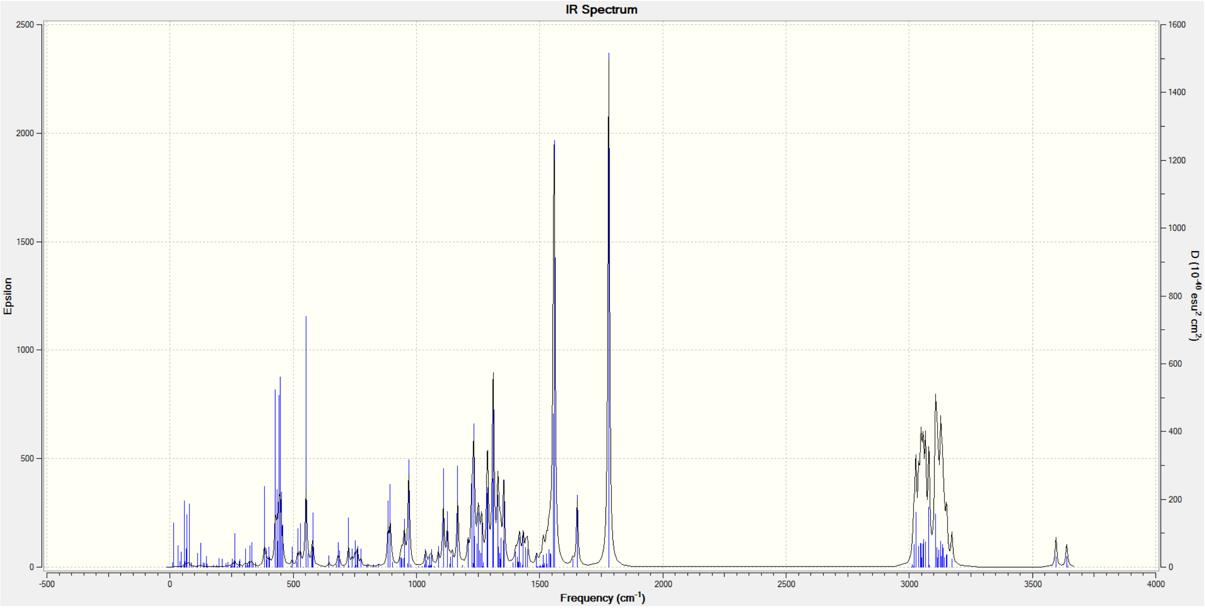

1,3,5,7-Tetra-*tert*-butyl-2,6,9-trioxabi-cyclo[3.3.1]nona-3,7-diene-4,8-diyl-bis(3-hexylurea), Compound 4c.

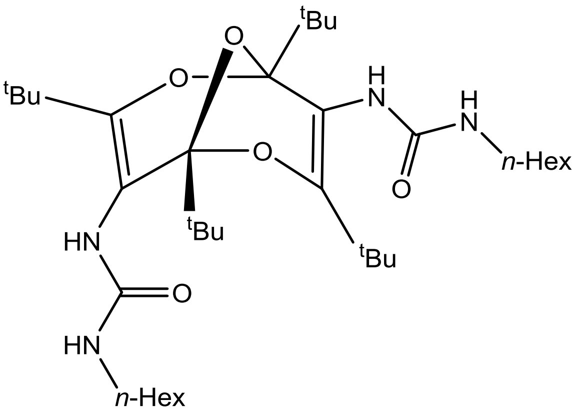

Atom Type	Coordinates (Angstroms) X	Y	Z
C	-1.16920275	2.25371276	-0.00079002
O	-1.27016559	1.61171399	1.28690487
C	-0.14369488	0.99225164	1.73040257
C	1.04925579	1.21149216	1.12071180
C	1.17011171	2.25341685	0.00414783
O	1.27091068	1.61302454	-1.28435934
C	0.14428252	0.99440905	-1.72864121
C	-1.04861700	1.21318594	-1.11867864
O	0.00055323	3.04655386	0.00217733
C	-2.36783603	3.26563557	-0.07719180
C	-3.72076547	2.60616719	0.26537762
H	-3.68262805	2.12002891	1.24230997
H	-4.49168687	3.38350501	0.30328085
H	-4.04174885	1.86286119	-0.46465034
C	-2.41590912	3.90117029	-1.47993410
H	-1.44970427	4.33681705	-1.74658029
H	-2.68387829	3.18142028	-2.26032279
H	-3.16805176	4.69670166	-1.50039402
C	-2.13249173	4.38641669	0.96325890
H	-2.02220366	3.96953692	1.96746975
H	-1.24091932	4.97267290	0.73547820
H	-2.99442170	5.06179901	0.96730952
C	-0.42528856	0.10453656	2.95819859
C	0.39851109	0.59850652	4.16914839
H	0.14187710	0.00695593	5.05489391
H	1.47295803	0.49419384	4.00259907
H	0.18333173	1.64869742	4.39440897
C	-0.08897413	-1.37228854	2.64356137
H	-0.61989741	-1.69877583	1.74762068
H	0.97922668	-1.52606950	2.49150020
H	-0.40382779	-1.99838175	3.48678096
C	-1.92226283	0.17019889	3.33497218
H	-2.54510240	-0.19692649	2.51750818
H	-2.08912553	-0.46306883	4.21275305
H	-2.23820809	1.18657856	3.58645843
C	2.36897683	3.26497494	0.08181619
C	3.72179499	2.60561436	-0.26140620
H	3.68361950	2.12057073	-1.23888082
H	4.49287223	3.38284182	-0.29840006
H	4.04258883	1.86143492	0.46781272

C	2.41707269	3.89892049	1.48527785
H	2.68486718	3.17825097	2.26487561
H	3.16935177	4.69429814	1.50668009
H	1.45092655	4.33443583	1.75235245
C	2.13396208	4.38699111	-0.95737320
H	2.02365324	3.97127648	-1.96206507
H	1.24250389	4.97318699	-0.72898995
H	2.99604428	5.06218370	-0.96059315
C	0.42566937	0.10821472	-2.95758332
C	-0.39784540	0.60408309	-4.16795446
H	-0.14132508	0.01363510	-5.05446898
H	-1.47234454	0.49991268	-4.00165554
H	-0.18228837	1.65450237	-4.39178387
C	1.92270003	0.17386805	-3.33412733
H	2.08942134	-0.45824285	-4.21276791
H	2.23902606	1.19048342	-3.58417707
H	2.54533233	-0.19460557	-2.51711127
C	0.08882315	-1.36891247	-2.64494216
H	0.40359250	-1.99399469	-3.48894191
H	0.61951234	-1.69676280	-1.74935948
H	-0.97945133	-1.52253770	-2.49324118
N	2.22023388	0.52795063	1.55776182
H	2.82855511	1.02891513	2.19175757
C	2.78973560	-0.54450848	0.88360270
O	2.26912546	-1.10603426	-0.07200882
N	4.01759611	-0.91862853	1.40120292
H	4.26452322	-0.57879470	2.31980660
C	4.69749137	-2.12372051	0.94534758
H	4.59792714	-2.92473196	1.69327579
H	4.16536157	-2.45288527	0.05012250
C	6.17850364	-1.87714986	0.64076863
H	6.67080448	-1.45680311	1.53003256
H	6.25427440	-1.11308667	-0.14283985
C	6.91378760	-3.15166882	0.21050538
H	6.41724508	-3.57690497	-0.67298426
H	6.82293336	-3.90924544	1.00217020
C	8.39752706	-2.92334300	-0.10244581
H	8.48903775	-2.17754157	-0.90477900
H	8.89076208	-2.48333576	0.77639257
C	9.14181090	-4.20004643	-0.51158924
H	8.64838568	-4.64132091	-1.38819515
H	9.05181013	-4.94362724	0.29202479
C	10.62225575	-3.96333554	-0.82445241
H	11.12487815	-4.89300619	-1.11030130
H	10.74467137	-3.25251354	-1.64957526
H	11.15056613	-3.55353437	0.04400981
N	-2.21980277	0.53059544	-1.55665867
H	-2.82794126	1.03259967	-2.19000795
C	-2.78970170	-0.54253087	-0.88389798
O	-2.26931429	-1.10547583	0.07099821
N	-4.01769643	-0.91552873	-1.40198985
H	-4.26449178	-0.57441642	-2.32015516
C	-4.69802573	-2.12097105	-0.94770864
H	-4.59866907	-2.92107639	-1.69663426
H	-4.16607583	-2.45143750	-0.05285661
C	-6.17897818	-1.87428711	-0.64293370
H	-6.67107285	-1.45266614	-1.53170852
H	-6.25455292	-1.11117756	0.14162244
C	-6.91472087	-3.14909570	-0.21431575
H	-6.82407559	-3.90570912	-1.00692483
H	-6.41837659	-3.57560849	0.66866984

H	-9.05335266	-4.94022070	-0.29821538
C	-8.39840138	-2.92065817	0.09883357
H	-8.48970575	-2.17583462	0.90209803
H	-8.89143411	-2.47937865	-0.77948023
C	-9.14314462	-4.19762094	0.50632852
H	-8.64991980	-4.64016672	1.38240626
C	-10.62352604	-3.96079769	0.81940680
H	-11.12648196	-4.89065517	1.10405884
H	-10.74574483	-3.25097172	1.64541583
H	-11.15164795	-3.54972452	-0.04856874

Point Group= C1. State= 1-A.
HF= -2006.8524225. RMSD=4.019e-09. RMSF=2.594e-06
Zero-point correction= 0.9938408 (Hartree/Particle)
Sum of electronic and zero-point Energies= -2005.858582

Calculated IR spectrum (unscaled Harmonic frequencies (cm-1)):

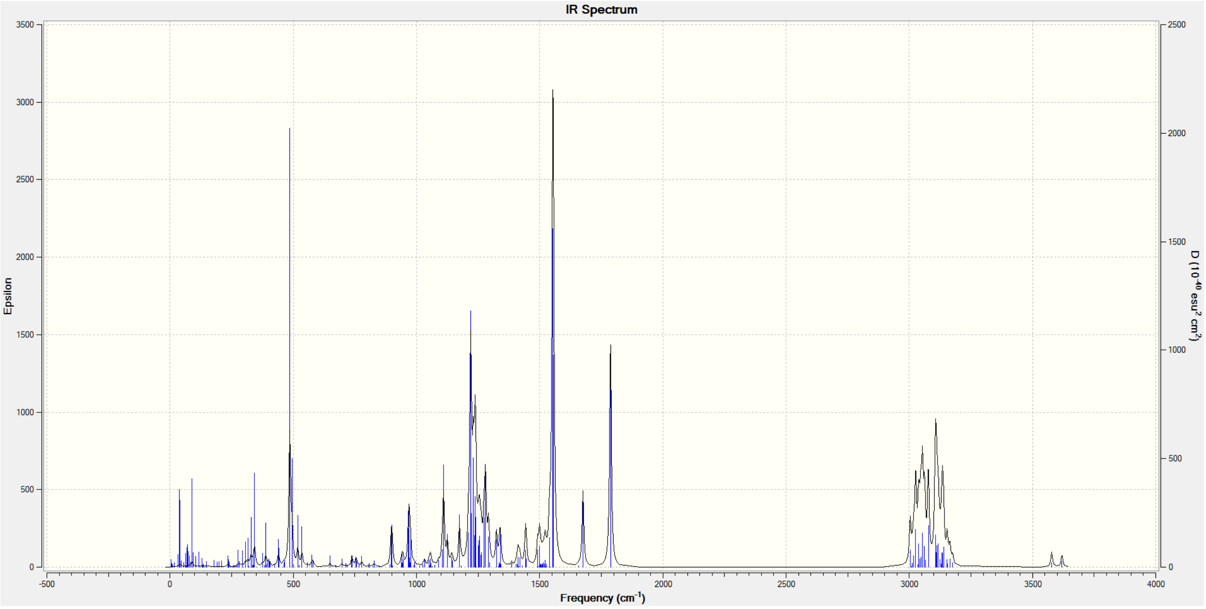

Dimethyl (1,3,5,7-tetra-*tert*-butyl-2,6,9-trioxabi-cyclo[3.3.1]nona-3,7-diene-4,8-diyl)-biscarbamate, Compound 5.

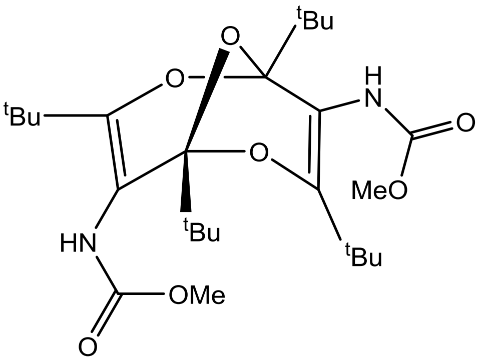

Atom Type	X	Y	Z
C	1.16827604	0.08004084	1.25124098
C	-1.16827604	-0.08004084	1.25124098

(Coordinates (Angstroms))

O	1.17719818	1.36952546	0.59523748
O	-1.17719818	-1.36952546	0.59523748
C	0.00965091	1.75687927	0.01774251
C	-0.00965091	-1.75687927	0.01774251
C	-1.13312695	1.05425809	0.22361702
C	1.13312695	-1.05425809	0.22361702
O	0.00000000	0.00000000	2.03924809
C	2.36937415	0.10049034	2.26405631
C	-2.36937415	-0.10049034	2.26405631
C	2.51752238	-1.28906259	2.91359531
C	-2.51752238	1.28906259	2.91359531
H	3.26860048	-1.24518869	3.70867312
H	-3.26860048	1.24518869	3.70867312
H	1.57347665	-1.62029846	3.35364177
H	-1.57347665	1.62029846	3.35364177
H	2.84457503	-2.05485887	2.20361669
H	-2.84457503	2.05485887	2.20361669
C	3.69580479	0.53341494	1.60192743
C	-3.69580479	-0.53341494	1.60192743
H	4.06384685	-0.16511232	0.85051557
H	-4.06384685	0.16511232	0.85051557
H	3.59650781	1.51405630	1.13124739
H	-3.59650781	-1.51405630	1.13124739
H	4.46405946	0.61233275	2.37841195
H	-4.46405946	-0.61233275	2.37841195
C	2.05693798	1.13142189	3.37471421
C	-2.05693798	-1.13142189	3.37471421
H	1.87638016	2.12263037	2.95102312
H	-1.87638016	-2.12263037	2.95102312
H	1.18381064	0.84563888	3.96354587
H	-1.18381064	-0.84563888	3.96354587
H	2.91595705	1.20245328	4.04946720
H	-2.91595705	-1.20245328	4.04946720
C	0.18020549	3.05844459	-0.79124417
C	-0.18020549	-3.05844459	-0.79124417
C	1.66642112	3.47836108	-0.81973927
C	-1.66642112	-3.47836108	-0.81973927
H	2.28981779	2.70995119	-1.28193971
H	-2.28981779	-2.70995119	-1.28193971
H	1.76150592	4.40011941	-1.40226517
H	-1.76150592	-4.40011941	-1.40226517
H	2.05646789	3.66855099	0.18278701
H	-2.05646789	-3.66855099	0.18278701
C	-0.29140481	2.87712446	-2.25376964
C	0.29140481	-2.87712446	-2.25376964
H	-1.37790079	2.84466405	-2.33325934
H	1.37790079	-2.84466405	-2.33325934
H	0.06032185	3.72286912	-2.85463293
H	-0.06032185	-3.72286912	-2.85463293
H	0.10977451	1.95555063	-2.68216225
H	-0.10977451	-1.95555063	-2.68216225
C	-0.62810853	4.19059375	-0.11642391
H	-0.30195700	4.34680888	0.91695195
H	-0.47852270	5.12908350	-0.66136666
H	-1.69947367	3.97773877	-0.11236241
N	-2.34548813	1.43769117	-0.41655839
N	2.34548813	-1.43769117	-0.41655839
H	-2.99735732	2.02415323	0.08719403
H	2.99735732	-2.02415323	0.08719403
C	-2.94659442	0.88784871	-1.52220082
C	2.94659442	-0.88784871	-1.52220082

O	-4.08176621	1.17075815	-1.86505381
O	4.08176621	-1.17075815	-1.86505381
O	-2.13943793	0.03684046	-2.19858965
O	2.13943793	-0.03684046	-2.19858965
C	0.62810853	-4.19059375	-0.11642391
H	0.47852270	-5.12908350	-0.66136666
H	1.69947367	-3.97773877	-0.11236241
H	0.30195700	-4.34680888	0.91695195
C	2.72681903	0.48080045	-3.40385329
H	1.97294608	1.13647383	-3.83819347
H	3.63831855	1.04037004	-3.18217171
H	2.97157922	-0.33083707	-4.09290537
C	-2.72681903	-0.48080045	-3.40385329
H	-1.97294608	-1.13647383	-3.83819347
H	-3.63831855	-1.04037004	-3.18217171
H	-2.97157922	0.33083707	-4.09290537

Point Group= C2. State= 1-A.
HF=-1653.4059296. RMSD= 5.631e-09. RMSF= 6.935e-06.
Zero-point correction= 0.685364 (Hartree/Particle)
Sum of electronic and zero-point Energies= -1652.720565

Calculated IR spectrum (unscaled Harmonic frequencies (cm-1)):

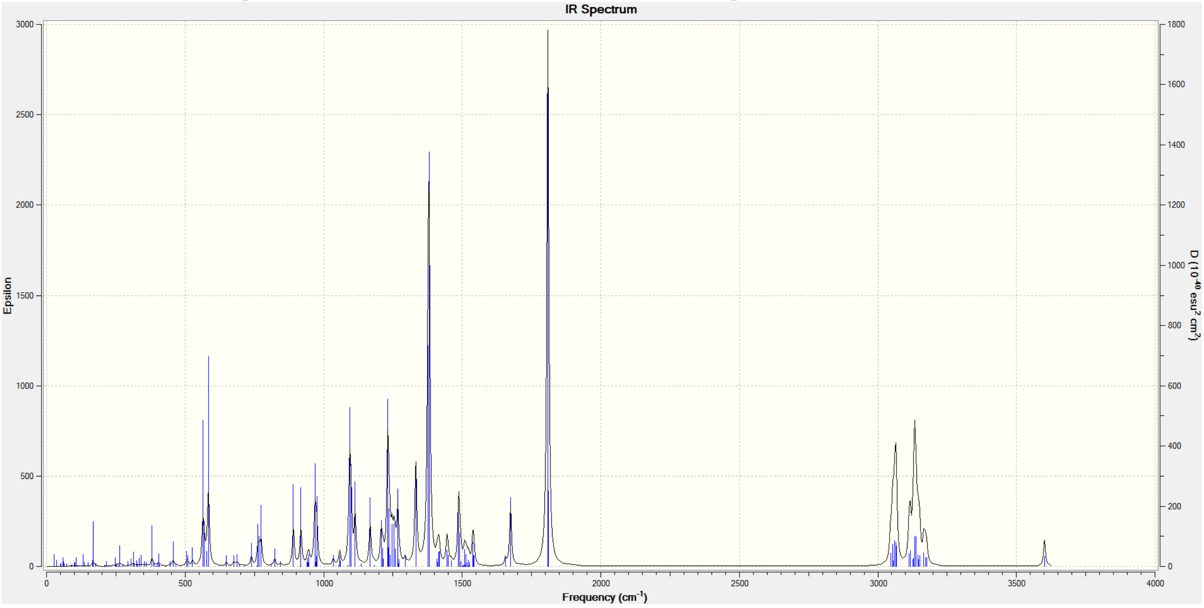

(1,3,5,7-Tetra-tert-butyl-2,6,9-trioxabicyclo[3.3.1]nona-3,7-diene)-4,8-dicarbonic acid-(benzo-15-crown-5)-4’-yl-amide, Compound 7a-a.

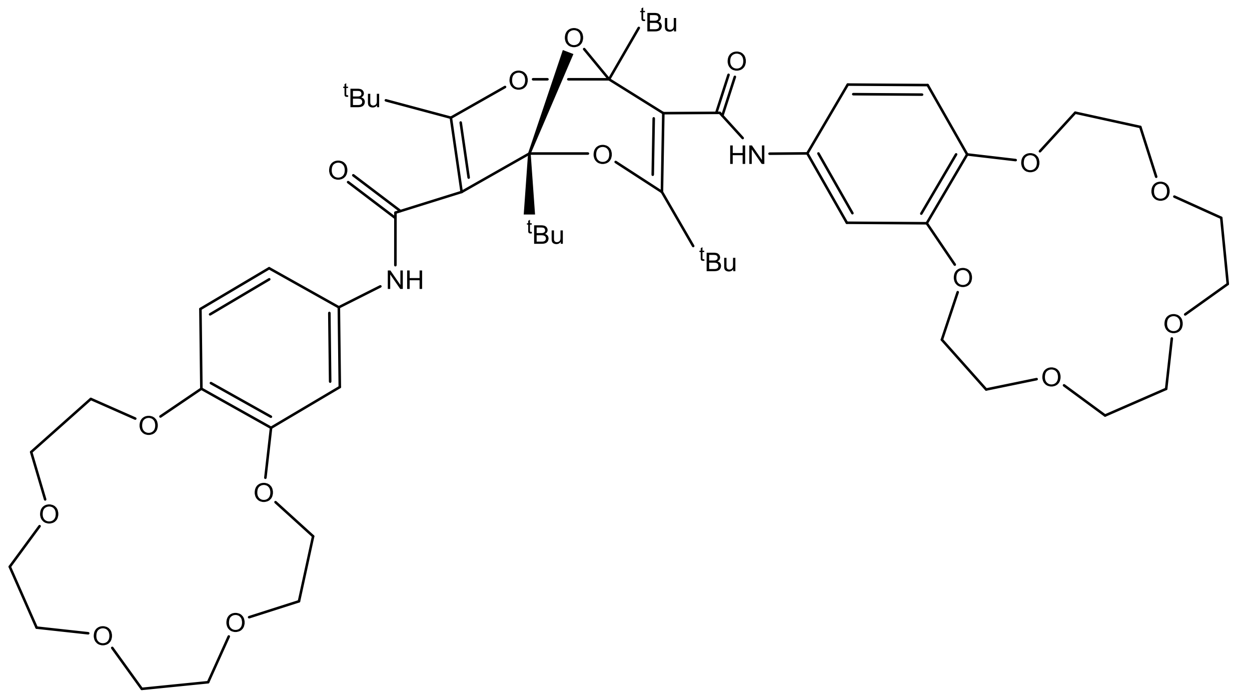

Atom Type	Coordinates (Angstroms) X	Y	Z
O	-0.00076800	4.53471900	0.00319600
C	1.14999300	3.75349700	0.20635400
C	-1.15154400	3.75391900	-0.20147100
C	2.31278800	4.77313000	0.48102800
C	-2.31433700	4.77408000	-0.47414600
C	-0.85741600	2.72004200	-1.29704400
C	0.85584400	2.71750100	1.29993800
C	-0.42124100	2.52700000	1.72103300
C	-0.92004400	1.74780200	2.95850200
O	-1.46824500	3.09180200	1.05158100
C	0.41964300	2.53035000	-1.71855100
O	1.46668300	3.09381500	-1.04800300
C	0.91832900	1.75349400	-2.95753900
C	0.56169000	2.57239500	-4.22389100
C	2.45312500	1.58225400	-2.91836000
C	0.29191100	0.34251500	-3.04832100
C	2.29648700	5.84473100	-0.63367700
C	2.08016600	5.45192600	1.84582400
C	3.70370800	4.10286600	0.46606000
C	-2.29802500	5.84348400	0.64266800
C	-2.08174300	5.45551700	-1.83762900
C	-3.70525900	4.10379900	-0.46048800
C	-0.29351300	0.33670800	3.04682300
C	-0.56373300	2.56442400	4.22640900
C	-2.45482000	1.57645800	2.91872400
C	-2.01520500	2.00483500	-1.94637000
C	2.01356300	2.00117600	1.94814500
O	-2.42125100	2.25331500	-3.08268800
O	2.41920000	2.24748800	3.08507900
N	2.57506200	1.04540600	1.14254400
N	-2.57631000	1.04745000	-1.14241900
H	1.02930500	3.56196600	-4.18724000
H	-0.51733800	2.69832900	-4.31822200
H	0.93933200	2.05553600	-5.11328500
H	2.97310000	2.54147700	-2.89646700

H	2.78908800	1.00253700	-2.05310000
H	2.76817400	1.04366400	-3.81713100
H	0.76785300	-0.20493700	-3.86834200
H	-0.77729400	0.36763500	-3.25588700
H	0.45648100	-0.23045700	-2.12922700
H	1.36332700	6.41030700	-0.64095100
H	2.43255400	5.39469900	-1.62071700
H	3.11905300	6.54697700	-0.46520600
H	1.10167100	5.93794700	1.88425200
H	2.14675200	4.73493400	2.66740500
H	2.84524100	6.21966100	2.00223200
H	3.87761100	3.46180600	1.33054400
H	4.46431100	4.88981400	0.49714800
H	3.86492800	3.52214500	-0.44521100
H	-1.36485500	6.40902800	0.65108700
H	-2.43411600	5.39149400	1.62881000
H	-3.12057400	6.54608700	0.47559700
H	-1.10309600	5.94128900	-1.87529600
H	-2.14873600	4.74015900	-2.66060400
H	-2.84659200	6.22381600	-1.99236400
H	-3.87910600	3.46433800	-1.32616600
H	-4.46585100	4.89081500	-0.49019700
H	-3.86655400	3.52141300	0.44970500
H	0.77567500	0.36154600	3.25450200
H	-0.45797300	-0.23468300	2.12672300
H	-0.76946500	-0.21223400	3.86584200
H	0.51526500	2.69023400	4.32123200
H	-1.03137800	3.55404100	4.19141300
H	-0.94158500	2.04595800	5.11477600
H	-2.76996300	1.03613300	3.81641900
H	-2.79057700	0.99835300	2.05230700
H	-2.97490700	2.53565900	2.89856300
H	2.16344900	0.96126100	0.22407000
H	-2.16413800	0.96137000	-0.22437900
C	-3.68645900	0.20856300	-1.39763700
C	-4.04808100	-0.70073300	-0.38882600
C	-4.42947400	0.24044000	-2.58218000
C	-5.11997500	-1.56756300	-0.55098500
H	-3.49687600	-0.75325600	0.54555700
C	-5.50423700	-0.63866900	-2.74890700
H	-4.16242100	0.93567300	-3.36379400
C	-5.85557600	-1.55299200	-1.75643000
O	-5.40701200	-2.46332800	0.44769700
H	-6.05794700	-0.60714100	-3.68036200
O	-6.85730900	-2.47483200	-1.85320200
C	-6.63043700	-2.22223300	1.17005200
C	-7.81961400	-2.33641700	-2.88812400
C	-6.72599200	-3.30285400	2.24000200
H	-7.49485900	-2.26295500	0.50175300
H	-6.58582100	-1.23731500	1.65332500
C	-8.90693800	-3.35817400	-2.62637000
H	-8.24259700	-1.32294900	-2.88683700
H	-7.36960900	-2.52611000	-3.87354000
O	-7.68338500	-2.90081100	3.21625100
H	-5.74523300	-3.41273300	2.72274800
H	-6.99559600	-4.26127500	1.78426400
O	-9.56638700	-3.01251100	-1.42670000
H	-8.45303300	-4.35825400	-2.54805700
H	-9.60502600	-3.36978500	-3.48097900
C	-8.74035600	-3.79826100	3.49822800
C	-10.45500000	-4.00498100	-0.95174900

C	-9.98714600	-3.53392800	2.66830800
H	-8.42640700	-4.84289700	3.36246600
H	-8.99903900	-3.66112600	4.55564000
C	-10.84873300	-3.66710700	0.47156700
H	-9.97760900	-4.99704000	-0.96658700
H	-11.36046200	-4.05645000	-1.58019100
O	-9.74436600	-3.90403600	1.32343200
H	-10.82930800	-4.11493500	3.08555600
H	-10.24967800	-2.46621800	2.73606300
H	-11.16643300	-2.61376600	0.52274200
H	-11.70876300	-4.29209800	0.76760000
C	3.68511200	0.20604700	1.39664900
C	4.04839200	-0.70000900	0.38551400
C	4.42637100	0.23430200	2.58239200
C	5.12013000	-1.56723700	0.54658100
H	3.49865000	-0.74959400	-0.54989100
C	5.50092700	-0.64526700	2.74800900
H	4.15818700	0.92714800	3.36573800
C	5.85383200	-1.55647300	1.75322300
O	5.40893200	-2.45976000	-0.45451400
H	6.05320100	-0.61661400	3.68040800
O	6.85540200	-2.47863200	1.84871200
C	6.63340100	-2.21586900	-1.17411000
C	7.81591300	-2.34354200	2.88575400
C	6.73266400	-3.29455500	-2.24562700
H	7.49659200	-2.25666000	-0.50422300
H	6.58846500	-1.23016400	-1.65575000
C	8.90342900	-3.36482300	2.62298600
H	8.23915600	-1.33018600	2.88821400
H	7.36409100	-2.53603000	3.86979300
O	7.69177300	-2.88919600	-3.21884100
H	5.75310500	-3.40514100	-2.73068400
H	7.00280200	-4.25325400	-1.79082800
O	9.56529800	-3.01579100	1.42563400
H	8.44937100	-4.36452500	2.54080000
H	9.59986400	-3.37921100	3.47890200
C	8.74853600	-3.78632300	-3.50265800
C	10.45487000	-4.00695200	0.94973800
C	9.99416200	-3.52560400	-2.66986400
H	8.43374500	-4.83124400	-3.37116200
H	9.00887100	-3.64553300	-4.55918400
C	10.85146600	-3.66516700	-0.47184800
H	9.97746400	-4.99905200	0.96089500
H	11.35907100	-4.06014000	1.57985300
O	9.74879400	-3.89968700	-1.32655300
H	10.83662400	-4.10590700	-3.08748500
H	10.25757700	-2.45787900	-2.73380300
H	11.16928500	-2.61169400	-0.51949000
H	11.71208000	-4.28936300	-0.76786900

Point Group= C1. State= 1-A.
HF= -3265.1536909. RMSD=2.004e-09. RMSF=1.306e-06.
Zero-point correction= 1.2422628 (Hartree/Particle)
Sum of electronic and zero-point Energies= -3263.911428

Calculated IR spectrum (unscaled Harmonic frequencies (cm-1)):

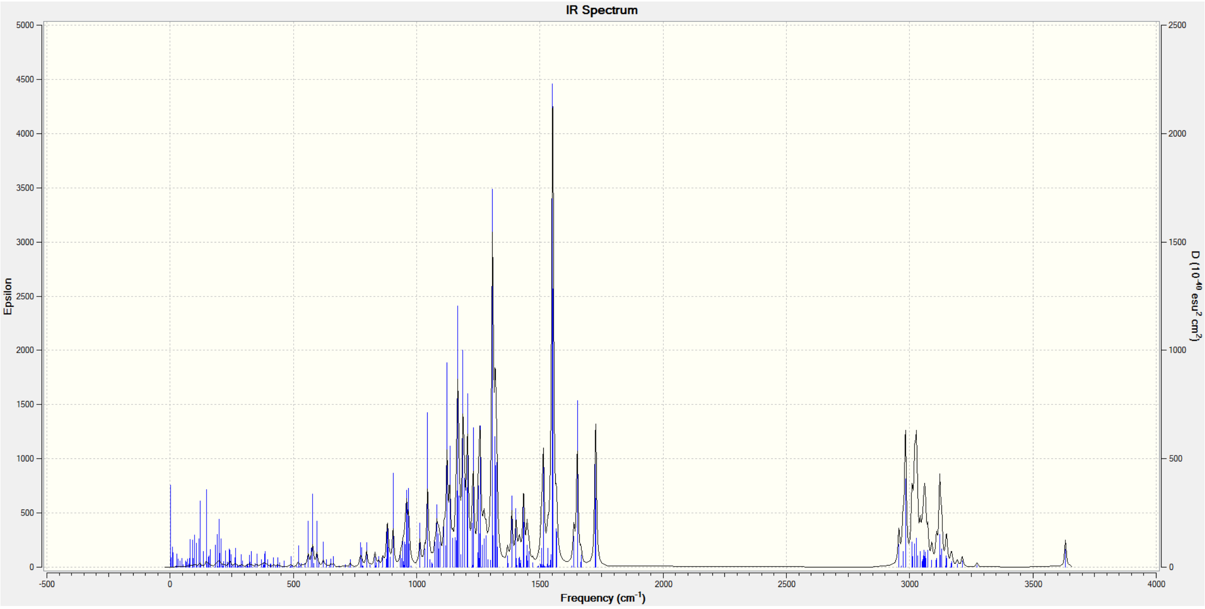

(1,3,5,7-Tetra-tert-butyl-2,6,9-trioxabicyclo[3.3.1]nona-3,7-diene)-4,8-dicarbonic acid-(benzo-15-crown-5)-4'-yl-amide, Compound 7a-b.

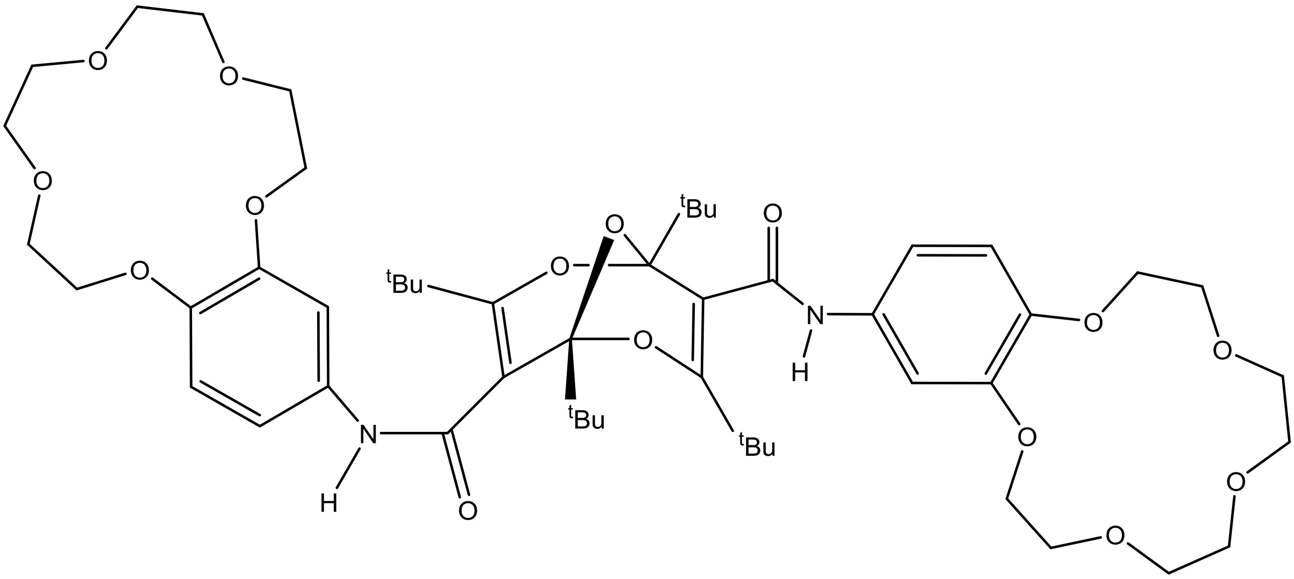

Atom Type	Coordinates (Angstroms) X	Y	Z
O	0.90307800	2.50710400	1.47199100
C	-0.39989000	1.97267400	1.48812100
C	1.48377000	2.51985400	0.19248500
C	-0.87050200	2.06990500	2.98277100
C	2.87761000	3.22636500	0.37949100
C	1.40522100	1.08411200	-0.39868900
C	-1.24325400	2.68254800	0.41835100
C	-0.62107400	3.38849000	-0.55989900
C	-1.23654900	4.30901700	-1.63489900
O	0.73473200	3.40765700	-0.65530300
C	0.50607100	0.20921100	0.12405700

O	-0.29464200	0.55613700	1.18212400
C	0.15213000	-1.24184200	-0.27064400
C	0.89583800	-1.80697400	-1.49290500
C	0.42919000	-2.16178100	0.94535100
C	-1.36143400	-1.29830200	-0.59268400
C	0.20784600	1.41965000	3.88044000
C	-1.02477300	3.55391400	3.37349000
C	-2.19857200	1.32399900	3.23600300
C	2.62831400	4.61934300	1.00776300
C	3.77049700	2.42176300	1.33968500
C	3.60377900	3.44432500	-0.96336500
C	-2.51503800	3.71421100	-2.26695600
C	-1.56680300	5.66889900	-0.97130900
C	-0.22169000	4.53558200	-2.77961200
C	2.01526400	0.88990400	-1.76478400
C	-2.74747400	2.69862100	0.53991700
O	1.44420700	1.35756200	-2.74785600
O	-3.37161700	3.66482000	0.98128200
N	-3.35615400	1.54389300	0.12474300
N	3.22528000	0.25527600	-1.97496600
H	1.96972200	-1.89208300	-1.32815300
H	0.71584900	-1.21538500	-2.39337900
H	0.51873200	-2.81727400	-1.68175900
H	1.49842300	-2.21203700	1.17076100
H	-0.09701900	-1.81488700	1.83671600
H	0.09063500	-3.17839100	0.71906000
H	-1.63639100	-2.31398400	-0.89394100
H	-1.60491200	-0.63097200	-1.42789800
H	-1.96404300	-1.03479100	0.27845400
H	1.16953400	1.92747000	3.79150100
H	0.35147600	0.36458900	3.63239400
H	-0.11429000	1.48146700	4.92486800
H	-0.08503800	4.09593600	3.23941100
H	-1.80003800	4.04741500	2.78378200
H	-1.30446400	3.62082900	4.43035900
H	-3.06188600	1.83308000	2.80785600
H	-2.36406100	1.27261800	4.31720300
H	-2.16599800	0.29944400	2.85714800
H	2.13216300	4.54444500	1.97689700
H	2.01982900	5.24981100	0.35622900
H	3.59255000	5.11551600	1.15777800
H	3.28421500	2.27813300	2.30793000
H	4.03707700	1.44355600	0.93863100
H	4.70264300	2.97104700	1.50890000
H	3.99584200	2.51789700	-1.38338400
H	4.45762400	4.10919300	-0.79658400
H	2.95028300	3.91247900	-1.70253700
H	-3.35702100	3.67537200	-1.57648500
H	-2.33282600	2.71084000	-2.66422300
H	-2.81836500	4.35106300	-3.10429800
H	-2.30896100	5.54921600	-0.17949900
H	-0.66669600	6.11912400	-0.53968400
H	-1.95943800	6.36090000	-1.72494700
H	-0.70119800	5.13384600	-3.56111100
H	0.11194000	3.58897800	-3.21166100
H	0.66197900	5.07710000	-2.43625000
H	-2.73538700	0.83786600	-0.24164500
H	3.45166400	0.30775200	-2.96168500
C	4.17387600	-0.45027100	-1.20132900
C	5.39170700	-0.75483800	-1.83633600
C	3.98548700	-0.87905600	0.11486800

C	6.39042300	-1.47021000	-1.18988500
H	5.58393700	-0.43230800	-2.85550200
C	4.99129700	-1.59577400	0.77380800
H	3.06830600	-0.65149600	0.63776700
C	6.18955600	-1.91299800	0.13674700
O	7.53327800	-1.77638400	-1.87967900
H	4.81250300	-1.92027100	1.79247200
O	7.20032200	-2.64912100	0.68155700
C	8.73544300	-1.10648700	-1.44643800
C	7.22302200	-2.84905600	2.08814000
C	9.83331200	-1.51987300	-2.41999200
H	8.99408900	-1.38771900	-0.42241700
H	8.59267100	-0.01962900	-1.50490700
C	8.55583200	-3.48396800	2.42765900
H	7.11427300	-1.89072800	2.61307300
H	6.40898100	-3.51712700	2.40507300
O	10.93132800	-0.62073400	-2.30116100
H	9.43700600	-1.46130000	-3.44254100
H	10.13853100	-2.55455400	-2.22918700
O	9.57880000	-2.55264000	2.14639700
H	8.68448900	-4.39895700	1.82883800
H	8.55649600	-3.77420300	3.49231700
C	12.19168800	-1.16601100	-1.95924500
C	10.88395800	-3.09509400	2.20760700
C	12.47750200	-1.13086800	-0.46593800
H	12.29387500	-2.19739500	-2.32528000
H	12.94886800	-0.55410000	-2.46530700
C	11.85308400	-2.10558200	1.59427600
H	10.93893800	-4.04378700	1.65175800
H	11.17771000	-3.29680700	3.25169600
O	11.66369300	-2.08439400	0.19245400
H	13.54690900	-1.35180300	-0.29826600
H	12.27930000	-0.11796000	-0.08111300
H	11.68273100	-1.10836300	2.02968200
H	12.88472500	-2.40880500	1.84224100
C	-4.73395800	1.22829500	0.08775500
C	-5.09265900	-0.01055500	-0.47290000
C	-5.74030300	2.06796600	0.57518600
C	-6.42048500	-0.39984500	-0.56295500
H	-4.34038000	-0.69075600	-0.86014600
C	-7.08034800	1.67623200	0.47950700
H	-5.47779900	3.02017100	1.01130300
C	-7.44120400	0.45971900	-0.09864600
O	-6.73624100	-1.59207400	-1.16563900
H	-7.84057300	2.34923600	0.85837700
O	-8.71927300	0.01126100	-0.27911900
C	-7.19038200	-2.63658400	-0.28780600
C	-9.79251000	0.79721700	0.21811100
C	-8.18262100	-3.49045600	-1.05986900
H	-7.67128800	-2.21789000	0.60064400
H	-6.33420400	-3.24704700	0.02926500
C	-11.09083400	0.16169600	-0.23552900
H	-9.75422400	0.84924100	1.31494000
H	-9.74424700	1.82058800	-0.18040400
O	-8.37385600	-4.68071100	-0.29784000
H	-7.76895000	-3.72920800	-2.05253000
H	-9.11875500	-2.94063500	-1.18737800
O	-11.22114000	-1.10961600	0.36274700
H	-11.09109400	0.07768200	-1.33369100
H	-11.92379300	0.82760400	0.05054900
C	-9.47109200	-5.48139800	-0.69883100

C	-12.36835700	-1.82199600	-0.06108000
C	-10.75921400	-5.12153400	0.02849700
H	-9.63051400	-5.42528800	-1.78571200
H	-9.21460900	-6.51861600	-0.45016600
C	-12.22036300	-3.26954400	0.36337500
H	-12.47352300	-1.77856500	-1.15625900
H	-13.28378600	-1.39564600	0.38348600
O	-11.21622000	-3.86393400	-0.43404800
H	-11.51697200	-5.90612500	-0.14739000
H	-10.55712600	-5.08478600	1.11024400
H	-11.94861400	-3.30918800	1.42959000
H	-13.18552400	-3.78999100	0.24083800

```
Point Group= C1. State= 1-A.
HF= -3265.1343346. RMSD=4.696e-09. RMSF=1.942e-06
Zero-point correction= 1.242410 (Hartree/Particle)
Sum of electronic and zero-point Energies= -3263.891924
```

Calculated IR spectrum (unscaled Harmonic frequencies (cm-1)):

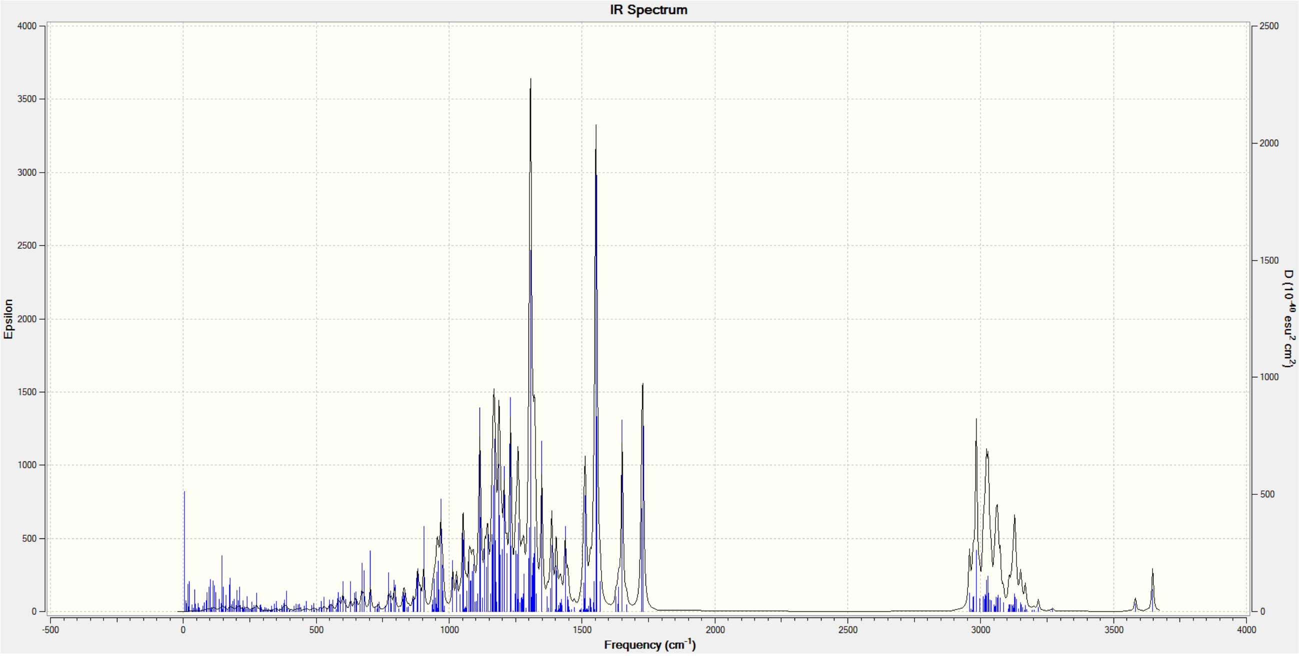

(1,3,5,7-Tetra-tert-butyl-2,6,9-trioxabicyclo[3.3.1]nona-3,7-diene)-4,8-dicarbonic acid-(benzo-15-crown-5)-4’-yl-amide, Compound 7a-c.

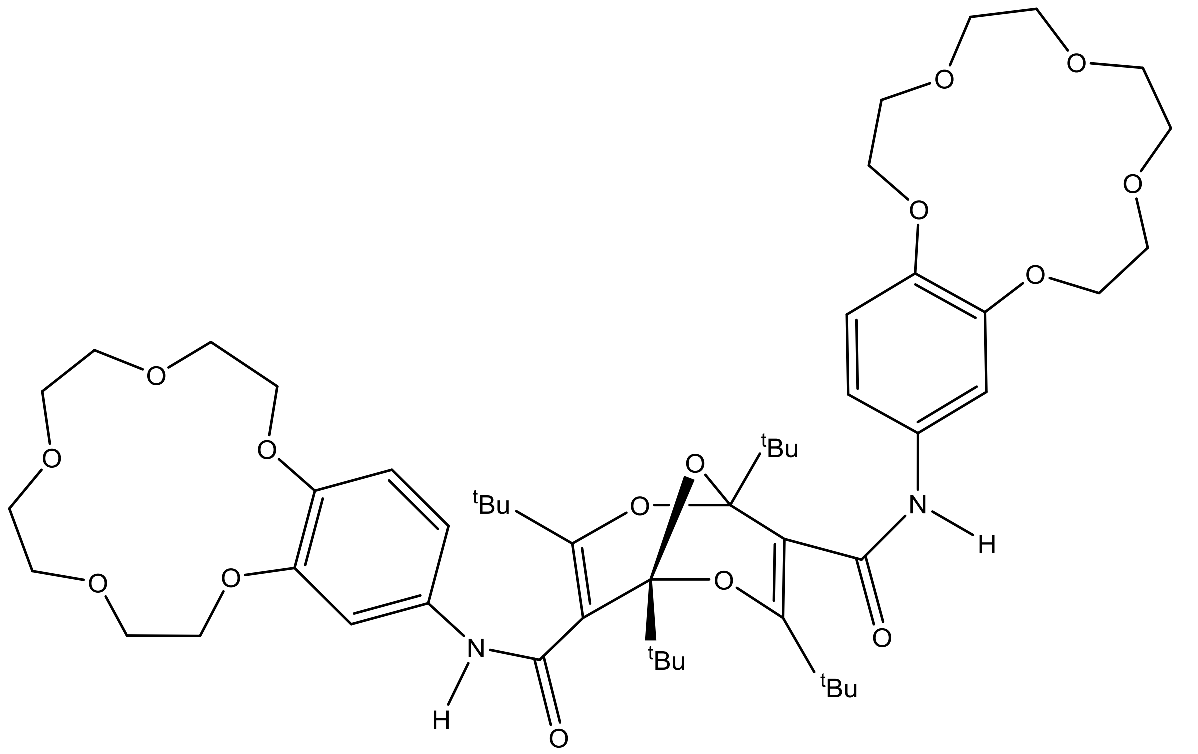

Atom Type	Coordinates (Angstroms) X	Y	Z
O	0.00000000	0.00000000	0.13564930
C	1.00921114	0.61413399	0.90479066
C	-1.00921114	-0.61413399	0.90479066
C	2.09097549	1.08427030	-0.13658278
C	-2.09097549	-1.08427030	-0.13658278
C	-0.34196301	-1.63483099	1.85953552
C	0.34196301	1.63483099	1.85953552
C	-0.93054384	1.39246444	2.26321769
C	-1.78809047	2.03914436	3.36868371
O	-1.67016788	0.38410520	1.70756841
C	0.93054384	-1.39246444	2.26321769
O	1.67016788	-0.38410520	1.70756841
C	1.78809047	-2.03914436	3.36868371
C	1.21070020	-3.31189683	4.01192772
C	3.18385083	-2.38275130	2.79374992
C	1.93812758	-0.97325404	4.48611765
C	2.55317208	-0.15587245	-0.93962300
C	1.48364958	2.09907665	-1.12106726
C	3.33997701	1.69234512	0.53585962
C	-2.55317208	0.15587245	-0.93962300
C	-1.48364958	-2.09907665	-1.12106726
C	-3.33997701	-1.69234512	0.53585962
C	-1.93812758	0.97325404	4.48611765
C	-1.21070020	3.31189683	4.01192772
C	-3.18385083	2.38275130	2.79374992
C	-1.24187305	-2.62796618	2.53688479
C	1.24187305	2.62796618	2.53688479
O	-2.04412211	-2.27052411	3.39833241
O	2.04412211	2.27052411	3.39833241
N	1.26259650	3.96117021	2.16648199
N	-1.26259650	-3.96117021	2.16648199
H	1.13010196	-4.14143060	3.30698931
H	0.23873765	-3.13639026	4.47723299

H	1.89606765	-3.62562826	4.80597307
H	3.11128431	-3.13005727	1.99637925
H	3.68664937	-1.49940155	2.39829675
H	3.80641429	-2.80405336	3.58948517
H	2.64271574	-1.33872398	5.24149806
H	0.97581756	-0.80308510	4.98015561
H	2.29246153	-0.01665147	4.09994018
H	1.71635911	-0.65487551	-1.43154468
H	3.05957338	-0.87990506	-0.29733080
H	3.25995030	0.16511584	-1.71162536
H	0.63636210	1.67149875	-1.66316283
H	1.14795418	3.01053459	-0.62462951
H	2.24292637	2.38620697	-1.85627391
H	3.16269625	2.69683516	0.92081955
H	4.13414347	1.77066805	-0.21397467
H	3.70378167	1.06961169	1.35495181
H	-1.71635911	0.65487551	-1.43154468
H	-3.05957338	0.87990506	-0.29733080
H	-3.25995030	-0.16511584	-1.71162536
H	-0.63636210	-1.67149875	-1.66316283
H	-1.14795418	-3.01053459	-0.62462951
H	-2.24292637	-2.38620697	-1.85627391
H	-3.16269625	-2.69683516	0.92081955
H	-4.13414347	-1.77066805	-0.21397467
H	-3.70378167	-1.06961169	1.35495181
H	-0.97581756	0.80308510	4.98015561
H	-2.29246153	0.01665147	4.09994018
H	-2.64271574	1.33872398	5.24149806
H	-0.23873765	3.13639026	4.47723299
H	-1.13010196	4.14143060	3.30698931
H	-1.89606765	3.62562826	4.80597307
H	-3.80641429	2.80405336	3.58948517
H	-3.68664937	1.49940155	2.39829675
H	-3.11128431	3.13005727	1.99637925
H	1.96647215	4.43951463	2.71740823
H	-1.96647215	-4.43951463	2.71740823
C	0.51891064	4.81690173	1.32107963
C	0.96480812	6.14952007	1.24974611
C	-0.60045598	4.45096769	0.56724963
C	0.31728896	7.09471028	0.46617535
H	1.83699861	6.47516200	1.80932559
C	-1.25633213	5.40061788	-0.22446733
H	-0.96609073	3.43502536	0.58394854
C	-0.82408590	6.72448556	-0.27824342
O	0.77522208	8.38624588	0.47765146
H	-2.12750948	5.08849766	-0.78879641
O	-1.42954577	7.72421849	-0.98286343
C	1.38499905	8.85276839	-0.74304901
C	-2.36252195	7.38005056	-1.99700385
C	1.89810741	10.25930750	-0.45721559
H	0.66425372	8.85905709	-1.56505009
H	2.23280997	8.20525919	-1.00232552
C	-2.68797676	8.65062359	-2.75505701
H	-1.93256149	6.63190516	-2.67632700
H	-3.28501432	6.96828614	-1.56216868
O	2.83480078	10.63149890	-1.46371166
H	2.40549220	10.25802565	0.51685771
H	1.06092359	10.96394561	-0.40695323
O	-1.52200657	9.08496495	-3.42268709
H	-3.04000548	9.41578935	-2.04586400
H	-3.50692353	8.44725821	-3.46620284

C	2.56526936	11.80499392	-2.20676189
C	-1.62948171	10.37860713	-3.98467478
C	1.83360868	11.53281605	-3.51192693
H	1.99333041	12.53221039	-1.61338780
H	3.53508695	12.25817598	-2.44787208
C	-0.24945620	10.85027513	-4.39454302
H	-2.04984152	11.08793144	-3.25506586
H	-2.29061652	10.37038204	-4.86794798
O	0.49936626	11.15016745	-3.23241517
H	1.85371605	12.44534879	-4.13455437
H	2.35874872	10.73773950	-4.06472094
H	0.24412842	10.06557206	-4.98931141
H	-0.34853638	11.74408710	-5.03418538
C	-0.51891064	-4.81690173	1.32107963
C	-0.96480812	-6.14952007	1.24974611
C	0.60045598	-4.45096769	0.56724963
C	-0.31728896	-7.09471028	0.46617535
H	-1.83699861	-6.47516200	1.80932559
C	1.25633213	-5.40061788	-0.22446733
H	0.96609073	-3.43502536	0.58394854
C	0.82408590	-6.72448556	-0.27824342
O	-0.77522208	-8.38624588	0.47765146
H	2.12750948	-5.08849766	-0.78879641
O	1.42954577	-7.72421849	-0.98286343
C	-1.38499905	-8.85276839	-0.74304901
C	2.36252195	-7.38005056	-1.99700385
C	-1.89810741	-10.25930750	-0.45721559
H	-0.66425372	-8.85905709	-1.56505009
H	-2.23280997	-8.20525919	-1.00232552
C	2.68797676	-8.65062359	-2.75505701
H	1.93256149	-6.63190516	-2.67632700
H	3.28501432	-6.96828614	-1.56216868
O	-2.83480078	-10.63149890	-1.46371166
H	-2.40549220	-10.25802565	0.51685771
H	-1.06092359	-10.96394561	-0.40695323
O	1.52200657	-9.08496495	-3.42268709
H	3.04000548	-9.41578935	-2.04586400
H	3.50692353	-8.44725821	-3.46620284
C	-2.56526936	-11.80499392	-2.20676189
C	1.62948171	-10.37860713	-3.98467478
C	-1.83360868	-11.53281605	-3.51192693
H	-1.99333041	-12.53221039	-1.61338780
H	-3.53508695	-12.25817598	-2.44787208
C	0.24945620	-10.85027513	-4.39454302
H	2.04984152	-11.08793144	-3.25506586
H	2.29061652	-10.37038204	-4.86794798
O	-0.49936626	-11.15016745	-3.23241517
H	-1.85371605	-12.44534879	-4.13455437
H	-2.35874872	-10.73773950	-4.06472094
H	-0.24412842	-10.06557206	-4.98931141
H	0.34853638	-11.74408710	-5.03418538

Point Group= C2. State= 1-A.
HF= -3265.1197003. RMSD=3.529e-09. RMSF=3.420e-06.
Zero-point correction= 1.241716 (Hartree/Particle)
Sum of electronic and zero-point Energies= -3263.877985

Calculated IR spectrum (unscaled Harmonic frequencies (cm-1)):

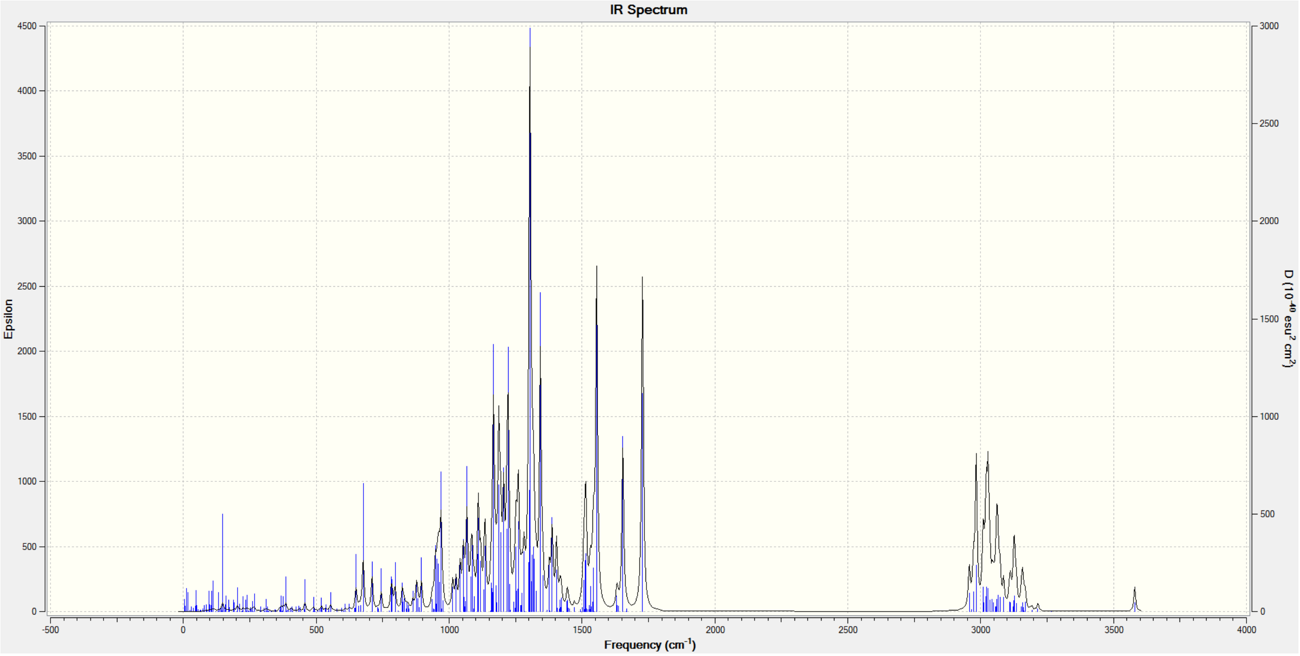

(1,3,5,7-Tetra-tert-butyl-2,6,9-trioxabicyclo[3.3.1]nona-3,7-diene)-4,8-dicarboxylic acid-(dibenzo-18-crown-6)-4'-yl-amide, Compound 7b-a.

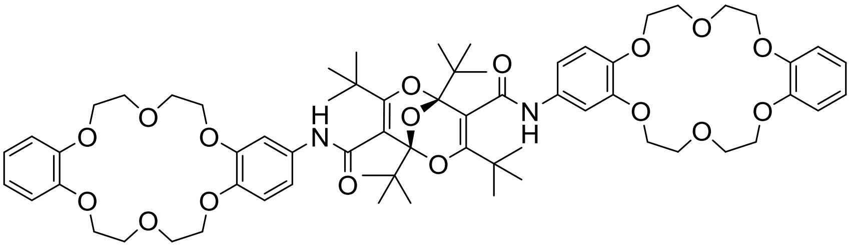

Atom Type	Coordinates (Angstroms) X	Y	Z
O	0.00000038	6.56398544	0.00000002
C	-1.16581475	5.78203700	-0.07366664
C	1.16581541	5.78203687	0.07366669
C	-2.35401032	6.79710106	-0.22601947
C	2.35401109	6.79710078	0.22601951
C	0.99151101	4.74057647	1.18854156
C	-0.99151048	4.74057659	-1.18854152
C	0.22879788	4.55740368	-1.75528850
C	0.58487471	3.78230389	-3.04262851
O	1.34250067	5.12909048	-1.21121509
C	-0.22879737	4.55740370	1.75528854
O	-1.34250009	5.12909064	1.21121513
C	-0.58487429	3.78230396	3.04262855
C	-0.07137221	4.59322432	4.25888066
C	-2.11583939	3.62703636	3.18414425
C	0.03220119	2.36405605	3.05826348
C	-2.21804402	7.87622953	0.87297117
C	-2.27344639	7.46697699	-1.61243952
C	-3.73265878	6.12329987	-0.05541542
C	2.21804492	7.87622927	-0.87297112
C	2.27344725	7.46697672	1.61243956

C	3.73265948	6.12329943	0.05541547
C	-0.03220093	2.36405605	-3.05826344
C	0.07137273	4.59322431	-4.25888061
C	2.11583979	3.62703611	-3.18414420
C	2.20974088	4.00460965	1.68755886
C	-2.20974043	4.00460991	-1.68755882
O	2.75922433	4.24062261	2.76342320
O	-2.75922386	4.24062294	-2.76342316
N	-2.64525717	3.04028842	-0.81534064
N	2.64525751	3.04028811	0.81534068
C	-3.75821338	2.17392719	-0.92342848
C	-3.89331742	1.19701011	0.07978130
C	-4.69726990	2.24267593	-1.95581328
C	-4.95644557	0.29348417	0.06737551
C	-5.76393399	1.34216213	-1.95121957
C	-5.91994696	0.37175718	-0.96689918
O	-5.13319815	-0.68866284	1.00029636
O	-6.95372615	-0.51824298	-1.10356143
C	-4.18348357	-0.81159486	2.05164530
C	-7.87927265	-0.64963836	-0.01972975
C	-4.64520395	-1.88944965	3.01123202
C	-9.00367917	-1.56106126	-0.46419656
O	-4.52468868	-3.15518139	2.40242857
O	-8.48108177	-2.85249657	-0.69340014
C	-4.89782892	-4.21569083	3.26196385
C	-9.46252618	-3.85820839	-0.79098058
C	-4.82254606	-5.52449218	2.50262206
C	-8.78203480	-5.17204873	-1.11839923
O	-5.94140957	-5.59686549	1.63340018
O	-7.92410340	-5.50646040	-0.03736759
C	-5.99499326	-6.61719331	0.72970893
C	-7.06768869	-6.55546657	-0.19587129
C	-5.08976139	-7.67471618	0.66081786
C	-7.18576723	-7.53963263	-1.17490471
C	-5.22419562	-8.66250622	-0.32449318
C	-6.26391589	-8.59315394	-1.24047248
C	3.75821361	2.17392675	0.92342852
C	3.89331754	1.19700966	-0.07978126
C	4.69727014	2.24267538	1.95581332
C	4.95644558	0.29348358	-0.06737547
C	5.76393413	1.34216145	1.95121961
C	5.91994698	0.37175648	0.96689922
O	5.13319805	-0.68866345	-1.00029632
O	6.95372607	-0.51824379	1.10356148
C	4.18348345	-0.81159535	-2.05164526
C	7.87927255	-0.64963929	0.01972979
C	4.64520371	-1.88945020	-3.01123198
C	9.00367897	-1.56106232	0.46419660
O	4.52468829	-3.15518193	-2.40242853
O	8.48108142	-2.85249757	0.69340018
C	4.89782840	-4.21569141	-3.26196381
C	9.46252570	-3.85820951	0.79098062
C	4.82254538	-5.52449275	-2.50262201
C	8.78203417	-5.17204977	1.11839927
O	5.94140888	-5.59686619	-1.63340013
O	7.92410273	-5.50646133	0.03736763
C	5.99499246	-6.61719401	-0.72970888
C	7.06768789	-6.55546741	0.19587134
C	5.08976046	-7.67471678	-0.66081782
C	7.18576632	-7.53963348	1.17490475
C	5.22419457	-8.66250684	0.32449322

C	6.26391486	-8.59315468	1.24047252
H	-0.52657560	5.58897039	4.27827565
H	1.01269499	4.70560938	4.22493793
H	-0.34711155	4.07935240	5.18664198
H	-2.62471690	4.59154810	3.22315848
H	-2.55783338	3.05317872	2.36326609
H	-2.32730133	3.09186188	4.11511452
H	-0.35391233	1.81935527	3.92610566
H	1.11827732	2.37519016	3.14411697
H	-0.24107576	1.79620339	2.16167673
H	-1.29329605	8.44633607	0.77073849
H	-2.23903550	7.43223964	1.87185062
H	-3.05822369	8.57341588	0.79431784
H	-1.29958658	7.93927623	-1.76641809
H	-2.44466588	6.74775178	-2.41670651
H	-3.04134827	8.24438558	-1.68320616
H	-3.99202531	5.46713279	-0.88649650
H	-4.49572164	6.90768797	-0.02121792
H	-3.79600797	5.55773075	0.87725174
H	1.29329702	8.44633592	-0.77073845
H	2.23903635	7.43223937	-1.87185058
H	3.05822467	8.57341552	-0.79431780
H	1.29958750	7.93927608	1.76641813
H	2.44466665	6.74775150	2.41670655
H	3.04134922	8.24438522	1.68320621
H	3.99202593	5.46713232	0.88649654
H	4.49572243	6.90768744	0.02121796
H	3.79600860	5.55773030	-0.87725169
H	-1.11827706	2.37519029	-3.14411693
H	0.24107595	1.79620336	-2.16167669
H	0.35391252	1.81935523	-3.92610562
H	-1.01269446	4.70560949	-4.22493789
H	0.52657623	5.58897032	-4.27827561
H	0.34711201	4.07935236	-5.18664194
H	2.32730167	3.09186161	-4.11511448
H	2.55783372	3.05317842	-2.36326605
H	2.62471742	4.59154779	-3.22315844
H	-2.10829724	2.95984262	0.03597791
H	2.10829757	2.95984237	-0.03597787
H	-3.13977390	1.13625970	0.85668743
H	-4.59285563	2.97979214	-2.73744285
H	-6.50700086	1.37288449	-2.74142169
H	-4.09710731	0.13612882	2.60245374
H	-3.19675015	-1.07309395	1.64689544
H	-8.29270531	0.33530486	0.24347724
H	-7.38644954	-1.07668794	0.85647900
H	-4.01812392	-1.82762898	3.91859303
H	-5.68587555	-1.69171233	3.31490789
H	-9.76249325	-1.57774259	0.33704976
H	-9.48419594	-1.16419589	-1.37365942
H	-5.92111544	-4.07476843	3.64293261
H	-4.21819416	-4.26686566	4.13042759
H	-10.19148784	-3.63797533	-1.59046273
H	-10.02285647	-3.95331148	0.15409269
H	-3.88261589	-5.56270000	1.93606404
H	-4.83226122	-6.36198447	3.21633519
H	-9.55092644	-5.94487348	-1.26864887
H	-8.20221134	-5.07169452	-2.04531846
H	-4.26978046	-7.73470802	1.36647171
H	-7.99761363	-7.49458991	-1.89159887
H	-4.50654550	-9.47605196	-0.36456846

H	-6.37273190	-9.35068590	-2.01038587
H	3.13977401	1.13625933	-0.85668739
H	4.59285596	2.97979160	2.73744289
H	6.50700100	1.37288373	2.74142173
H	4.09710730	0.13612833	-2.60245370
H	3.19675000	-1.07309433	-1.64689540
H	8.29270532	0.33530388	-0.24347719
H	7.38644939	-1.07668881	-0.85647896
H	4.01812369	-1.82762945	-3.91859299
H	5.68587533	-1.69171300	-3.31490785
H	9.76249304	-1.57774374	-0.33704972
H	9.48419578	-1.16419701	1.37365946
H	5.92111493	-4.07476913	-3.64293256
H	4.21819364	-4.26686616	-4.13042755
H	10.19148739	-3.63797653	1.59046278
H	10.02285598	-3.95331266	-0.15409265
H	3.88261521	-5.56270046	-1.93606400
H	4.83226045	-6.36198504	-3.21633515
H	9.55092571	-5.94487461	1.26864891
H	8.20221072	-5.07169549	2.04531850
H	4.26977953	-7.73470852	-1.36647167
H	7.99761272	-7.49459085	1.89159892
H	4.50654436	-9.47605249	0.36456850
H	6.37273077	-9.35068665	2.01038591

Point Group= C2. State= 1-A.
HF= -3877.6842961. RMSD=1.793e-09. RMSF=2.259e-06.
Zero-point correction= 1.4121496 (Hartree/Particle)
Sum of electronic and zero-point Energies= -3876.272146

Calculated IR spectrum (unscaled Harmonic frequencies (cm-1)):

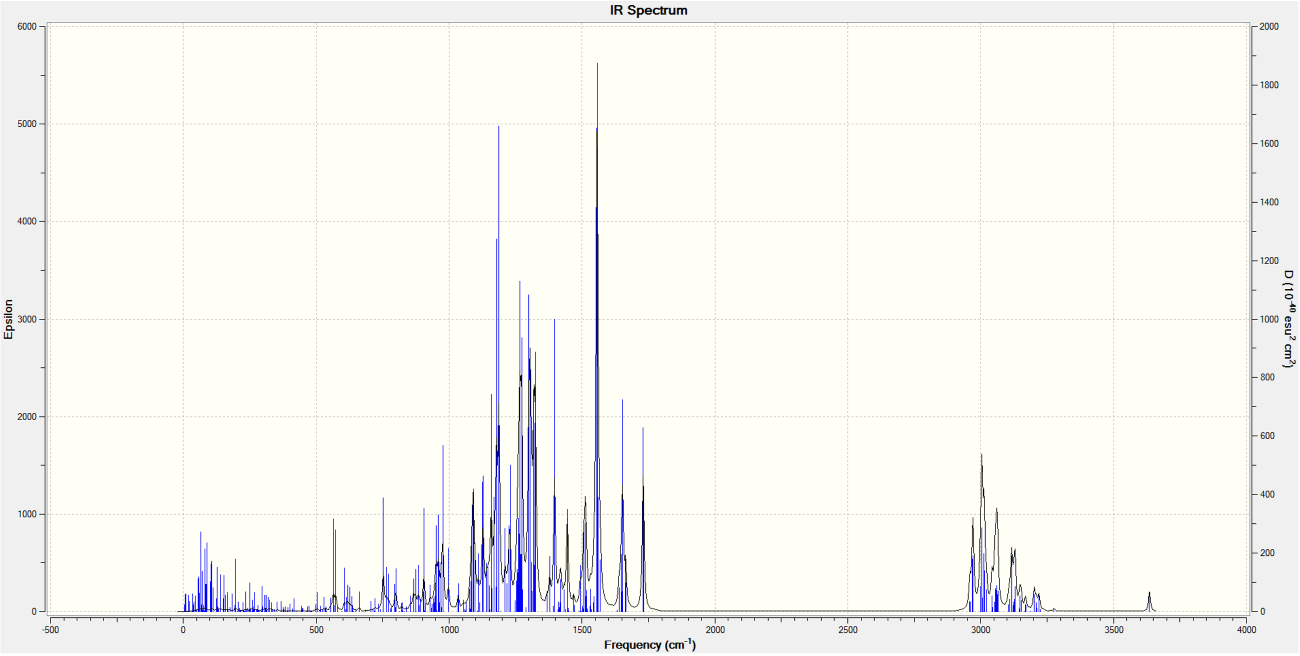

(1,3,5,7-Tetra-tert-butyl-2,6,9-trioxabicyclo[3.3.1]nona-3,7-diene)-4,8-dicarboxylic acid-(dibenzo-18-crown-6)-4’-yl-amide, Compound 7b-b.

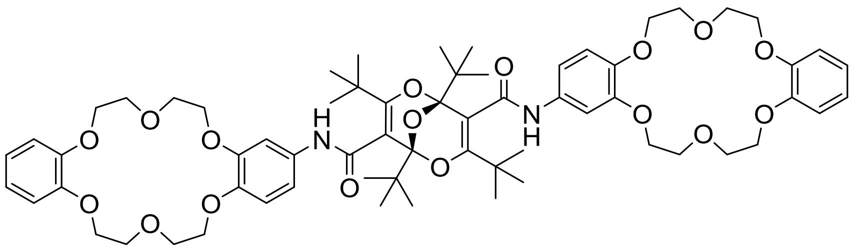

Atom Type	Coordinates (Angstroms) X	Y	Z
O	-0.68278619	-4.25562290	1.06600548
C	0.55280651	-3.58761327	1.16018471
C	-1.26868594	-4.18406922	-0.20812496
C	1.00824428	-3.80253525	2.64903042
C	-2.53827419	-5.11181925	-0.13601451
C	-1.38596147	-2.68636213	-0.59895601
C	1.47694851	-4.06868608	0.03890273
C	0.93715767	-4.67392021	-1.04923811
C	1.65233849	-5.31228330	-2.25834632
O	-0.40910665	-4.82901631	-1.16920862
C	-0.53794967	-1.79234184	-0.02646468
O	0.31572023	-2.15982044	0.98417799
C	-0.24675215	-0.32473346	-0.43224017
C	-1.46945057	0.48754856	-0.90582618
C	0.36794930	0.44937005	0.75757851
C	0.76697769	-0.36903834	-1.60602453
C	-0.15179398	-3.37894235	3.57995926
C	1.32071662	-5.29764779	2.86511366
C	2.24344514	-2.96449833	3.04286632
C	-2.06601657	-6.53733109	0.24149807
C	-3.50973577	-4.62779955	0.95369752
C	-3.26708519	-5.20588254	-1.49206014
C	2.81431874	-4.43479335	-2.77701533
C	2.18965268	-6.69998088	-1.83083871
C	0.65624711	-5.49274562	-3.42736532
C	-2.11920215	-2.36025564	-1.87277618
C	2.96483264	-3.88987862	0.19003321
O	-1.58510721	-2.56690924	-2.96215596
O	3.74158235	-4.83039819	0.36020498
N	3.37261497	-2.58179289	0.12942426
N	-3.41131046	-1.86757654	-1.88039677
C	-4.36337845	-1.43277808	-0.92673913
C	-5.65289462	-1.16372928	-1.43208665
C	-4.11851628	-1.23366944	0.42934283
C	-6.67173452	-0.70295365	-0.60566177
C	-5.14905337	-0.78244572	1.26721131
C	-6.42041882	-0.50854304	0.77737006
O	-7.93565686	-0.41973847	-1.01606858
O	-7.47680656	-0.06435673	1.51295959
C	-8.25426894	-0.55810560	-2.39405984
C	-7.27628123	0.17356510	2.89861946
C	-9.68439824	-0.10138563	-2.59658913
C	-8.56155844	0.73176619	3.47400086
O	-9.73560857	1.30539653	-2.49756510
O	-8.71679659	2.06665060	3.04195314
C	-11.04645314	1.82330542	-2.59939732
C	-9.93068732	2.65201191	3.46513909
C	-11.00494461	3.32457978	-2.40130325
C	-10.03808128	4.04686583	2.88423378
O	-10.77537721	3.58545317	-1.02566378

O	-10.31725756	3.93041881	1.49790300
C	-10.65821540	4.88374229	-0.62497994
C	-10.40692643	5.07268506	0.75873583
C	-10.77199844	5.99400184	-1.45891992
C	-10.27779566	6.36562004	1.26157960
C	-10.63869925	7.28995984	-0.94075840
C	-10.39292751	7.47461497	0.41155557
C	4.66473486	-2.04052365	0.32483491
C	4.76183294	-0.63362339	0.35154171
C	5.81388218	-2.81269998	0.48116286
C	5.98924206	-0.00733818	0.53050577
C	7.05243322	-2.17611995	0.64463069
C	7.16597869	-0.79052107	0.65453920
O	6.17613006	1.33821531	0.61056194
O	8.32613324	-0.08447664	0.76982595
C	5.04703279	2.19344893	0.54638238
C	9.53801037	-0.79846127	0.94581405
C	5.48964131	3.58990610	0.93269982
C	10.68778668	0.18361366	0.83765618
O	6.36318473	4.10153694	-0.05006866
O	10.62118249	1.10344040	1.90822966
C	6.87572386	5.37687389	0.27826388
C	11.77628775	1.89353371	2.09527881
C	7.99884737	5.72600730	-0.67614892
C	12.23520506	2.70993361	0.89198573
O	9.14598748	4.97626641	-0.30306897
O	11.18739115	3.57486167	0.48740847
C	10.28137549	5.12502075	-1.04129337
C	11.39647623	4.35937528	-0.61176514
C	10.40995439	5.95760119	-2.15178137
C	12.59985216	4.45222907	-1.30846815
C	11.62579714	6.04074772	-2.84408004
C	12.71516979	5.29211131	-2.42498501
H	-2.26027830	0.52104967	-0.15430888
H	-1.89212215	0.11591498	-1.83822339
H	-1.14481503	1.51627997	-1.09211042
H	-0.32151235	0.48903859	1.60670162
H	1.30123778	0.00868231	1.11093348
H	0.57196510	1.47765158	0.44298482
H	1.02771237	0.65185110	-1.90650554
H	0.34044198	-0.88923896	-2.46683591
H	1.69559175	-0.88064530	-1.33941365
H	-1.04926676	-3.97556754	3.41096190
H	-0.40659616	-2.32483176	3.43885395
H	0.15747041	-3.51672150	4.62088699
H	0.44794631	-5.92061916	2.65430278
H	2.14714937	-5.63187876	2.23304982
H	1.60604027	-5.45931480	3.91005332
H	3.16917939	-3.32937409	2.59825398
H	2.36652711	-3.02777313	4.12889212
H	2.11936049	-1.90900068	2.78776509
H	-1.51591437	-6.54543721	1.18414742
H	-1.42900592	-6.96833863	-0.53330313
H	-2.94474025	-7.18018009	0.35588279
H	-3.02507480	-4.59476368	1.93264709
H	-3.91902831	-3.64082966	0.73843949
H	-4.35170691	-5.32513575	1.01890578
H	-3.82795878	-4.30093282	-1.72803643
H	-3.98737706	-6.02928745	-1.44684405
H	-2.57226893	-5.40910563	-2.30975863
H	3.64522190	-4.36923253	-2.07570519

H	2.47053122	-3.42414648	-3.01814543
H	3.20547769	-4.88019762	-3.69768350
H	2.92373380	-6.60426305	-1.02891369
H	1.37317420	-7.34165274	-1.48259852
H	2.66045111	-7.19299537	-2.68888631
H	1.20045590	-5.87978998	-4.29483021
H	0.18163194	-4.54712585	-3.70159120
H	-0.13431700	-6.20470448	-3.18120753
H	2.62337774	-1.90883387	0.05593532
H	-3.71780045	-1.77932510	-2.84264437
H	-5.84639478	-1.32305801	-2.48677851
H	-3.14412424	-1.43347624	0.84977951
H	-4.93119988	-0.64257273	2.31890447
H	-8.15588932	-1.60826719	-2.70627702
H	-7.58445502	0.05572285	-3.01093693
H	-6.46289070	0.89458212	3.05635561
H	-7.01804210	-0.76278612	3.41532506
H	-10.02529220	-0.43932036	-3.59120577
H	-10.32937445	-0.57700119	-1.84141554
H	-9.40838096	0.10796752	3.14789511
H	-8.50866388	0.67831284	4.57581346
H	-11.70868231	1.37385484	-1.84332366
H	-11.47522457	1.60898547	-3.59414673
H	-9.96838983	2.72306393	4.56651622
H	-10.79435868	2.05294281	3.13707601
H	-10.20462955	3.75303452	-3.01973087
H	-11.96293929	3.76047250	-2.72191904
H	-10.84455318	4.59329504	3.39574008
H	-9.09510024	4.58548317	3.04902134
H	-10.96343546	5.86085795	-2.51690549
H	-10.08592701	6.52128771	2.31643418
H	-10.72944498	8.14216359	-1.60700494
H	-10.28795717	8.47393440	0.82235380
H	3.86347326	-0.03888127	0.22977998
H	5.74356590	-3.88980369	0.47519366
H	7.93499714	-2.79300895	0.76496047
H	4.26980400	1.86725994	1.25290414
H	4.61916433	2.19368258	-0.46600367
H	9.66051718	-1.56166557	0.16314561
H	9.55107931	-1.29840649	1.92451403
H	4.59093809	4.22435030	1.03472101
H	5.98684493	3.55082474	1.91430481
H	11.63094828	-0.39048868	0.86363174
H	10.62517060	0.69763220	-0.13166120
H	7.26356991	5.39419160	1.30824150
H	6.08759776	6.14646095	0.20167937
H	12.63477883	1.26849666	2.40253222
H	11.53567654	2.56955912	2.92061012
H	7.69373287	5.48979566	-1.70444626
H	8.20929635	6.80395904	-0.61409321
H	13.12685876	3.28209704	1.19201062
H	12.53046857	2.05683140	0.05880972
H	9.56465114	6.54470456	-2.48969544
H	13.45874370	3.87509582	-0.98786108
H	11.70369587	6.69383254	-3.70770924
H	13.66159215	5.34932973	-2.95354231

Point Group= C1. State= 1-A.
HF= -3877.6652277. RMSD=1.379e-09. RMSF=1.042e-06
Zero-point correction= 1.412655 (Hartree/Particle)
Sum of electronic and zero-point Energies= -3876.252573

Calculated IR spectrum (unscaled Harmonic frequencies (cm-1)):

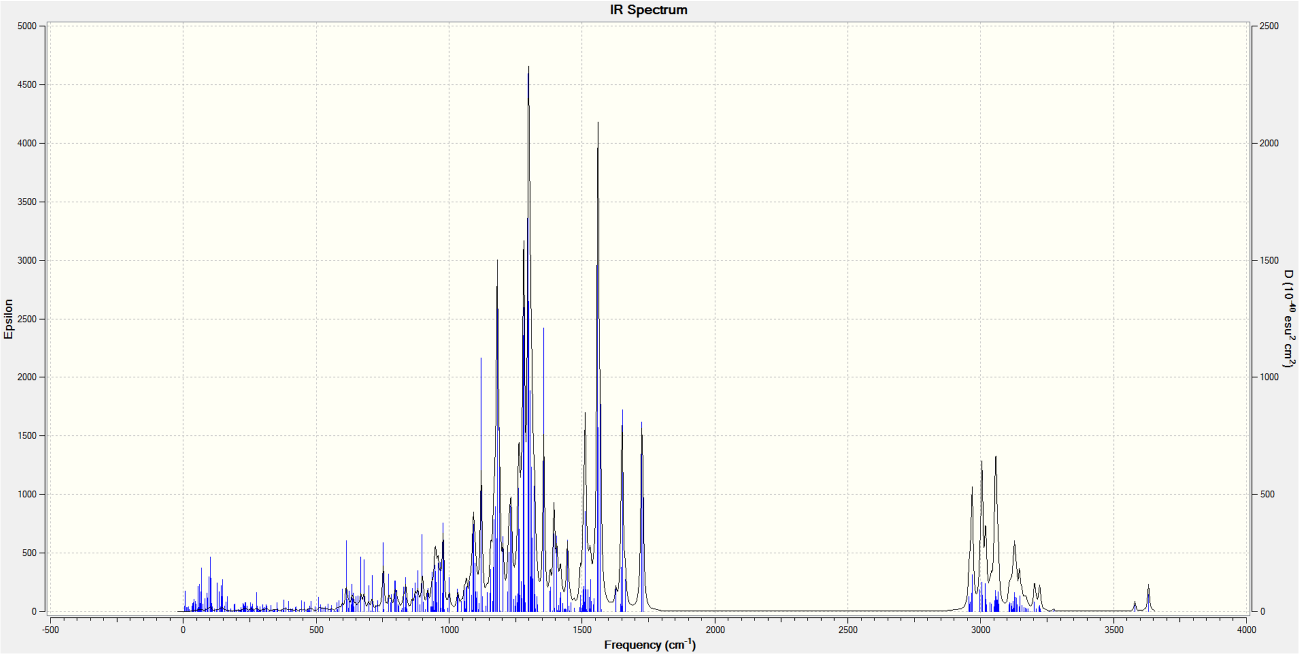

(1,3,5,7-Tetra-tert-butyl-2,6,9-trioxabicyclo[3.3.1]nona-3,7-diene)-4,8-dicarboxylic acid-(dibenzo-18-crown-6)-4'-yl-amide, Compound 7b-c.

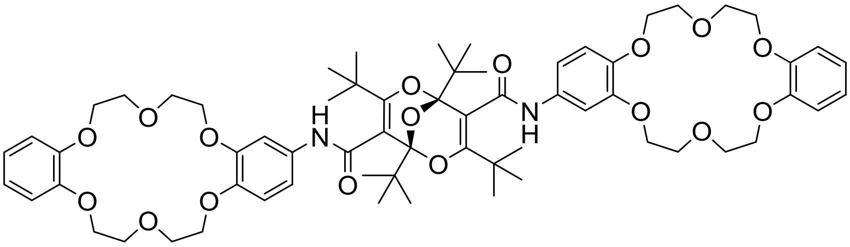

Atom Type	Coordinates (Angstroms) X	Y	Z
O	-0.00006600	-0.00024200	-0.62989200
C	-0.49871600	1.07014500	0.13923900
C	0.49869800	-1.07082200	0.13889700
C	-0.84871900	2.19621200	-0.90197400
C	0.84853000	-2.19663400	-0.90264700
C	1.58506800	-0.52144800	1.09570400
C	-1.58494300	0.52053100	1.09606700
C	-1.48767400	-0.77255700	1.49590700
C	-2.23321900	-1.55430200	2.59620500
O	-0.56788200	-1.61912100	0.93886000
C	1.48786600	0.77154500	1.49586800
O	0.56798900	1.61824600	0.93916800
C	2.23359400	1.55303300	2.59622400
C	3.44523900	0.84172800	3.22367500
C	2.71903700	2.90546000	2.01935900
C	1.20359800	1.81684100	3.72563000
C	0.43756000	2.53394400	-1.69449000
C	-1.91021500	1.69675000	-1.89846100
C	-1.33357900	3.49681600	-0.22723400

C	-0.43787000	-2.53412700	-1.69506900
C	1.90991400	-1.69696100	-1.89914700
C	1.33342800	-3.49742400	-0.22829500
C	-3.44481400	-0.84318200	3.22396000
C	-2.71867400	-2.90664200	2.01914700
C	-1.20305100	-1.81827500	3.72541900
C	2.45976100	-1.52565800	1.79083900
C	-2.45955900	1.52455800	1.79155400
O	2.00824100	-2.25289100	2.67470600
O	-2.00793700	2.25160000	2.67552200
N	-3.77767900	1.72270200	1.42232800
N	3.77781300	-1.72377200	1.42136600
C	-4.70460100	1.13940400	0.52609400
C	-5.91339700	1.84774000	0.35503700
C	-4.52406200	-0.05745200	-0.16281100
C	-6.91760000	1.37305600	-0.48051300
C	-5.53351300	-0.53278000	-1.01316500
C	-6.72922100	0.15443600	-1.18269400
O	-8.10366500	1.99950800	-0.70173100
O	-7.76215600	-0.23200600	-1.98293000
C	-8.36484200	3.22158500	-0.02633800
C	-7.65341200	-1.47282000	-2.66419000
C	-9.75470200	3.68671800	-0.40907400
C	-8.94368500	-1.71323200	-3.42039800
O	-10.70822200	2.86890600	0.23390900
O	-9.96364000	-2.04814400	-2.50359800
C	-12.03910800	3.18248800	-0.12134500
C	-11.22522000	-2.21814500	-3.11527700
C	-12.97778700	2.20365500	0.55333300
C	-12.26320400	-2.50006600	-2.04861100
O	-12.86395800	0.95220100	-0.10539800
O	-12.52008700	-1.29433100	-1.34597700
C	-13.58931900	-0.09810400	0.37444800
C	-13.40074100	-1.32924500	-0.30544900
C	-14.47576900	-0.03634000	1.44757800
C	-14.10501800	-2.45695700	0.11136800
C	-15.17741300	-1.17865400	1.85792100
C	-14.99309000	-2.38195900	1.19364800
C	4.70469600	-1.14016700	0.52529000
C	5.91361500	-1.84828100	0.35419500
C	4.52400300	0.05676400	-0.16344100
C	6.91779900	-1.37329900	-0.48120900
C	5.53343600	0.53239500	-1.01364700
C	6.72927000	-0.15459500	-1.18320600
O	8.10398400	-1.99952300	-0.70243800
O	7.76220100	0.23215800	-1.98329600
C	8.36535800	-3.22159700	-0.02711600
C	7.65326700	1.47304300	-2.66439900
C	9.75538900	-3.68635700	-0.40968200
C	8.94354600	1.71380000	-3.42048800
O	10.70860700	-2.86839400	0.23355700
O	9.96336400	2.04881000	-2.50357200
C	12.03962800	-3.18157600	-0.12154800
C	11.22496500	2.21911500	-3.11512500
C	12.97794300	-2.20257700	0.55339300
C	12.26279100	2.50116900	-2.04834100
O	12.86387200	-0.95107100	-0.10519700
O	12.51984500	1.29543600	-1.34576800
C	13.58898500	0.09933600	0.37480200
C	13.40031300	1.33046900	-0.30508500
C	14.47526000	0.03768800	1.44808300

C	14.10432500	2.45828700	0.11188800
C	15.17663800	1.18010900	1.85858400
C	14.99222400	2.38340400	1.19431900
H	4.24507200	0.65876800	2.50369900
H	3.16980500	-0.09824600	3.70570000
H	3.85113500	1.49650100	4.00165100
H	3.45008200	2.75764900	1.21723700
H	1.89145100	3.49904800	1.62912600
H	3.20803700	3.48000500	2.81260700
H	1.65766900	2.46475100	4.48372300
H	0.92338500	0.87651500	4.21142400
H	0.29330700	2.28755300	3.35261100
H	0.85546900	1.65139700	-2.18213700
H	1.20330200	2.96800400	-1.04779300
H	0.19281300	3.26732600	-2.46977400
H	-1.55660200	0.82258200	-2.45076500
H	-2.85126400	1.43554500	-1.41247500
H	-2.12266900	2.48944700	-2.62381600
H	-2.35554700	3.41901600	0.14478200
H	-1.32106000	4.29994500	-0.97149200
H	-0.68696900	3.78984100	0.60200100
H	-0.85581200	-1.65145100	-2.18245300
H	-1.20354000	-2.96830200	-1.04836400
H	-0.19326100	-3.26734100	-2.47055600
H	1.55628800	-0.82260000	-2.45113700
H	2.85106000	-1.43597900	-1.41322800
H	2.12219300	-2.48945800	-2.62477100
H	2.35544300	-3.41975100	0.14362000
H	1.32079500	-4.30036500	-0.97275400
H	0.68690400	-3.79063700	0.60094100
H	-4.24476900	-0.66015200	2.50413800
H	-3.16937100	0.09673100	3.70609900
H	-3.85054700	-1.49811200	4.00189000
H	-3.44983000	-2.75871500	1.21714800
H	-1.89111300	-3.50012100	1.62869600
H	-3.20754600	-3.48135600	2.81235200
H	-1.65698500	-2.46635400	4.48345200
H	-0.92282400	-0.87802700	4.21135300
H	-0.29278800	-2.28887300	3.35218600
H	-4.16104300	2.47417300	1.98414700
H	4.16124000	-2.47535500	1.98299200
H	-6.05170200	2.78203100	0.88709700
H	-3.60649300	-0.61757300	-0.06534000
H	-5.36392200	-1.46413500	-1.53941800
H	-7.62966600	3.98574000	-0.31907900
H	-8.31214900	3.08367100	1.06196500
H	-7.48521700	-2.29321800	-1.95344200
H	-6.81365600	-1.44992600	-3.37489100
H	-9.87033200	4.74158800	-0.10298300
H	-9.86541800	3.64075300	-1.50371300
H	-9.20514100	-0.80836400	-3.99105000
H	-8.78156800	-2.53178100	-4.14400200
H	-12.17999500	3.13027000	-1.21211600
H	-12.30029400	4.20501500	0.20387400
H	-11.20645400	-3.06629100	-3.82239700
H	-11.51629100	-1.31776800	-3.67854300
H	-12.71028100	2.11224300	1.61471800
H	-14.00833700	2.58330000	0.48601800
H	-13.18257400	-2.87131600	-2.52568000
H	-11.88773300	-3.27620100	-1.36802600
H	-14.62683300	0.89853100	1.97393000

H	-13.96765300	-3.40223900	-0.39974500
H	-15.86221300	-1.11047500	2.69744800
H	-15.53102800	-3.27224000	1.50418400
H	6.05203700	-2.78263400	0.88611500
H	3.60633800	0.61672500	-0.06594000
H	5.36372900	1.46381200	-1.53975200
H	7.63040900	-3.98590400	-0.32003400
H	8.31247000	-3.08379400	1.06119200
H	7.48487800	2.29331500	-1.95355100
H	6.81355800	1.45008600	-3.37515300
H	9.87121900	-4.74123700	-0.10369900
H	9.86627400	-3.64023400	-1.50429800
H	9.20522400	0.80905100	-3.99122600
H	8.78131900	2.53240200	-4.14400800
H	12.18065600	-3.12917500	-1.21229100
H	12.30104100	-4.20407500	0.20357500
H	11.20608900	3.06731100	-3.82218200
H	11.51627100	1.31884000	-3.67843100
H	12.71025000	-2.11137400	1.61474900
H	14.00860800	-2.58193000	0.48618800
H	13.18213200	2.87263700	-2.52529600
H	11.88710100	3.27718100	-1.36773600
H	14.62638600	-0.89717200	1.97443800
H	13.96689200	3.40356000	-0.39922100
H	15.86130300	1.11202000	2.69822800
H	15.52995800	3.27376700	1.50497600

Point Group= C1. State= 1-A.
HF= -3877.6577297. RMSD=3.538e-09. RMSF=9.346e-07
Zero-point correction= 1.412393 (Hartree/Particle)
Sum of electronic and zero-point Energies= -3876.245337

Calculated IR spectrum (unscaled Harmonic frequencies (cm-1)):

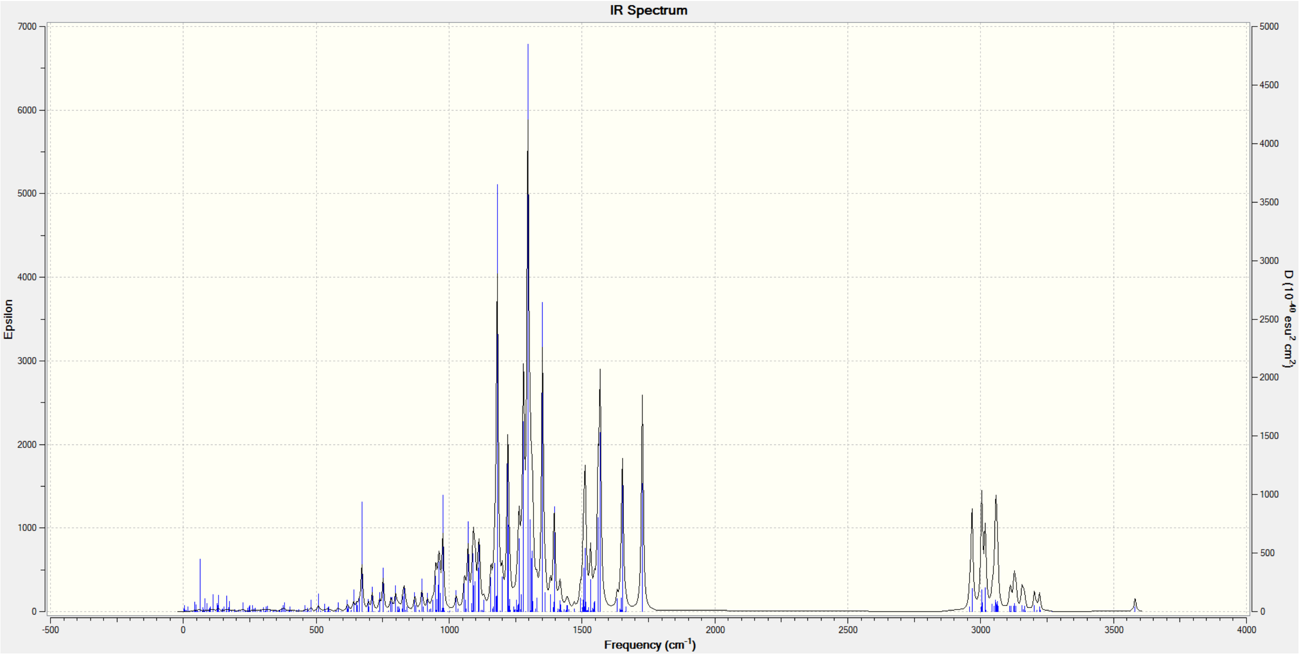

References

1. Kremsner, J.; Wallfisch, B. C.; Belaj, F.; Uray, G.; Kappe, C. O.; Wentrup, C.; Kollenz, G. *Eur. J. Org. Chem.* **2008,** 3382–3388.
2. Smounig, R.; Leber, S.; Kvaskoff, D.; Kollenz, G.; Wentrup, C. *RSC Adv.* **2012,** *2,* 7743–7746.
3. Smounig, R.; Kappe, C. O.; Wentrup, C.; Kollenz, G. *Monatsh. Chem.* **2003,** *134,* 509–518.
4. Smounig, R. Ph.D. Thesis, Karl Franzens Universität Graz, Austria; 1998.
5. Heilmayer, W.; Smounig, R.; Gruber, K.; Fabian, W. M. F.; Reidlinger, C.; Kappe, C. O.; Wentrup, C.; Kollenz, G. *Tetrahedron* **2004,** *60,* 2857–2867.
6. *Gaussian 09*, Revision A.02; Gaussian, Inc.: Pittsburgh, 2009.
7. Wong, M. W. *Chem. Phys. Lett.* **1996**, *256*, 391.
